# Facile Synthesis of Functionalized Spiropyrrolizidine Oxindoles via a Three-Component Tandem Cycloaddition Reaction

**DOI:** 10.3390/molecules16108745

**Published:** 2011-10-19

**Authors:** Yong-Mei Xie, Yu-Qin Yao, Hong-Bao Sun, Ting-Ting Yan, Jie Liu, Tai-Ran Kang

**Affiliations:** 1 State Key Laboratory of Biotherapy, West China Hospital, West China Medical School, Sichuan University, Chengdu, 610041, China; Email: xieym@scu.edu.cn (Y.M.X.); yuqin_yao@163.com (Y.Q.Y.); 773770248@qq.com (H.B.S.); 121968698@qq.com (T.T.Y.); 2 Chemical Synthesis and Pollution Control Key Laboratory of Sichuan Province, College of Chemistry and Chemical Engineering, China West Normal University, Nanchong, 637002, China

**Keywords:** spiropyrrolizidine oxindoles, cycloaddition, isatin

## Abstract

An efficient synthesis of functionalized spiropyrrolizidine oxindoles via a three-component tandem cycloaddition has been achieved. This strategy can provide direct and rapid access to spiropyrrolizidine oxindoles in high yields (up to 99%) with excellent diastereoselectivities (up to 99:1 dr). The features of this procedure are the following: mild reaction conditions, high yields, high diastereoselectivities, one-pot procedure and operational simplicity.

## 1. Introduction

Spirocyclic oxindoles are valuable synthetic intermediates and constitute the core units of many pharmacological agents and alkaloids [1,2,3]. These compounds have attracted much attention from synthetic chemists due to their diverse biological activities including antimycobacterial [4,5,6,7,8,9], antitumor [10,11,12,13,14], antimicrobial [15], antibacterial [16,17], antifungal [18,19], antiviral [20,21], and local anesthetic [22] properties. Hence, a number of synthetic routes have been developed for the preparation of these structural frameworks [23,24,25,26,27,28,29,30,31,32,33]. 1,3-Dipolar cycloaddition provides an efficient approach for the synthesis of ﬁve-membered heterocycles [34,35] and spiro-heterocycles, such as poly functionalized pyrrolidines [36,37,38,39], pyrazolidines and pyrrolizines [40,41], which widely occur in natural products and biologically active compounds. Although there are reports of synthesis of these substituted heterocycles, the development of synthetically important functionalized new spiro-heterocycles is still a challenge and has become a much attempted research endeavor.

Spiropyrrolizidine oxindoles are important synthetic targets and several reports of such syntheses exist [42,43]. To the best of our knowledge, however, there are no reports concerning the synthesis of spiropyrrolizidine oxindoles **4** containing two ester groups or two amide groups, which could possess some interesting biological activities. Herein, we report a three-component tandem cycloaddition reaction between substituted isatins, L-proline and maleates (maleamide) that produces such structures.

## 2. Results and Discussion

**Scheme 1:**
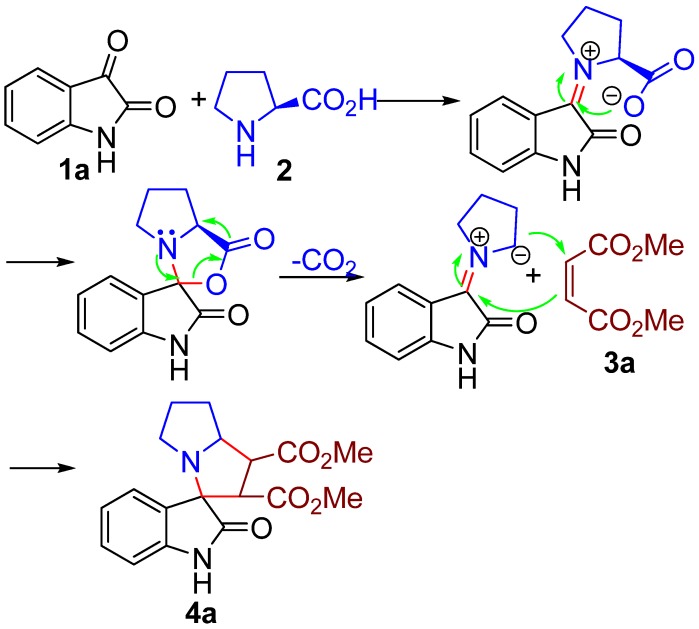
Possible reaction mechanism for the synthesis of spiropyrrolidine oxindole.

From the mechanistic perspective, the azomethine ylides, a class of powerful reagents, have emerged in a number of 1,3-dipolar cycloaddition reactions. In combination with the experiences in previous work, we envisaged that an azomethine ylide could be generated *in situ* from isatin (**1a**) and L-proline (**2**), and then trapped with dimethyl maleate (**3a**) acting as dipolarophile to afford spiropyrrolizidine oxindole **4a**. Hence, the 1,3-dipolar cycloaddition reaction would be facilitated (Scheme 1).

**Figure 1:**
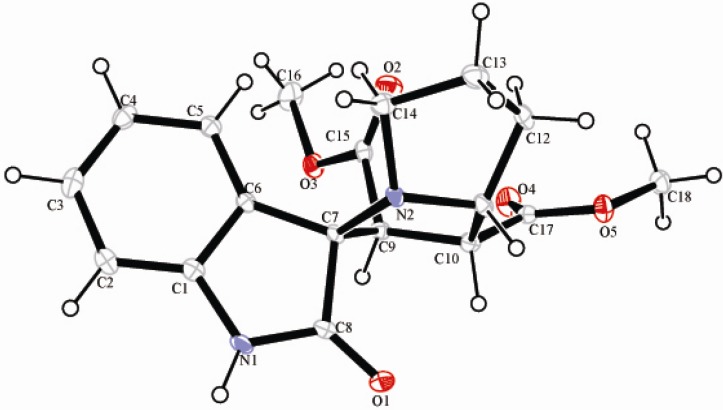
X-ray structure of racemiccompound **4a**.

**Scheme 2:**
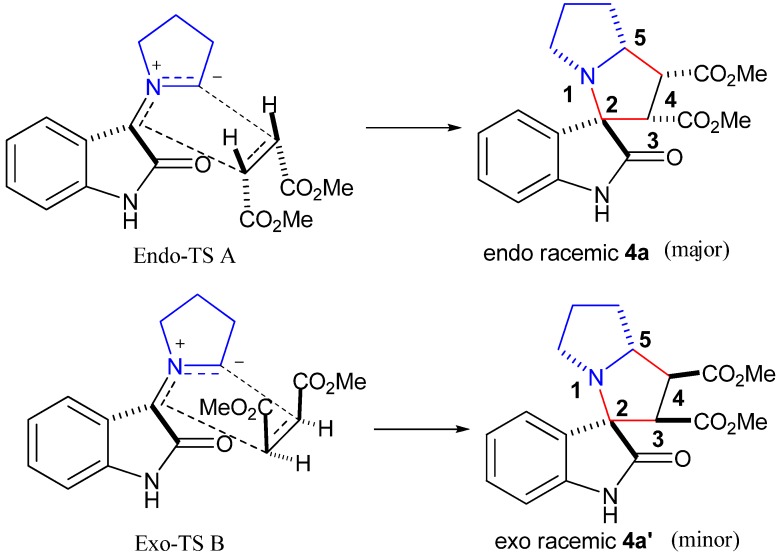
Stereochemistry of cycloadducts differing in their relative configuration.

In light of the above considerations, the reaction in methanol at 60 °C of dimethyl maleate with azomethine ylide (generated *in situ* by decarboxylative condensation of isatin and L-proline) was examined. After 3 h, the expected adduct **4a** was obtained in 87% yield (Table 1, entry 1). We were pleased to see that our reaction afforded the adduct **4a** with excellent diastereoselectivity (99:1, determined by ^1^H-NMR). The structure of **4a** was further confirmed by a single crystal X-ray crystallographic study (Figure 1) [14]. The ORTEP diagram of **4a** shows that: (i) the pair of linked pyrrole rings of pyrrolizidine nucleus adopts an envelope-like conformation, (ii) H-3, H-4 and H-5 are all *cis* and (iii) the two carbonyls linked to C-2 and C-3 of **4a** have a *trans* stereochemical relationship. This can be explained by the fact that the corresponding *endo* transition state (A) would require less free energy of activation than the *exo* transition state (B) leading to **4a′** as the latter would result in electrostatic repulsion between the *cis* carbonyls increasing the free energy of activation (Scheme 2). Therefore, the relative configuration of **4a** was assigned as shown in Table 1.

**Table 1:** Optimization of reaction conditions^a^. 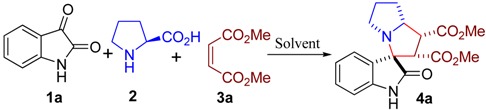

Entry	Solvent	Temp / °C	Yield / % ^b^
1	methanol	60	87
2	ethanol	60	63
3	isopropanol	60	71
4	acetonitrile	60	82
5	chloroform	60	84
6	tetrahydrofuran	60	94
7	1,4-dioxane	60	99
8	1,4-dioxane	25	37
9^c^	1,4-dioxane	reflux	94

^a^ Unless indicated otherwise, the reaction was carried out in 0.2 mmol scale in solvent (1 mL) at 60 °C for 3 h, and the ratio of **1a**/**2**/**3a** is 1:1:1. ^b^ Isolated yield based on isatin. ^c^ 45 min.

To improve the yield, efforts were made to optimize other reaction parameters including solvents and reaction temperatures. Thus, the reaction was studied in different solvents that included ethanol, isopropanol, acetonitrile, chloroform, tetrahydrofuran and 1,4-dioxane (Table 1, entries 2–7). To our satisfaction, the reaction in 1,4-dioxane led to the desired product in almost quantitative yield (99%) and maintained stereoselectivity (Table 1, entry 7), while ethanol as solvent gave the product in only 63% yield (Table 1, entry 2). In general, reactions carried out in aprotic solvents were better yielding than those in protic solvents. Temperature influenced the rate of the reaction. Elevating the reaction temperature resulted in a high reactivity and the reaction time was shortened to 45 min (Table 1, entry 9). Based on the consideration of reaction time and yield, the optimized conditions were those shown in Table 1, entry 7.

To show the general nature of the reaction, isatin bearing different substituents and L-proline were reacted with maleates (maleamide) under optimized conditions. Various functional groups appeared to be well tolerated and gave the corresponding spiropyrrolizidine oxindoles in moderate to good yields (51–99%) with excellent diastereoselectivities (up to 99:1). The results are summarized in Table 2. For dimethyl maleate, the results showed that the reaction took place with excellent diastereo-selectivities of up to 99:1, regardless of the electronic and steric nature of the substituted isatins. However, the yields of the reaction were affected by the substitutent group on the isatins (Table 2, entries 1-6). 5-Bromoisatin resulted in low to 71% yield, with an extention of the reaction time to 8 h (Table 2, entry 3). The oxindole core may also be modified. Thus, the N-protecting group may be changed as well. Incorporating different protecting groups on the N1 of oxindole had little effects on reactivity and diastereoselectivity (Table 2, entries 7–8). For the diethyl maleate, a similar phenomenon was observed. Substituents on the isatins influenced the diastereoselectivities only slightly, but affected the yields to a greater extent (Table 2, entries 9–16). Generally, isatins with electron-withdrawing groups gave lower yields than those with electron-donating groups. However, when we further expanded the substrate scope to maleamide (Table 2, entries 17–18), the corresponding products were obtained in moderate yields (51–64%). The stereochemistry of the other products was assigned by analogy to the relative configuration of **4a**.

**Table 2:** Synthesis of racemic spiropyrrolizidine oxindoles^a^. 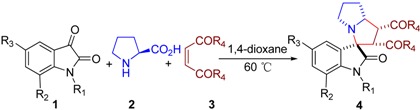

Entry		1	3	4	Time / h	Yield / % ^b^	dr ^c^
1	R_1_=R_2_=R_3_= H		R_4_=Me	**4a**	3	99	99/1
2	R_1_=R_2_=H, R_3_=CH_3_		R_4_=Me	**4b**	2	95	97/3
3	R_1_=R_2_=H, R_3_=Br		R_4_=Me	**4c**	8	71	95/5
4	R_1_=H, R_2_=R_3_=F		R_4_=Me	**4d**	3	90	99/1
5	R_1_=H, R_2_=R_3_=Cl		R_4_=Me	**4e**	3	89	99/1
6	R_1_=R_2_=H, R_3_=COOH		R_4_=Me	**4f**	3	95	99/1
7	R_1_=Et, R_2_=R_3_=H		R_4_=Me	**4g**	3	88	99/1
8	R_1_=benzyl,R_2_=R_3_=H		R_4_=Me	**4h**	2	93	99/1
9^c^	R_1_=R_2_=R_3_=H		R_4_=Et	**4i**	2	97	94/6
10	R_1_=R_2_=H, R_3_=CH_3_		R_4_=Et	**4j**	2	92	95/5
11	R_1_=R_2_=H, R=Br		R_4_=Et	**4k**	5	70	92/8
12	R_1_=H, R_2_=R_3_=F		R_4_=Et	**4l**	3	88	99/1
13	R_1_=H, R_2_=R_3_=Cl		R_4_=Et	**4m**	3	94	99/1
14	R_1_=R_2_=H, R_3_=COOH		R_4_=Et	**4n**	3	91	99/1
15	R_1_=Et, R_2_=R_3_=H		R_4_=Et	**4o**	3	92	99/1
16	R_1_=benzyl,R_2_=R_3_=H		R_4_=Et	**4p**	2	95	99/1
17	R_1_=R_2_=R_3_=H		R_4_=NH_2_	**4q**	2	64	98/2
18	R_1_=R_2_=H, R_3_=CH_3_		R_4_=NH_2_	**4r**	2	51	85/5

^a ^The reaction was carried out in 0.2 mmol scale in 1,4-dioxane (1 mL) at 60 °C, and the ratio of **1**/**2**/**3** is 1:1:1. ^b^ Isolated yield based on substituted isatins. ^c^ The dr refers to the diastereoselectivity and was determined by ^1^H-NMR.

## 3. Experimental

### 3.1. General

All chemicals were obtained from commercial sources and used without further purification. Column chromatography was carried out on silica gel (300–400 mesh, Qingdao Marine Chemical Ltd., Qingdao, China). Thin layer chromatography (TLC) was performed on TLC silica gel 60 F254 plates. ^1^H-NMR and ^13^C-NMR spectra were recorded on Varian Gemini 400 Bruker AVII-400 or Bruker AVII-600 spectrometers. The chemical shifts were recorded in ppm relative to tetramethylsilane and with the solvent resonance as the internal standard. Data are reported as follows: chemical shift, multiplicity (s = singlet, d = doublet, t = triplet, m = multiplet, br = broad), coupling constants (Hz), integration. Chemical shifts are reported in ppm from the tetramethylsilane with the solvent resonance as internal standard. Mass Spectra (MS) were measured by 3200 Q TRAP LC/MS/MS utilizing electrospray ionization (ESI).

### 3.2. Experimental Procedures

A mixture of isatin (0.2 mmol), L-proline (1 eq.), dimethyl maleate (1 eq.) in 1,4-dioxane (1 mL) was stirred for 3 h at 60 °C. After completion of the reaction (TLC), the solvent was removed under vacuum. The crude product was subjected to column chromatography on silica gel using CH_2_Cl_2_- ethyl acetate (2:1) as the eluent to give **4a** (60 mg, 87% yield). Compounds **4b-r** were synthesized by a similar procedure as described for compound **4a**. For the separation of these compounds, the eluent of silica gel column chromatography consisted of appropriate mixtures of CH_2_Cl_2_ and ethyl acetate or CH_2_Cl_2_ and MeOH.

### 3.3. Spectral Data

*Dimethyl 2-oxo-1',2',5',6',7',7a'-hexahydrospiro[indoline-3,3'-pyrrolizine]-1',2'-dicarboxylate* (**4a**). Yield 99%; White solid; m.p. 194.5–197.8 °C; ^1^H-NMR (CDCl_3_, 400 MHz): δ 1.81–1.93 (m, 2H), 1.95–2.01 (m, 2H), 2.49–2.52 (m, 1H), 3.10–3.16 (m, 1H), 3.39 (s, 3H), 3.77 (s, 3H), 3.88–3.96 (m, 2H), 4.26–4.32 (m, 1H), 6.88 (d, *J* = 7.2 Hz, 1H), 7.00–7.04 (m, 1H), 7.23–7.25 (m, 1H), 7.64 (d, *J =* 7.6 Hz, 1H), 8.26 (s, 1H); ^13^C-NMR (CDCl_3_, 100 MHz): δ 27.3, 27.7, 47.3, 47.6, 51.5, 51.7, 56.1, 66.6, 71.1, 110.1, 122.2, 125.9, 127.9, 129.4, 141.8, 170.8, 172.4, 181.4; HRMS: calcd. for C_18_H_20_N_2_O_5_^+^ [M+H]^+^: 345.1472, found: 345.1447.

*Dimethyl 5-methyl-2-oxo-1',2',5',6',7',7a'-hexahydrospiro[indoline-3,3'-pyrrolizine]-1',2'-dicarbo -xylate* (**4b**). Yield 95%; White solid; m.p. 187.2–189.6 °C; ^1^H-NMR (CDCl_3_, 600 MHz): δ 1.88–1.92 (m, 2H), 1.96–2.02(m, 2H), 2.31 (s, 3H), 2.51–2.54(m, 1H), 3.16–3.20 (m, 1H), 3.41 (s, 3H), 3.77 (s, 3H), 3.81 (d, *J =* 7.8 Hz, 1H), 4.01–4.04(m, 1H), 4.29 (q, *J =* 8.4 Hz, 1H), 6.80 (d, *J =* 7.8 Hz, 1H), 7.04 (d, *J =* 7.2 Hz, 1H), 7.37 (s, 1H), 9.33 (s, 1H). ^13^C-NMR (CDCl_3_, 150 MHz): δ 21.3, 27.3, 27.9, 47.5, 47.7, 51.5, 51.7, 55.4, 66.5, 71.2, 110.0, 12.6, 128.3, 129.8, 131.5, 139.5, 171.2, 172.2, 181.3; HRMS: calcd. for C_19_H_22_N_2_O_5_^+^ [M+H]^+^: 359.1529, found: 359.1626.

*Dimethyl 5-bromo-2-oxo-1',2',5',6',7',7a'-hexahydrospiro[indoline-3,3'-pyrrolizine]-1',2'-dicarbo -xylate* (**4c**). Yield 71%; White solid; m.p. 119.3–121.6 °C; ^1^H-NMR (CDCl_3_, 400 MHz): δ 1.71–1.78 (m, 1H), 1.85–1.92 (m, 1H), 1.94–2.00 (m, 2H), 2.47–2.50 (m, 1H), 2.96–3.02 (m, 1H), 3.43 (s, 3H), 3.78 (s, 3H), 3.82–3.86 (m, 1H), 3.93–3.95 (m, 1H), 4.21–4.27 (m, 1H), 6.79 (d, *J* = 8.0 Hz, 1H), 7.38 (d, *J* = 8.0 Hz, 1H), 7.83 (s, 1H), 7.17 (s, 1H); ^13^C-NMR (CDCl_3_, 100 MHz): δ 27.4, 27.5, 46.7, 47.3, 51.7, 51.8, 56.6, 66.7, 70.7, 111.5, 115.1, 128.3, 131.0, 132.2, 140.7, 170.2, 172.1, 181.0; HRMS: calcd. for C_18_H_19_BrN_2_O_5_^+^ [M+H]^+^: 423.0477, 425.0457, found: 423.0596, 425.0580.

*Dimethyl 5,7-difluoro-2-oxo-1',2',5',6',7',7a'-hexahydrospiro[indoline-3,3'-pyrrolizine]-1',2'-dica -rboxylate* (**4d**). Yield 90%; White solid; m.p. 193.9–195.5 °C; ^1^H-NMR (CDCl_3_, 600 MHz): δ 1.55–1.60 (m, 1H), 1.90–1.99 (m, 3H), 2.44–2.47 (m, 1H), 2.84–2.88 (m, 1H), 3.42(s, 3H), 3.68–3.71 (m, 1H), 3.78 (s, 3H), 4.09–4.10 (d, *J =* 8.4 Hz, 1H), 4.18–4.22 (m, 1H), 6.81–6.84 (m, 1H), 7.51 (q, *J =* 8.4, 1H), 8.21 (s, 1H); ^13^C-NMR (CDCl_3_, 150 MHz): δ 26.9, 27.4, 29.7, 45.6, 46.9, 51.8, 57.7, 66.7, 70.3, 104.6 (dd, *J =* 28, 21 Hz), 111.8 (dd, *J =* 25, 3 Hz), 124.9 (dd, *J =* 12, 3 Hz), 130.0 (dd, *J =* 9, 3 Hz), 146.1 (dd, *J =* 244, 11 Hz), 158.2 (dd, *J =* 243, 9 Hz), 169.6, 172.3, 179.9; HRMS: calcd. for C_18_H_18_F_2_N_2_O_5_^+^ [M+H]^+^: 381.1184, found: 381.1279.

*Dimethyl 5,7-dichloro-2-oxo-1',2',5',6',7',7a'-hexahydrospiro[indoline-3,3'-pyrrolizine]-1',2'-dica -rboxylate* (**4e**). Yield 89%; White solid; m.p. 231.0–232.3 °C; ^1^H-NMR (CDCl_3_, 400 MHz): δ 1.56–1.62 (m, 1H), 1.90–2.01 (m, 3H), 2.43–2.47 (m, 1H), 2.86 (q, *J* =7.6 Hz, 1H), 3.44 (s, 3H), 3.69–3.73 (m, 1H), 3.78 (s, 3H), 4.04 (d, *J* = 8.0 Hz, 1H), 4.15–4.20 (m, 1H), 7.27 (m, 1H),7.74 (d, *J* = 1.6 Hz, 1H), 8.17 (s, 1H); ^13^C-NMR (CDCl_3_, 100 MHz): δ 26.9, 27.3, 45.6, 46.9, 51.8, 57.6, 66.6, 70.8, 115.2, 126.8, 128.1, 128.9, 129.5, 137.8, 169.6, 172.1, 179.5; HRMS: calcd. for C_18_H_18_Cl_2_N_2_O_5_^+^ [M+H]^+^: 413.0593, 415.0563, found: 413.0677, 415.0646.

*1',2'-bis(methoxycarbonyl)-2-oxo-1',2',5',6',7',7a'-hexahydrospiro[indoline-3,3'-pyrrolizine]-5-carboxylic acid* (**4f**). Yield 95%; White solid; m.p. 250.0–253.0 °C; ^1^H-NMR (DMSO, 400 MHz): δ 1.84–1.92 (m, 4H), 2.31–2.34 (m, 1H), 3.11–3.16 (m, 1H), 3.40 (s, 3H), 3.49–3.57 (m, 1), 3.64 (d, *J* = 7.6 Hz, 1H), 3.70 (s, 3H), 4.02–4.12 (m, 1H), 6.83 (d, *J* = 8.0 Hz, 1H), 7.19 (d, *J* = 8.0 Hz, 1H), 7.33 (s, 1H), 10.43 (s, 1H), 12.30 (s, 1H); ^13^C-NMR (DMSO, 100 MHz): δ 27.1, 27.8, 41.1, 47.6, 51.7, 51.8, 54.8, 65.9, 70.4, 109.8, 125.8, 128.3, 128.5, 130.8, 142.0, 171.3, 172.0, 173.4, 179.5; HRMS: calcd. for C_19_H_2_N_2_O_7_^+^ [M+K]^+^: 427.1271, found: 427.1264.

*Dimethyl 1-ethyl-2-oxo-1',2',5',6',7',7a'-hexahydrospiro[indoline-3,3'-pyrrolizine]-1',2'-dicarbox -ylate* (**4g**). Yield 88%; White solid; m.p. 98.6–100.1 °C; ^1^H-NMR (CDCl_3_, 400 MHz): δ 1.26 (t, *J* = 7.2 Hz, 3H), 1.80–1.88 (m, 2H), 1.94–1.98 (m, 2H), 2.42–2.45 (m, 1H), 3.09–3.12 (m, 1H), 3.35 (s, 3H), 3.67–3.72 (m, 1H), 3.76 (s, 3H), 3.78–3.94 (m, 3H), 4.28 (q, *J* = 8.0 Hz, 1H), 6.84 (d, *J* = 7.6 Hz, 1H), 7.02 (t, *J* = 3.6 Hz, 1H), 7.30 (t, *J* = 8.0 Hz, 1H), 7.69 (d, *J* = 7.2 Hz, 1H); ^13^C-NMR (CDCl_3_, 100 MHz): δ 12.4, 27.3, 27.5, 34.8, 47.1, 47.5, 51.4, 51.6, 56.4, 66.6, 70.3, 108.1, 122.1, 125.7, 127.8, 129.3, 143.5, 170.8, 172.4, 178.4; HRMS: calcd. for C_20_H_24_N_2_O_5_^+^ [M+H]^+^: 373.1685, found: 373.1772.

*Dimethyl 1-benzyl-2-oxo-1',2',5',6',7',7a'-hexahydrospiro[indoline-3,3'-pyrrolizine]-1',2'-dicarbo -xylate* (**4h**). Yield 93%; White solid; m.p. 138.4–139.8 °C; ^1^H-NMR (CDCl_3_, 400 MHz): δ 1.73–1.78 (m, 1H), 1.88–2.01 (m, 3H), 2.44–2.47 (m, 1H), 3.05–3.10 (m, 1H), 3.26 (s, 3H), 3.78 (s, 3H), 3.83 (t, *J* = 8.0 Hz, 1H), 4.05 (d, *J* = 8.0 Hz, 1H), 4.30 (q, *J* = 7.2 Hz, 1H) , 4.76 (d, *J* = 15.6 Hz, 1H), 5.07 (d, *J* = 16.0 Hz, 1H), 6.67 (d, *J* = 7.6 Hz, 1H), 6.99 (t, *J* = 7.6 Hz, 1H), 7.16 (t, *J* = 7.6 Hz, 1H), 7.23–7.32 (m, 5H), 7.77 (d, *J* = 7.6 Hz, 1H); ^13^C-NMR (CDCl_3_, 100 MHz): δ 27.1, 27.4, 43.8, 46.8, 47.4, 51.4, 51.6, 57.3, 66.8, 70.2, 109.1, 122.4, 125.8, 127.1, 127.6, 127.7, 128.7, 129.2, 135.8, 143.4, 170.5, 172.5, 179.3; HRMS: calcd. for C_25_H_26_N_2_O_5_^+^ [M+H]^+^: 435.1842, found: 435.1880.

*Diethyl 2-oxo-1',2',5',6',7',7a'-hexahydrospiro[indoline-3,3'-pyrrolizine]-1',2'-dicarboxylate* (**4i**). Yield 97%; White solid; m.p. 180.4–183.0 °C; ^1^H-NMR (CDCl_3_, 400 MHz): δ 0.85 (t, *J* = 7.2 Hz, 3H), 1.32 (t, *J* = 7.2 Hz, 3H), 1.81–1.91 (m, 2H), 1.93–2.00 (m, 2H), 2.46–2.50 (m, 1H), 3.03–3.07 (m, 1H), 3.82–3.87 (m, 3H), 3.93–3.96 (m, 1H), 4.21–4.28 (m, 3H), 6.89 (d, *J* = 7.6 Hz, 1H), 7.00 (t, *J* = 7.6 Hz, 1H), 7.21–7.27 (m, 1H), 7.72 (d, *J* = 7.6 Hz, 1H), 8.91 (s, 1H);^13^C-NMR (CDCl_3_, 100 MHz): δ 13.6, 14.3, 27.3, 27.5, 47.1, 47.5, 56.5, 60.6, 66.7, 71.1, 110.0, 122.2, 126.2, 128.2, 129.3, 141.8, 170.1, 171.9, 181.6; HRMS: calcd. for C_20_H_24_N_2_O_5_^+^ [M+H]^+^: 373.1685, found: 373.1766.

*Diethyl 5-methyl-2-oxo-1',2',5',6',7',7a'-hexahydrospiro[indoline-3,3'-pyrrolizine]-1',2'-dicarbox -ylate* (**4j**). Yield 92%; White solid; m.p. 155.0–157.3 °C; ^1^H-NMR (CDCl_3_, 600 MHz): δ 0.88 (t, *J =* 7.2 Hz, 3H), 1.31 (t, *J =* 7.2 Hz, 3H), 1.86–1.91 (m, 2H), 1.97–2.02 (m, 2H), 2.30 (s, 3H), 2.50–2.52 (m, 1H), 3.11–3.15 (m, 1H), 3.83–3.95 (m, 4H), 4.22–4.28 (m, 3H), 6.79 (d, *J =* 7.8 Hz, 1H), 7.04 (d, *J =* 8.4 Hz, 1H), 7.45 (s, 1H), 9.29 (s, 1H). ^13^C-NMR (CDCl_3_, 150 MHz): δ 13.6, 14.3, 21.3, 27.3, 27.7, 47.4, 47.6, 55.9, 60.51, 60.52, 66.6, 71.2, 109.9, 125.9, 128.6, 129.6, 131.4, 139.5, 170.5, 171.8, 181.6; HRMS: calcd. for C_21_H_26_N_2_O_5_^+^ [M+H]^+^: 387.1842, found: 387.1908.

*Diethyl 5-bromo-2-oxo-1',2',5',6',7',7a'-hexahydrospiro[indoline-3,3'-pyrrolizine]-1',2'-dicarbox -ylate* (**4k**). Yield 70%; White solid; m.p. 174.3–176.5 °C; ^1^H-NMR (CDCl_3_, 400 MHz): δ 0.89 (t, *J* = 6.8 Hz, 3H), 1.31 (t, *J* = 7.2 Hz, 3H), 1.73–1.76 (m, 1H), 1.83–1.90 (m, 1H), 1.94–1.99 (m, 2H), 2.45–2.48 (m, 1H), 2.92–2.98 (m, 1H), 3.74–3.78 (m, 1H), 3.83–3.88 (m, 1H), 3.92–3.96 (m, 2H), 4.20–4.27 (m, 3H), 6.78 (d, *J* = 8.4 Hz, 1H), 7.35–7.37 (m, 1H), 7.89 (d, *J* = 1.6 Hz, 1H), 9.17 (s, 1H); ^13^C-NMR (CDCl_3_, 100 MHz): δ 13.6, 14.3, 27.3, 27.4, 46.6, 47.3, 56.9, 60.7, 60.8, 66.7, 70.8, 111.4, 115.1, 128.6, 131.2, 132.1, 140.8, 169.6, 171.6, 181.3; HRMS: calcd. for C_20_H_23_BrN_2_O_5_^+^ [M+H]^+^: 451.0790, 453.0770, found: 451.0746, 453.0753.

*Diethyl 5,7-difluoro-2-oxo-1',2',5',6',7',7a'-hexahydrospiro[indoline-3,3'-pyrrolizine]-1',2'-dicarb -oxylate* (**4l**). Yield 88%; White solid; m.p. 119.3–121.8 °C; ^1^H-NMR (CDCl_3_, 600 MHz): δ 0.90 (t, *J =* 7.2 Hz, 3H), 1.33 (t, *J =* 6.6 Hz, 3H), 1.54–1.56 (m, 1H), 1.90–1.94 (m, 2H), 1.95–2.00 (m, 1H), 2.44–2.46 (m, 1H), 2.80–2.84 (m, 1H), 3.60–3.63 (m, 1H), 3.84–3.92 (m, 2H), 4.10 (d, *J =* 8.4 Hz, 1H), 4.17–4.20 (m, 1H), 4.23–4.26 (m, 2H), 6.80–6.84 (m, 1H), 7.60 (d, *J =* 8.4 Hz, 1H), 7.66 (s, 1H); ^13^C-NMR (CDCl_3_, 150 MHz): δ 13.6, 14.4, 26.8, 27.4, 45.4, 46.9, 58.2, 60.7, 60.8, 66.8, 70.2, 104.4 (dd, *J =* 28, 22 Hz), 112.2 (dd, *J =* 26, 3 Hz), 124.9 (dd, *J =* 12, 3 Hz), 130.5 (dd, *J =* 9, 3 Hz), 146.1 (dd, *J =* 243, 13 Hz), 158.3 (dd, *J =* 242, 10 Hz), 169.0, 171.8, 179.8; HRMS: calcd. for C_20_H_22_F_2_N_2_O_5_^+^ [M+H]^+^: 409.1497, found: 409.1551.

*Diethyl 5,7-dichloro-2-oxo-1',2',5',6',7',7a'-hexahydrospiro[indoline-3,3'-pyrrolizine]-1',2'-dicarb -oxylate* (**4m**). Yield 94%; White solid; m.p. 197.6–199.1 °C; ^1^H-NMR (CDCl_3_, 600 MHz): δ 0.90 (t, *J* = 7.2 Hz, 3H), 1.33 (t, *J* = 7.2 Hz, 3H), 1.60–1.63 (m, 1H), 1.90–2.00 (m, 3H), 2.45–2.47 (m, 1H), 2.86 (m, 1H), 3.66 (t, *J* = 7.8 Hz, 1H), 3.84–3.87 (m, 1H), 3.92–3.95 (m, 1H), 4.07 (t, *J* = 12.0 Hz, 1H), 4.17–4.21 (m, 1H), 4.23–4.27 (m, 2H), 7.27–7.29 (m, 1H),7.82 (s, 1H), 8.79 (s, 1H); ^13^C-NMR (CDCl_3_, 150 MHz): δ 13.6, 14.3, 26.8, 27.4, 45.6, 46.9, 57.9, 60.7, 60.8, 66.7, 70.9, 115.3, 127.0, 128.0, 128.7, 129.7, 138.1, 169.1, 171.6, 180.3; HRMS: calcd. for C_20_H_22_Cl_2_N_2_O_5_^+^ [M+H]^+^: 441.0906, 443.0876, found: 441.0980, 443.0964.

*1',2'-bis(ethoxycarbonyl)-2-oxo-1',2',5',6',7',7a'-hexahydrospiro[indoline-3,3'-pyrrolizine]-5-carboxylic acid* (**4n**). Yield 91%; White solid; m.p. 228.1–230.6 °C;^1^H-NMR (DMSO, 600 MHz): δ0.80 (t, *J* =7.2 Hz, 3H), 1.21 (t, *J* = 7.2 Hz, 3H), 1.71–1.76 (m, 1H), 1.84–1.89 (m, 2H), 2.23–2.26 (m, 1H), 3.01 (q, *J* = 7.2 Hz,1H), 3.42–3.48 (m, 1H), 3.63 (d, *J* = 8.4 Hz, 1H), 3.76–3.85 (m, 3H), 4.00–4.02 (m, 1H), 4.11–4.14 (m, 2H), 6.76 (d, *J* = 7.8 Hz,1H), 7.13 (d, *J* = 7.8 Hz,1H), 7.38 (s, 1H), 10.38 (s, 1H), 12.23 (brs, 1H); ^13^C-NMR (DMSO, 100 MHz): δ 13.9, 14.6, 27.2, 27.4, 47.2, 47.5, 55.6, 60.3, 60.4, 66.1, 70.4, 109.7, 126.2, 128.1, 128.8, 130.6, 142.1, 170.4, 171.6, 173.2, 179.9; HRMS: calcd. for C_21_H_24_N_2_O_7_^+^ [M+K]^+^: 455.1584, found: 455.1568.

*Diethyl 1-ethyl-2-oxo-1',2',5',6',7',7a'-hexahydrospiro[indoline-3,3'-pyrrolizine]-1',2'-dicarboxyl -ate* (**4o**). Yield 92%; White solid; m.p. 66.1–68.4 °C; ^1^H-NMR (CDCl_3_, 600 MHz): δ 0.81 (t, *J =* 7.2 Hz, 3H), 1.27 (t, *J =* 7.2 Hz, 3H), 1.32 (t, *J* = 7.2 Hz, 3H), 1.75–1.82 (m, 1H), 1.85–1.90 (m, 1H), 1.95–1.97 (m, 2H), 2.40–2.43 (m, 1H), 3.05 (q, *J* = 6.0 Hz, 1H), 3.69–3.72 (m, 1H), 3.78–3.84 (m, 4H), 3.94 (d, *J* = 8.4 Hz, 1H), 4.22–4.27 (m, 3H), 6.84 (d, *J* = 7.8 Hz, 1H), 7.00–7.03 (m, 1H), 7.28–7.30 (m, 1H), 7.80 (d, *J* = 7.2 Hz, 1H); ^13^C-NMR (CDCl_3_, 150 MHz): δ 12.5, 13.5, 14.3, 27.2, 27.3, 34.8, 46.8, 47.5, 56.9, 60.4, 60.5, 66.7, 70.2, 108.0, 122.0, 126.1, 128.0, 129.2, 143.5, 170.1, 172.0, 178.7; HRMS: calcd. for C_22_H_28_N_2_O_5_^+^ [M+H]^+^: 401.1998, found: 401.2046.

*Diethyl 1-benzyl-2-oxo-1',2',5',6',7',7a'-hexahydrospiro[indoline-3,3'-pyrrolizine]-1',2'-dicarboxy -late* (**4p**). Yield 95%; White solid; m.p. 97.2–99.3 °C; ^1^H-NMR (CDCl_3_, 400 MHz): δ 0.66 (t, *J =* 7.2 Hz, 3H), 1.26–1.35 (m, 3H), 1.71–1.78 (m, 1H), 1.95–1.98 (m, 3H), 2.41–2.46 (m, 1H), 3.02 (q, *J =* 8.0 Hz, 1H), 3.71–3.78 (m, 3H), 4.10 (d, *J* = 8.0 Hz, 1H), 4.24–4.29 (m, 3H), 4.78 (d, *J* = 16.0 Hz, 1H), 5.05 (d, *J* = 15.6 Hz, 1H), 6.67 (d, *J* = 8.0 Hz, 1H), 6.97–7.00 (m, 1H), 7.13–7.17 (m, 1H), 7.26–7.31 (m, 5H), 7.85 (d, *J* = 7.2 Hz, 1H); ^13^C-NMR (CDCl_3_, 100 MHz): δ13.4, 14.3, 26.9, 27.4, 29.7,43.9, 46.5, 47.4, 57.6, 60.4, 60.5, 66.8, 70.1, 108.9, 122.4, 126.2, 127.2, 127.5, 127.9, 128.7, 129.0, 135.8, 143.5, 169.8, 172.0, 179.5; HRMS: calcd. for C_27_H_30_N_2_O_5_^+^ [M+H]^+^: 463.2155, found: 463.2198.

*2-oxo-1',2',5',6',7',7a'-hexahydrospiro[indoline-3,3'-pyrrolizine]-1',2'-dicarboxamide* (**4q**). Yield 64%; White solid; m.p. 107.6–110.1 °C; ^1^H-NMR (DMSO, 600 MHz): δ 1.54–1.58 (m, 1H), 1.79–1.83 (m, 2H), 2.18–2.21 (m, 1H), 2.27–2.29 (m, 1H), 3.13–3.17 (m, 2H), 3.35–3.39 (m, 1H), 4.02–4.04 (m, 2H), 6.61 (s, 1H), 6.79–6.91 (m, 4H), 7.03(s, 1H), 7.51 (d, *J =* 8.4 Hz, 1H), 10.31 (s, 1H); ^13^C-NMR (DMSO, 150 MHz): δ 27.4, 28.0, 48.8, 49.0, 53.7, 65.9, 71.1, 109.7, 121.2, 125.7, 129.2, 129.4, 143.5, 172.7, 172.8, 180.1; HRMS: calcd. for C_16_H_18_N_4_O_3_^+^ [M+H]^+^: 315.1379, found: 315.1443.

*5-methyl-2-oxo-1',2',5',6',7',7a'-hexahydrospiro[indoline-3,3'-pyrrolizine]-1',2'-dicarboxamide* (**4r**). Yield 51%; White solid; m.p. 244.9–247.1 °C; ^1^H-NMR (DMSO, 400 MHz): δ 1.54–1.61 (m, 2H), 1.78–1.84 (m, 3H), 2.22 (s, 3H), 3.17 (d, *J =* 4.4 Hz, 2H), 3.99 (d, *J =* 4.0 Hz, 2H), 6.60 (s, 1H), 6.67 (d, *J =* 7.6 Hz, 1 H), 6.82 (d, *J =* 7.6 Hz, 2 H), 7.01(d, *J =* 8.0 Hz, 2H), 7.36 (s, 1H), 10.15(s, 1H); ^13^C-NMR (DMSO, 100 MHz): δ 21.5, 27.4, 27.9, 48.7, 49.1, 54.1, 65.9, 71.1, 109.3, 125.8, 129.7 129.9, 141.0, 172.7, 172.8, 180.3; HRMS: calcd. for C_17_H_20_N_4_O_3_^+^ [M+H]^+^: 329.1535, found: 329.1547.

## 4. Conclusions

In this work, we have developed an efficient method for the synthesis of potentially biologically active spiropyrrolizidine oxindoles via a three-component 1,3-dipolar cycloaddition reaction. A range of spiropyrrolizidine oxindoles bearing two ester or two amide groups were obtained in high yields (up to 99%) with excellent diastereoselectivities (up to 99:1 dr). The methodology is rapid, simple, and inexpensive affording complex compounds. Further study on the antibacterial, antiviral and antitumor activities of these compounds is underway. Supporting Information for this article is available online at http://www.mdpi.com/journal/molecules/. Included crystallographic data and molecular structure, ^1^H-, ^13^C-NMR and HRMS spectra of all compounds.

## Supplementary Materials

Supplementary materials can be accessed at http://www.mdpi.com/1420-3049/16/10/8745/s1.
