# Supplementary material for: Facile Synthesis of Functionalized Spiropyrrolizidine Oxindoles via a Three-Component Tandem Cycloaddition Reaction
Source: Molecules. 2011 Oct 19;16(10):8745–57. doi: 10.3390/molecules16108745 (PMC6264231; doi:10.3390/molecules16108745)
Supplement: Supplementary file 1 [file molecules-16-08745-s001.pdf]

Supplementary Material

Facile Synthesis of Functionalized Spiropyrrolizidine Oxindoles via a Three-Component Tandem Cycloaddition Reaction

Yong-Mei Xie [1], Yu-Qin Yao [1], Hong-Bao Sun [1], Ting-Ting Yan [1], Jie Liu [1,*] and Tai-Ran Kang [2,*]

[1] State Key Laboratory of Biotherapy, West China Hospital, West China Medical School, Sichuan University, Chengdu 610041, China; E-Mails: xieym@scu.edu.cn (Y.-M.X.); yuqin_yao@163.com (Y.-Q.Y.); 773770248@qq.com (H.-B.S.); 121968698@qq.com (T.-T.Y.)

[2] Chemical Synthesis and Pollution Control Key Laboratory of Sichuan Province, College of Chemistry and Chemical Engineering, China West Normal University, Nanchong 637002, China

* Author to whom correspondence should be addressed; E-Mails: liujie2011@scu.edu.cn (J.L.); kangtairan@cwnu.edu.cn (T.-R.K.); Tel.: +86-28-85503817; Fax: +86-28-85503817.

1. Crystallographic data and molecular structure……………………..…2
2. ^{1}H, ^{13}C-NMR Spectra……………………………………………………………..……..…...3-29

1. Crystallographic Data and Molecular Structure

Crystal structure data for compound **4a**: CCDC 828257.

$C_{18}H_{20}N_2O_5$, chemical formula weight: 344.14, monoclinic, space group $P2_1/c$, a = 7.5912 (6), b = 27.380 (2), c = 7.8522 (6) Å; α = 90.00°, β = 95.729 (8) °, γ = 90.00°, U = 1623.9 (2) $Å^3$, T = 145.0 K, Z = 4, ρ = 1.413 mg/mm^3, μ = 0.104 mm^{-1}, F(000)732, crystal size 0.30 × 0.25 × 0.20 mm^3, 5840 independent reflections [R(int) = 0.0000], reflections collected 5840, refinement method: full-matrix least-squares on F^2: Goodness-of-fit on F^2 0.721, final R indexes [$I > 2\sigma(I)$], R_1 = 0.0468, wR_2 = 0.0683, largest diff. peak and hole 0.243 $Å^{-3}$ and −0.246 $eÅ^{-3}$.

Crystal Structure of **4a**.

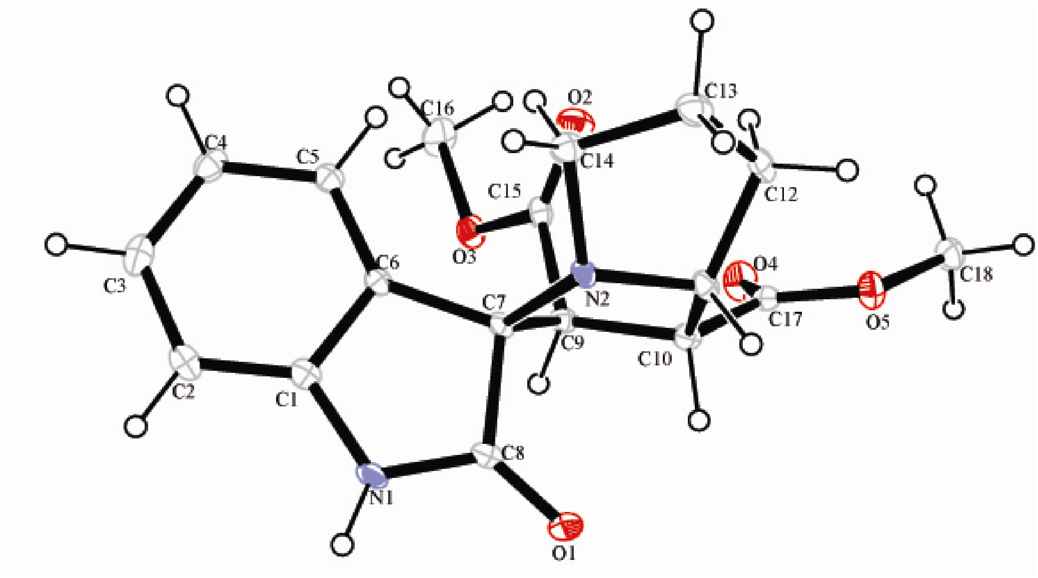

2. ^{1}H, ^{13}C NMR Spectra

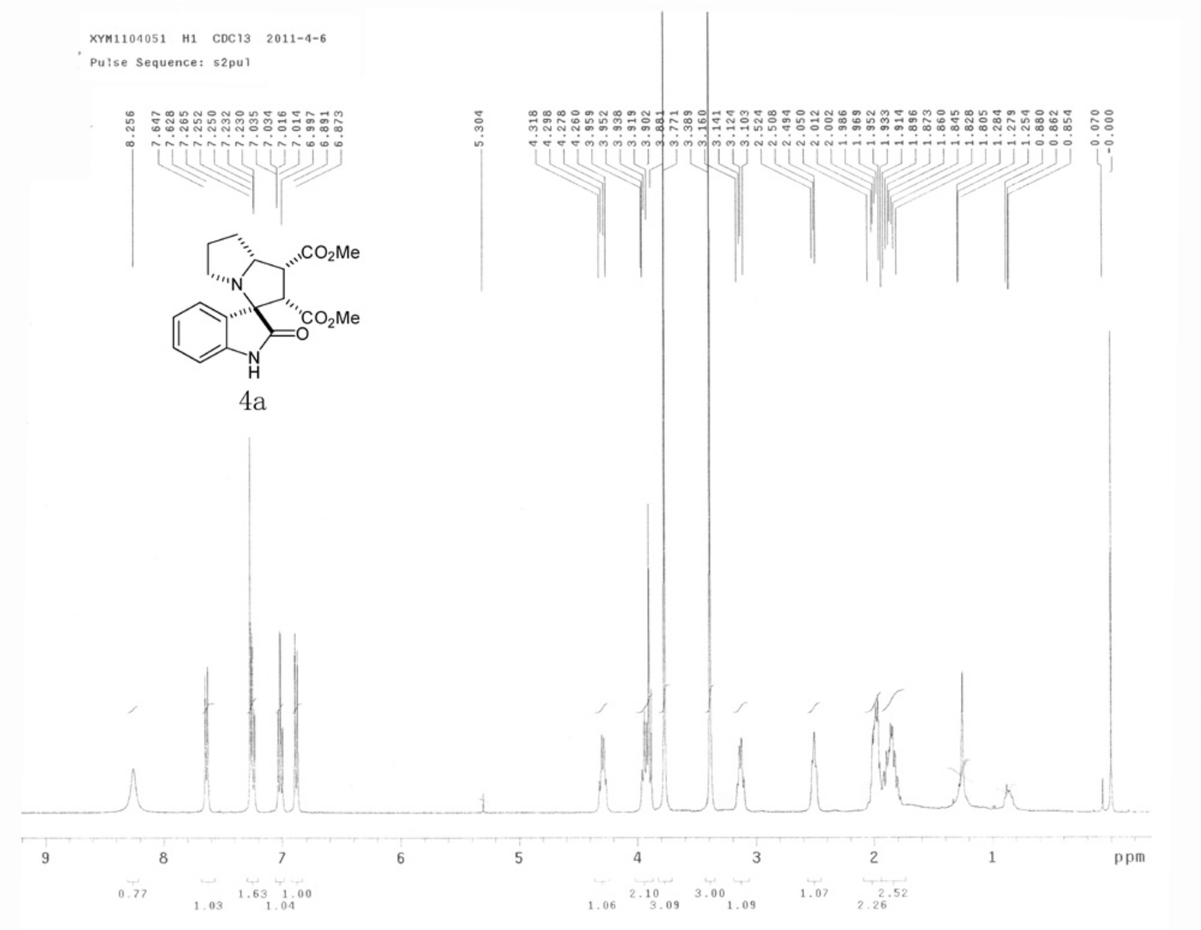

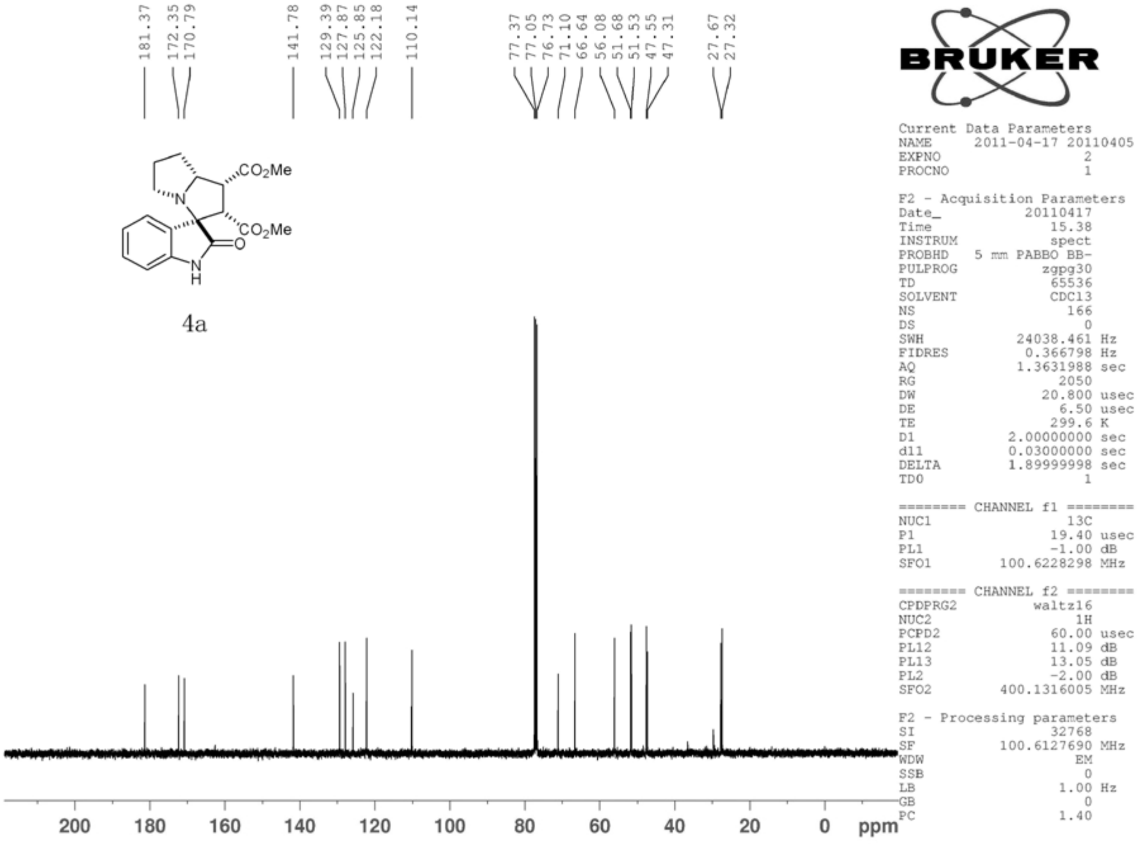

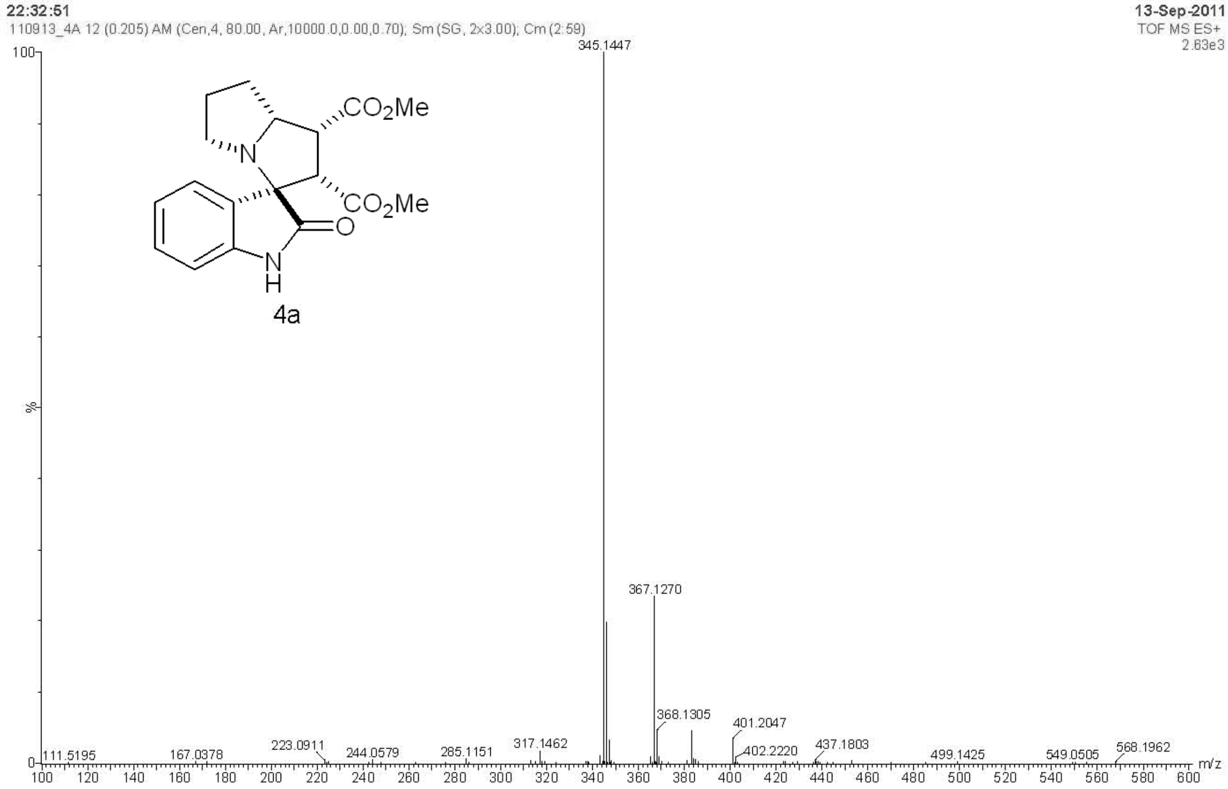
22:32:51
110913_4A 12 (0.205) AM (Cen,4, 80.00, Ar,10000.0,0.00,0.70); Sm (SG, 2x3.00); Cm (2:59)
13-Sep-2011
TOF MS ES+
2.63e3
CO2Me
CO2Me
N
O
N
H
4a
%
345.1447
367.1270
368.1305
401.2047
402.2220
437.1803
111.5195
167.0378
223.0911
244.0579
285.1151
317.1462
499.1425
549.0505
568.1962
m/z

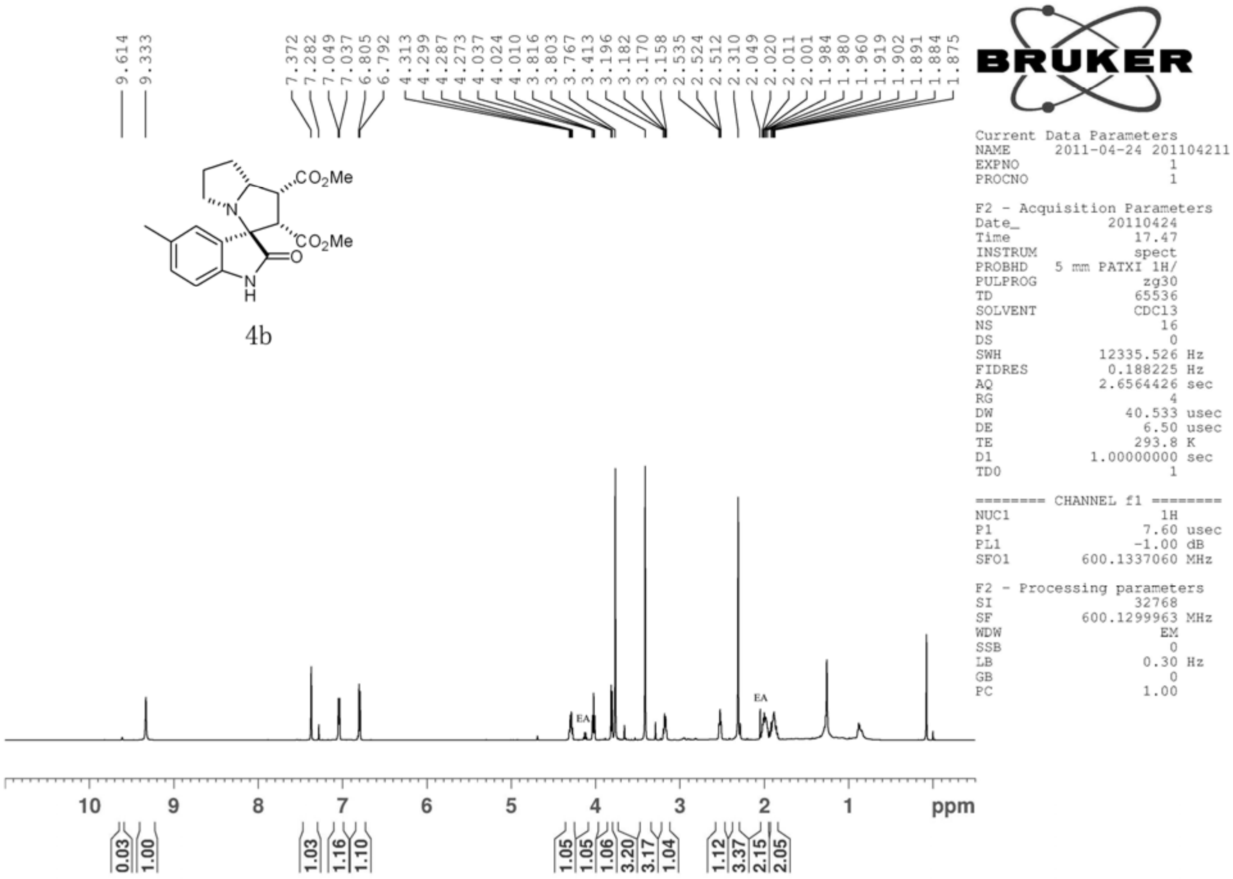
9.614
9.333
7.372
7.282
7.049
7.037
6.805
6.792
4.313
4.299
4.287
4.273
4.037
4.024
4.010
3.816
3.803
3.767
3.413
3.196
3.182
3.170
3.158
2.535
2.524
2.512
2.310
2.049
2.020
2.011
2.001
1.984
1.980
1.960
1.919
1.902
1.891
1.884
1.875
BRUKER
CO2Me
CO2Me
N
O
N
H
4b
Current Data Parameters
NAME 2011-04-24 201104211
EXPNO 1
PROCNO 1
F2 - Acquisition Parameters
Date_ 20110424
Time 17.47
INSTRUM spect
PROBHD 5 mm PATXI 1H/
PULPROG zg30
TD 65536
SOLVENT CDCl3
NS 16
DS 0
SWH 12335.526 Hz
FIDRES 0.188225 Hz
AQ 2.6564426 sec
RG 4
DW 40.533 usec
DE 6.50 usec
TE 293.8 K
D1 1.00000000 sec
TD0 1
======== CHANNEL f1 ========
NUC1 1H
P1 7.60 usec
PL1 -1.00 dB
SFO1 600.1337060 MHz
F2 - Processing parameters
SI 32768
SF 600.1299963 MHz
WDW EM
SSB 0
LB 0.30 Hz
GB 0
PC 1.00
EA
EA
ppm
0.03
1.00
1.03
1.16
1.10
1.05
1.05
1.06
3.20
3.17
1.04
1.12
3.37
2.15
2.05

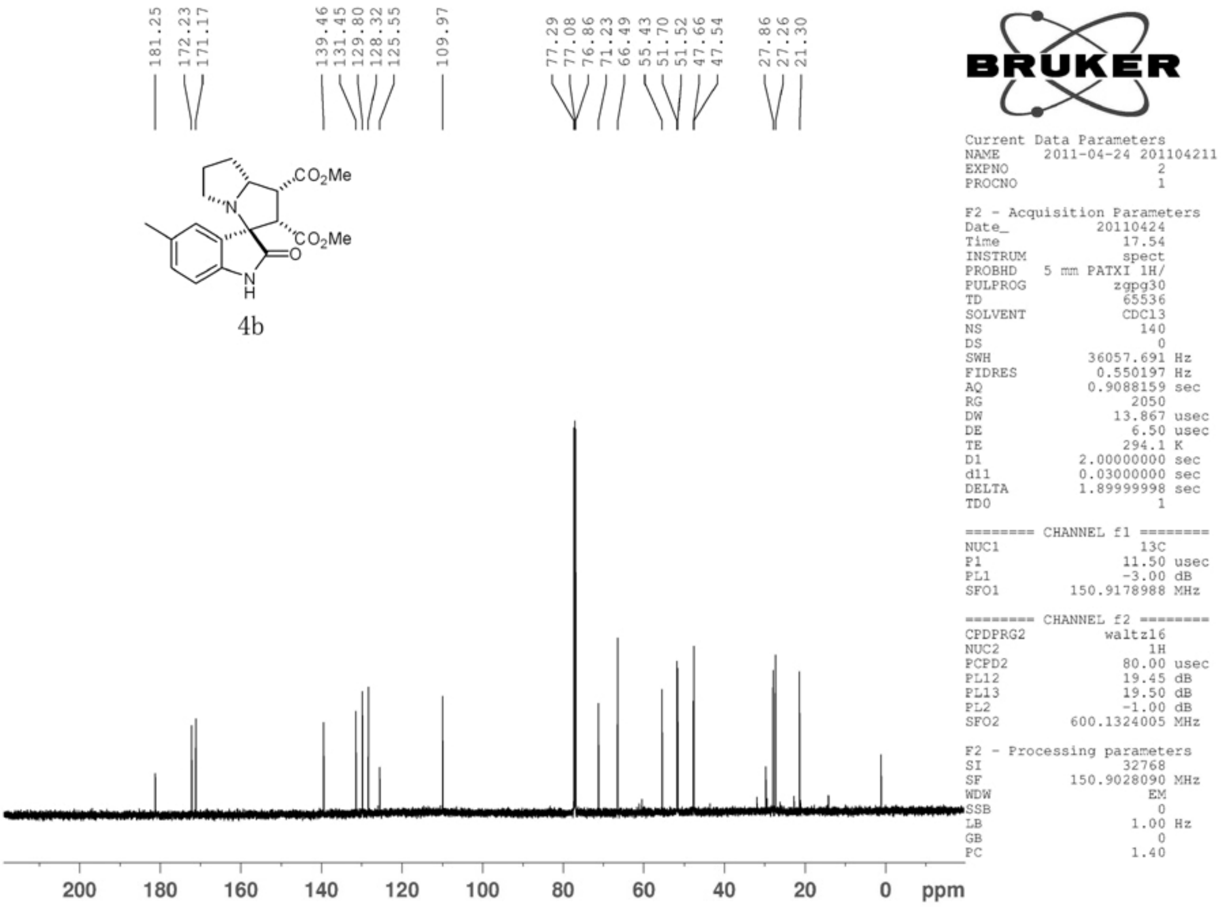
181.25
172.23
171.17
139.46
131.45
129.80
128.32
125.55
109.97
77.29
77.08
76.86
71.23
66.49
55.43
51.70
51.52
47.66
47.54
27.86
27.26
21.30
CO2Me
CO2Me
N
O
N
H
4b
BRUKER
Current Data Parameters
NAME 2011-04-24 201104211
EXPNO 2
PROCNO 1
F2 - Acquisition Parameters
Date_ 20110424
Time 17.54
INSTRUM spect
PROBHD 5 mm PATXI 1H/
PULPROG zgpg30
TD 65536
SOLVENT CDCl3
NS 140
DS 0
SWH 36057.691 Hz
FIDRES 0.550197 Hz
AQ 0.9088159 sec
RG 2050
DW 13.867 usec
DE 6.50 usec
TE 294.1 K
D1 2.00000000 sec
d11 0.03000000 sec
DELTA 1.89999998 sec
TD0 1
======== CHANNEL f1 ========
NUC1 13C
P1 11.50 usec
PL1 -3.00 dB
SFO1 150.9178988 MHz
======== CHANNEL f2 ========
CPDPRG2 waltz16
NUC2 1H
PCPD2 80.00 usec
PL12 19.45 dB
PL13 19.50 dB
PL2 -1.00 dB
SFO2 600.1324005 MHz
F2 - Processing parameters
SI 32768
SF 150.9028090 MHz
WDW EM
SSB 0
LB 1.00 Hz
GB 0
PC 1.40
200 180 160 140 120 100 80 60 40 20 0 ppm

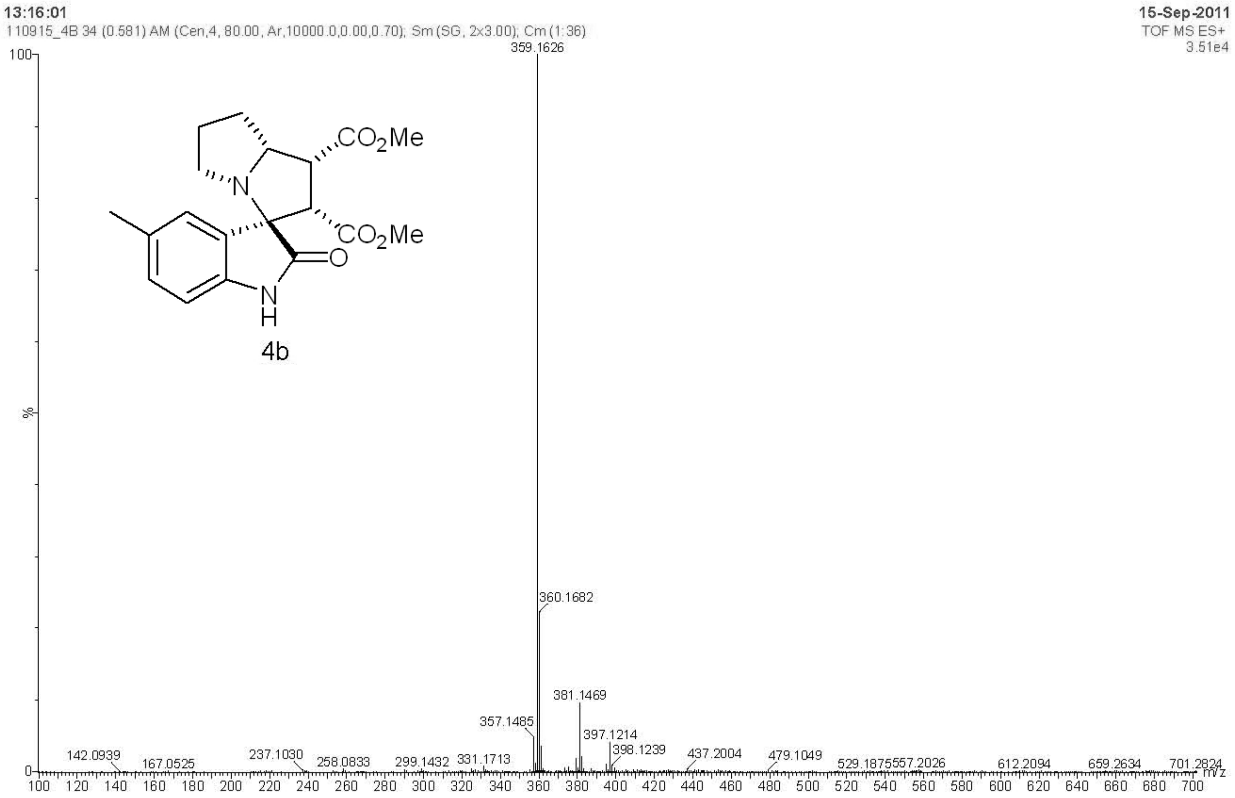
13:16:01
110915_4B 34 (0.581) AM (Cen,4, 80.00, Ar,10000.0,0.00,0.70); Sm (SG, 2x3.00); Cm (1:36)
15-Sep-2011
TOF MS ES+
3.51e4
100
%
0
359.1626
360.1682
381.1469
357.1485
397.1214
398.1239
437.2004
479.1049
529.1875
557.2026
612.2094
659.2634
701.2824
142.0939
167.0525
237.1030
258.0833
299.1432
331.1713
CO2Me
CO2Me
N
O
N
H
4b
100 120 140 160 180 200 220 240 260 280 300 320 340 360 380 400 420 440 460 480 500 520 540 560 580 600 620 640 660 680 700 m/z

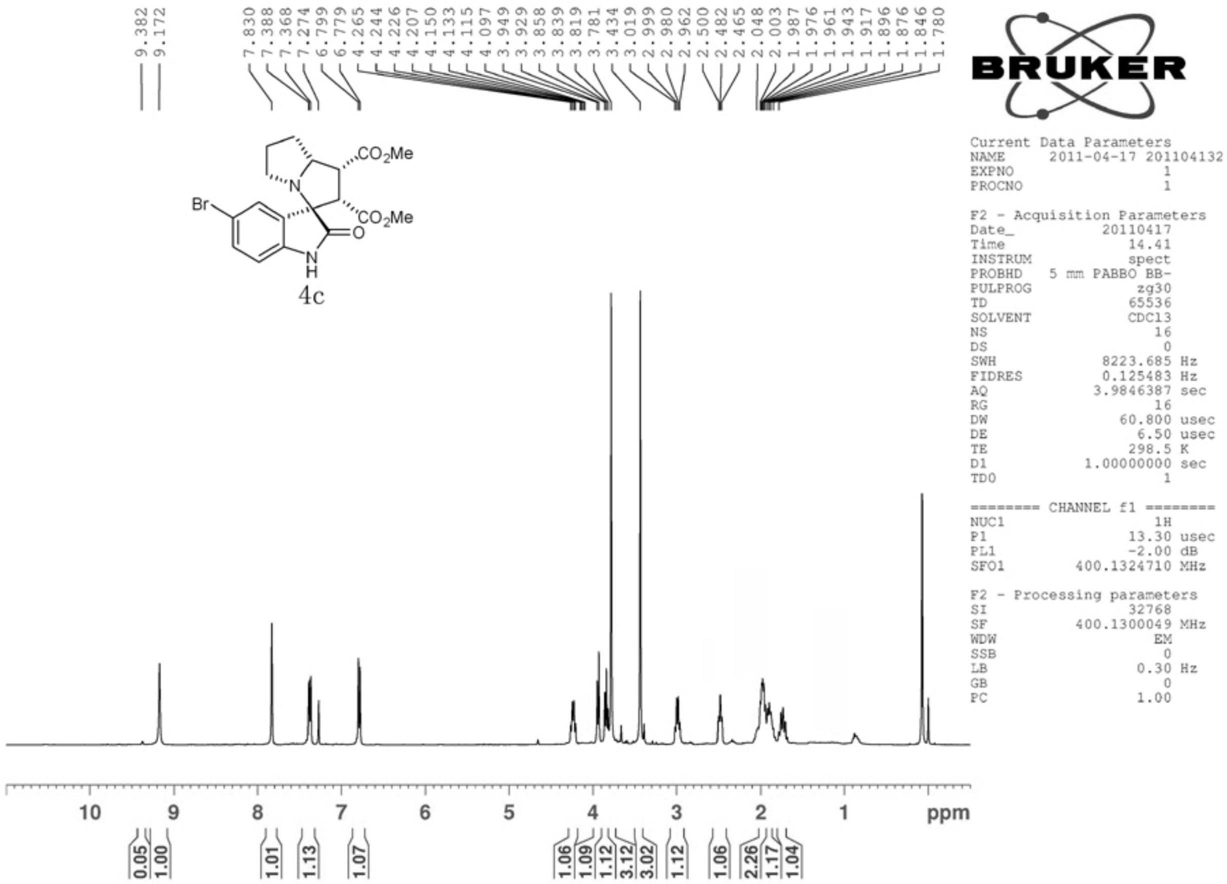
9.382
9.172
7.830
7.388
7.368
7.274
6.799
6.779
4.265
4.244
4.226
4.207
4.150
4.133
4.115
4.097
3.949
3.929
3.858
3.839
3.819
3.781
3.434
3.019
2.999
2.980
2.962
2.500
2.482
2.465
2.048
2.003
1.987
1.976
1.961
1.943
1.917
1.896
1.876
1.846
1.780
CO2Me
CO2Me
Br
N
O
N
H
4c
BRUKER
Current Data Parameters
NAME 2011-04-17 201104132
EXPNO 1
PROCNO 1
F2 - Acquisition Parameters
Date_ 20110417
Time 14.41
INSTRUM spect
PROBHD 5 mm PABBO BB-
PULPROG zg30
TD 65536
SOLVENT CDCl3
NS 16
DS 0
SWH 8223.685 Hz
FIDRES 0.125483 Hz
AQ 3.9846387 sec
RG 16
DW 60.800 usec
DE 6.50 usec
TE 298.5 K
D1 1.00000000 sec
TD0 1
======== CHANNEL f1 ========
NUC1 1H
P1 13.30 usec
PL1 -2.00 dB
SFO1 400.1324710 MHz
F2 - Processing parameters
SI 32768
SF 400.1300049 MHz
WDW EM
SSB 0
LB 0.30 Hz
GB 0
PC 1.00
10
9
8
7
6
5
4
3
2
1
ppm
0.05
1.00
1.01
1.13
1.07
1.06
1.09
1.12
3.12
3.02
1.12
1.06
2.26
1.17
1.04

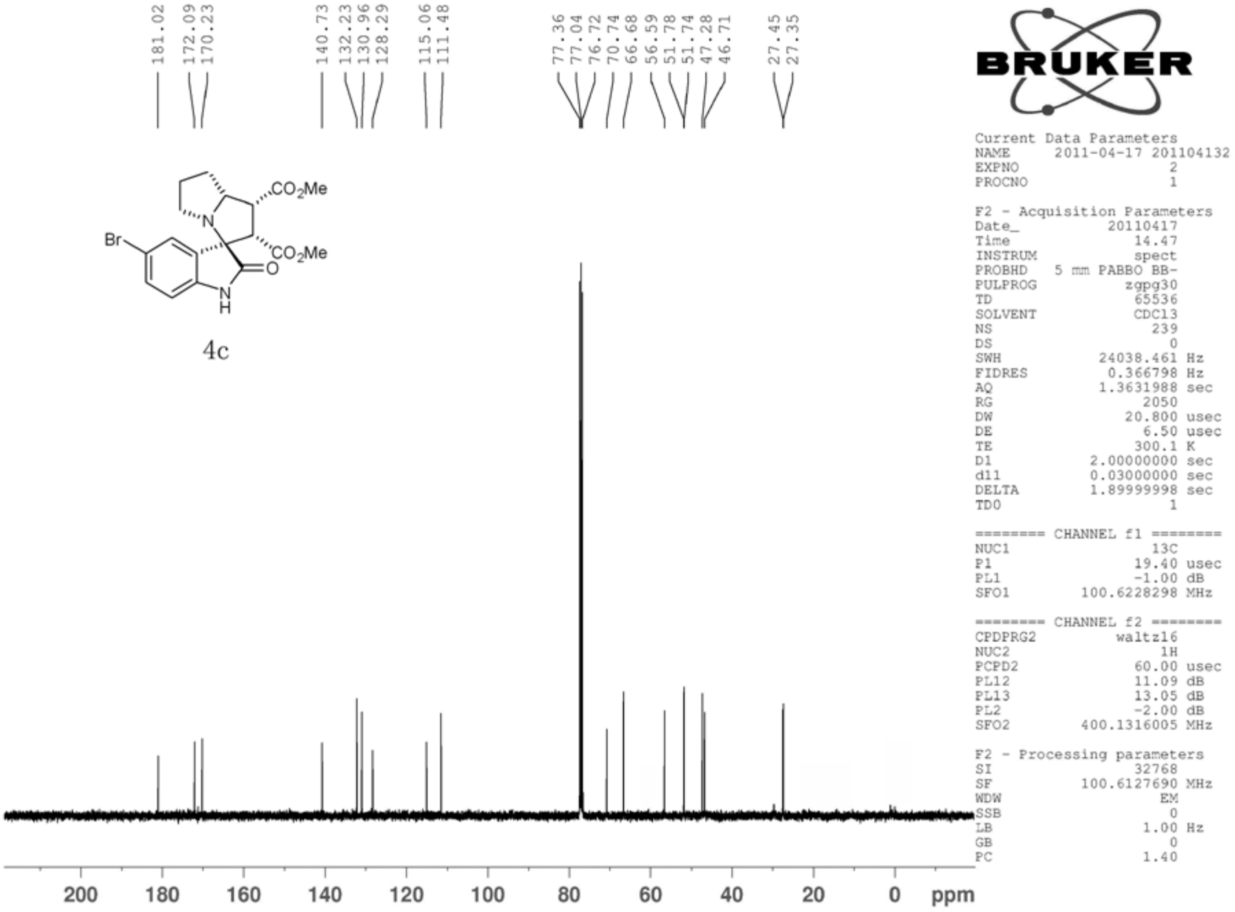
181.02
172.09
170.23
140.73
132.23
130.96
128.29
115.06
111.48
77.36
77.04
76.72
70.74
66.68
56.59
51.78
51.74
47.28
46.71
27.45
27.35
CO2Me
CO2Me
Br
N
O
N
H
4c
BRUKER
Current Data Parameters
NAME 2011-04-17 201104132
EXPNO 2
PROCNO 1
F2 - Acquisition Parameters
Date_ 20110417
Time 14.47
INSTRUM spect
PROBHD 5 mm PABBO BB-
PULPROG zgpg30
TD 65536
SOLVENT CDCl3
NS 239
DS 0
SWH 24038.461 Hz
FIDRES 0.366798 Hz
AQ 1.3631988 sec
RG 2050
DW 20.800 usec
DE 6.50 usec
TE 300.1 K
D1 2.00000000 sec
d11 0.03000000 sec
DELTA 1.89999998 sec
TD0 1
======== CHANNEL f1 ========
NUC1 13C
P1 19.40 usec
PL1 -1.00 dB
SFO1 100.6228298 MHz
======== CHANNEL f2 ========
CPDPRG2 waltz16
NUC2 1H
PCPD2 60.00 usec
PL12 11.09 dB
PL13 13.05 dB
PL2 -2.00 dB
SFO2 400.1316005 MHz
F2 - Processing parameters
SI 32768
SF 100.6127690 MHz
WDW EM
SSB 0
LB 1.00 Hz
GB 0
PC 1.40
200
180
160
140
120
100
80
60
40
20
0
ppm

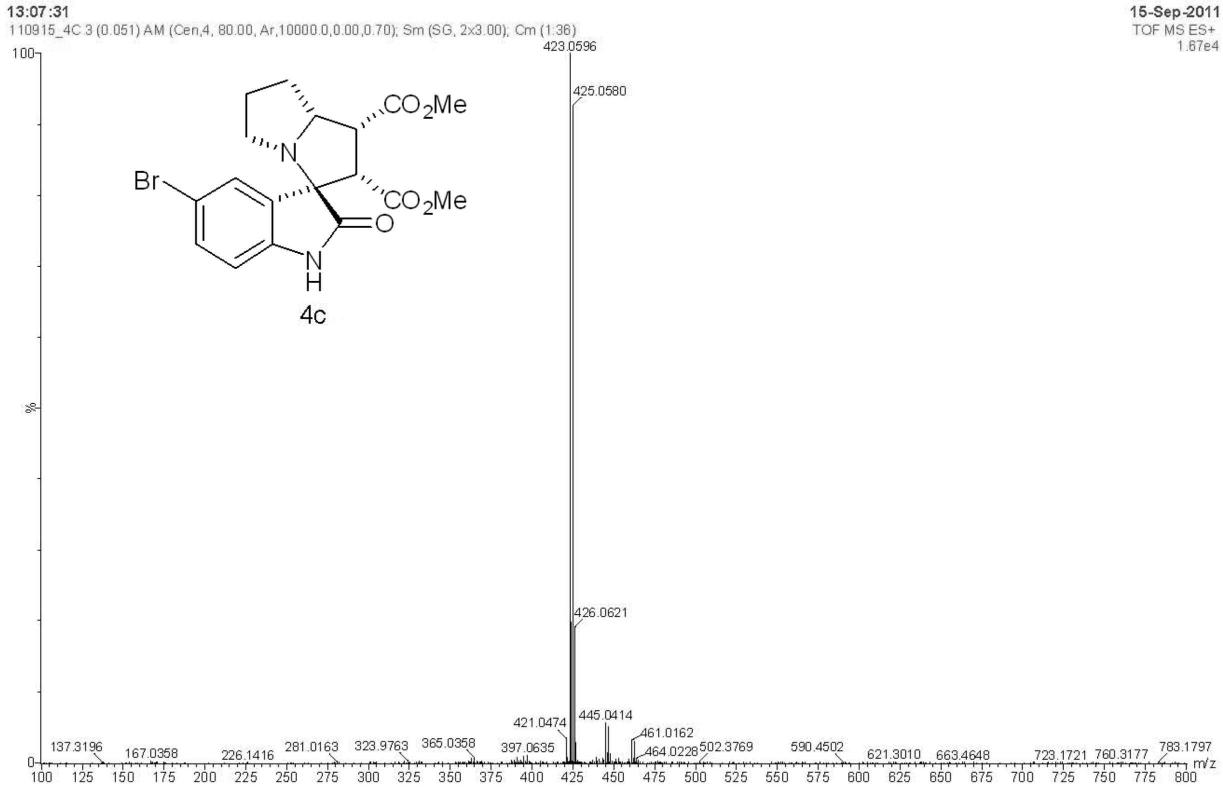
13:07:31
110915_4C 3 (0.051) AM (Cen,4, 80.00, Ar,10000.0,0.00,0.70); Sm (SG, 2x3.00); Cm (1:36)
15-Sep-2011
TOF MS ES+
1.67e4
4c
423.0596
425.0580
426.0621
445.0414
421.0474
461.0162
464.0228
m/z

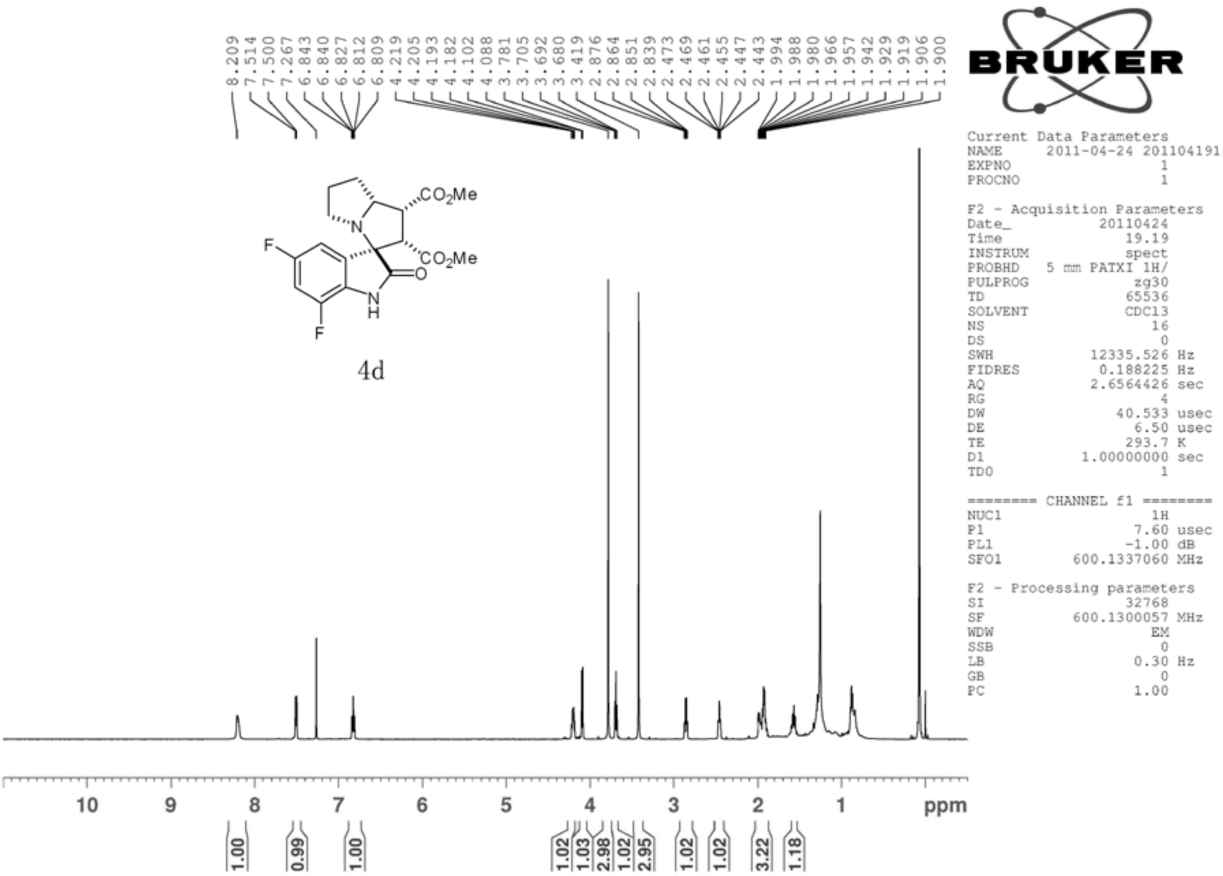
4d
BRUKER
Current Data Parameters
NAME 2011-04-24 201104191
EXPNO 1
PROCNO 1
F2 - Acquisition Parameters
Date_ 20110424
Time 19.19
INSTRUM spect
PROBHD 5 mm PATXI 1H/
PULPROG zg30
TD 65536
SOLVENT CDCl3
NS 16
DS 0
SWH 12335.526 Hz
FIDRES 0.188225 Hz
AQ 2.6564426 sec
RG 4
DW 40.533 usec
DE 6.50 usec
TE 293.7 K
D1 1.00000000 sec
TD0 1
======== CHANNEL f1 ========
NUC1 1H
P1 7.60 usec
PL1 -1.00 dB
SFO1 600.1337060 MHz
F2 - Processing parameters
SI 32768
SF 600.1300057 MHz
WDW EM
SSB 0
LB 0.30 Hz
GB 0
PC 1.00
ppm

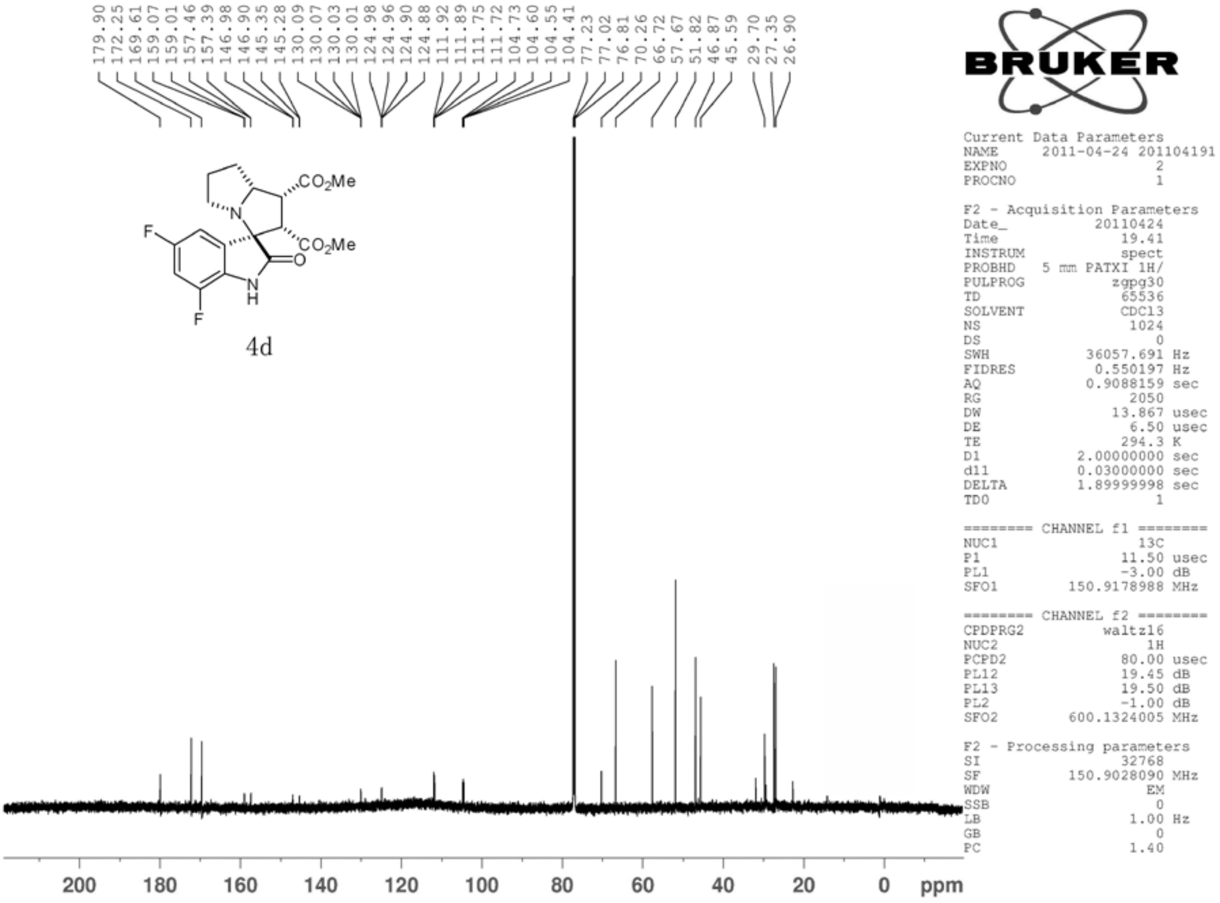
179.90
172.25
169.61
159.07
159.01
157.46
157.39
146.98
146.90
145.35
145.28
130.09
130.07
130.03
130.01
124.98
124.96
124.90
124.88
111.92
111.89
111.75
111.72
104.73
104.60
104.55
104.41
77.23
77.02
76.81
70.26
66.72
57.67
51.82
46.87
45.59
29.70
27.35
26.90
CO2Me
CO2Me
N
F
O
N
H
F
4d
BRUKER
Current Data Parameters
NAME 2011-04-24 201104191
EXPNO 2
PROCNO 1
F2 - Acquisition Parameters
Date_ 20110424
Time 19.41
INSTRUM spect
PROBHD 5 mm PATXI 1H/
PULPROG zgpg30
TD 65536
SOLVENT CDCl3
NS 1024
DS 0
SWH 36057.691 Hz
FIDRES 0.550197 Hz
AQ 0.9088159 sec
RG 2050
DW 13.867 usec
DE 6.50 usec
TE 294.3 K
D1 2.00000000 sec
d11 0.03000000 sec
DELTA 1.89999998 sec
TD0 1
======== CHANNEL f1 ========
NUC1 13C
P1 11.50 usec
PL1 -3.00 dB
SFO1 150.9178988 MHz
======== CHANNEL f2 ========
CPDPRG2 waltz16
NUC2 1H
PCPD2 80.00 usec
PL12 19.45 dB
PL13 19.50 dB
PL2 -1.00 dB
SFO2 600.1324005 MHz
F2 - Processing parameters
SI 32768
SF 150.9028090 MHz
WDW EM
SSB 0
LB 1.00 Hz
GB 0
PC 1.40
200 180 160 140 120 100 80 60 40 20 0 ppm

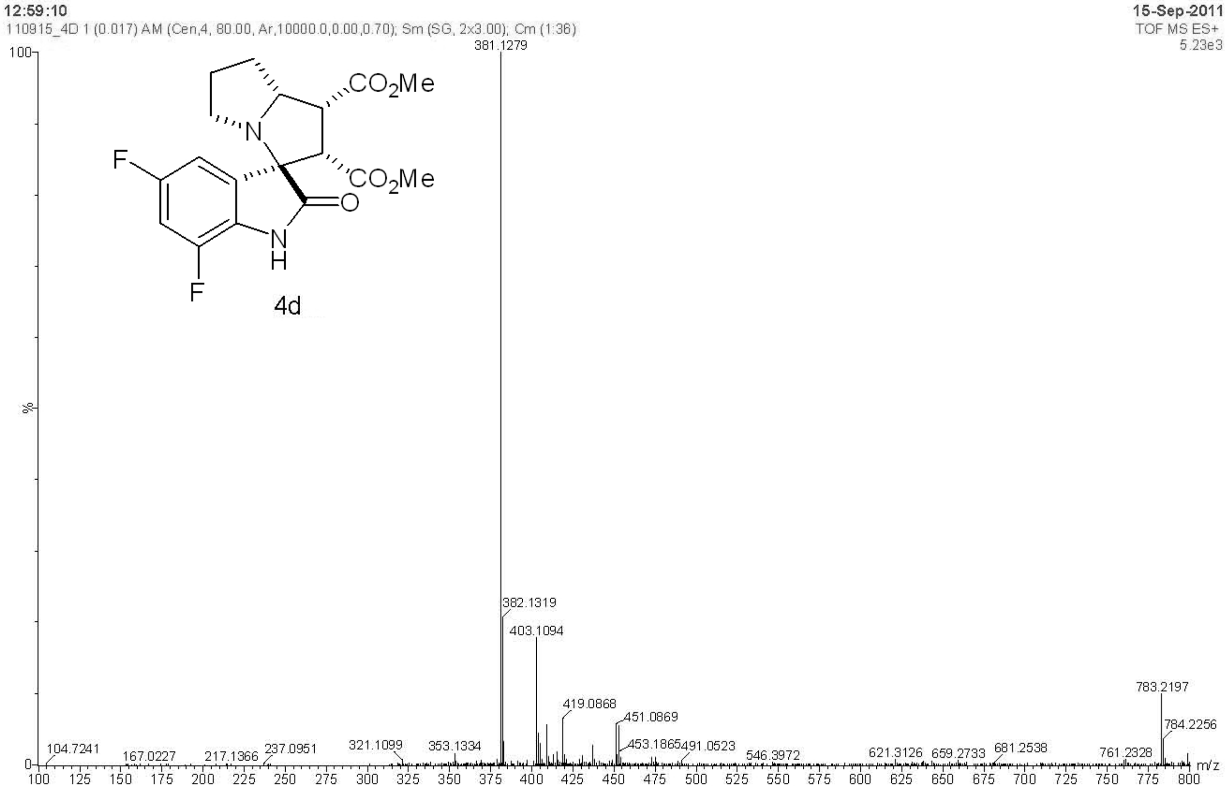
12:59:10
110915_4D 1 (0.017) AM (Cen,4, 80.00, Ar,10000.0,0.00,0.70); Sm (SG, 2x3.00); Cm (1:36)
15-Sep-2011
TOF MS ES+
5.23e3
100
%
0
381.1279
CO2Me
CO2Me
N
F
O
N
H
F
4d
382.1319
403.1094
419.0868
451.0869
453.1865
491.0523
546.3972
783.2197
784.2256
104.7241
167.0227
217.1366
237.0951
321.1099
353.1334
621.3126
659.2733
681.2538
761.2328
m/z
100 125 150 175 200 225 250 275 300 325 350 375 400 425 450 475 500 525 550 575 600 625 650 675 700 725 750 775 800

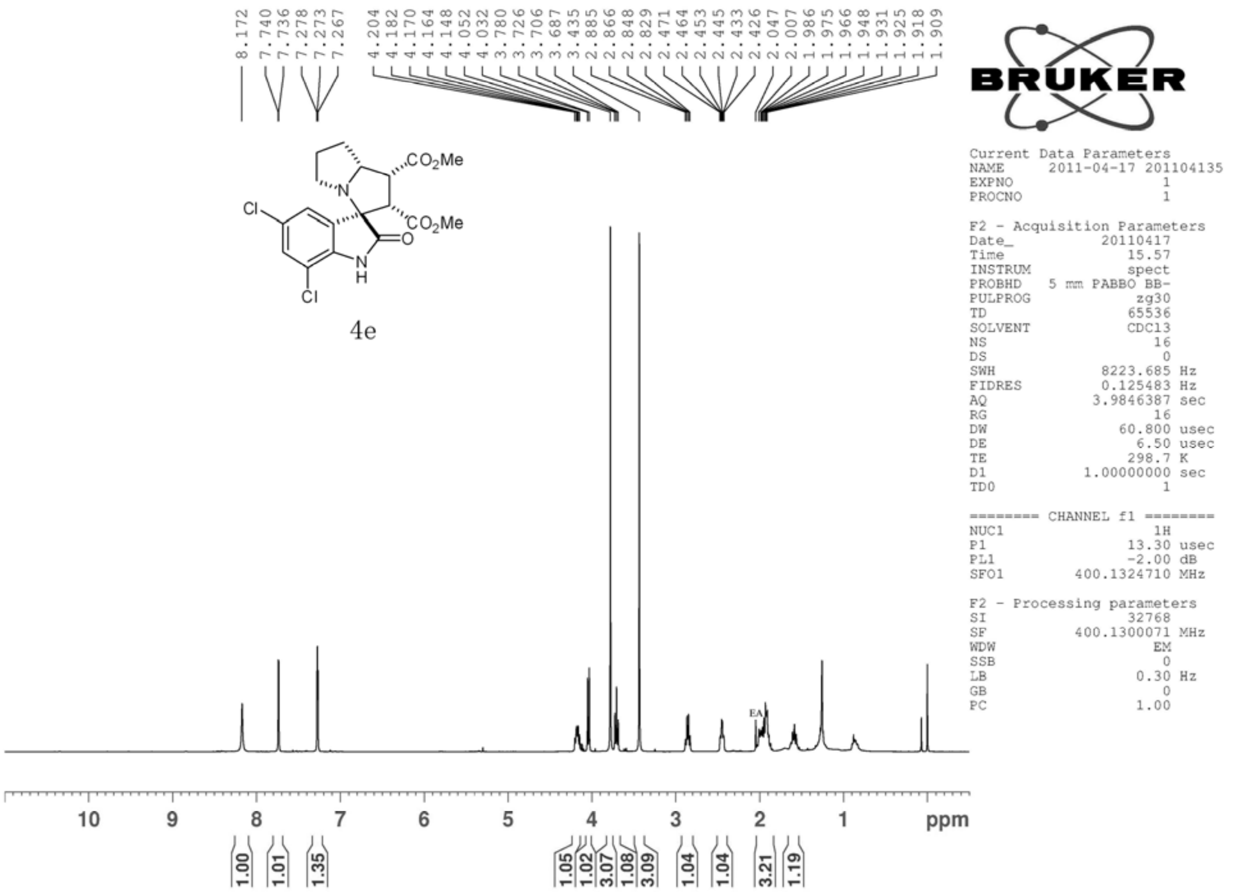
8.172
7.740
7.736
7.278
7.273
7.267
4.204
4.182
4.170
4.164
4.148
4.052
4.032
3.780
3.726
3.706
3.687
3.435
2.885
2.866
2.848
2.829
2.471
2.464
2.453
2.445
2.433
2.426
2.047
2.007
1.986
1.975
1.966
1.948
1.931
1.925
1.918
1.909
CO2Me
CO2Me
Cl
Cl
N
NH
O
4e
EA
10 9 8 7 6 5 4 3 2 1 ppm
1.00
1.01
1.35
1.05
1.02
3.07
1.08
3.09
1.04
1.04
3.21
1.19
BRUKER
Current Data Parameters
NAME 2011-04-17 201104135
EXPNO 1
PROCNO 1
F2 - Acquisition Parameters
Date_ 20110417
Time 15.57
INSTRUM spect
PROBHD 5 mm PABBO BB-
PULPROG zg30
TD 65536
SOLVENT CDCl3
NS 16
DS 0
SWH 8223.685 Hz
FIDRES 0.125483 Hz
AQ 3.9846387 sec
RG 16
DW 60.800 usec
DE 6.50 usec
TE 298.7 K
D1 1.00000000 sec
TD0 1
======== CHANNEL f1 ========
NUC1 1H
P1 13.30 usec
PL1 -2.00 dB
SFO1 400.1324710 MHz
F2 - Processing parameters
SI 32768
SF 400.1300071 MHz
WDW EM
SSB 0
LB 0.30 Hz
GB 0
PC 1.00

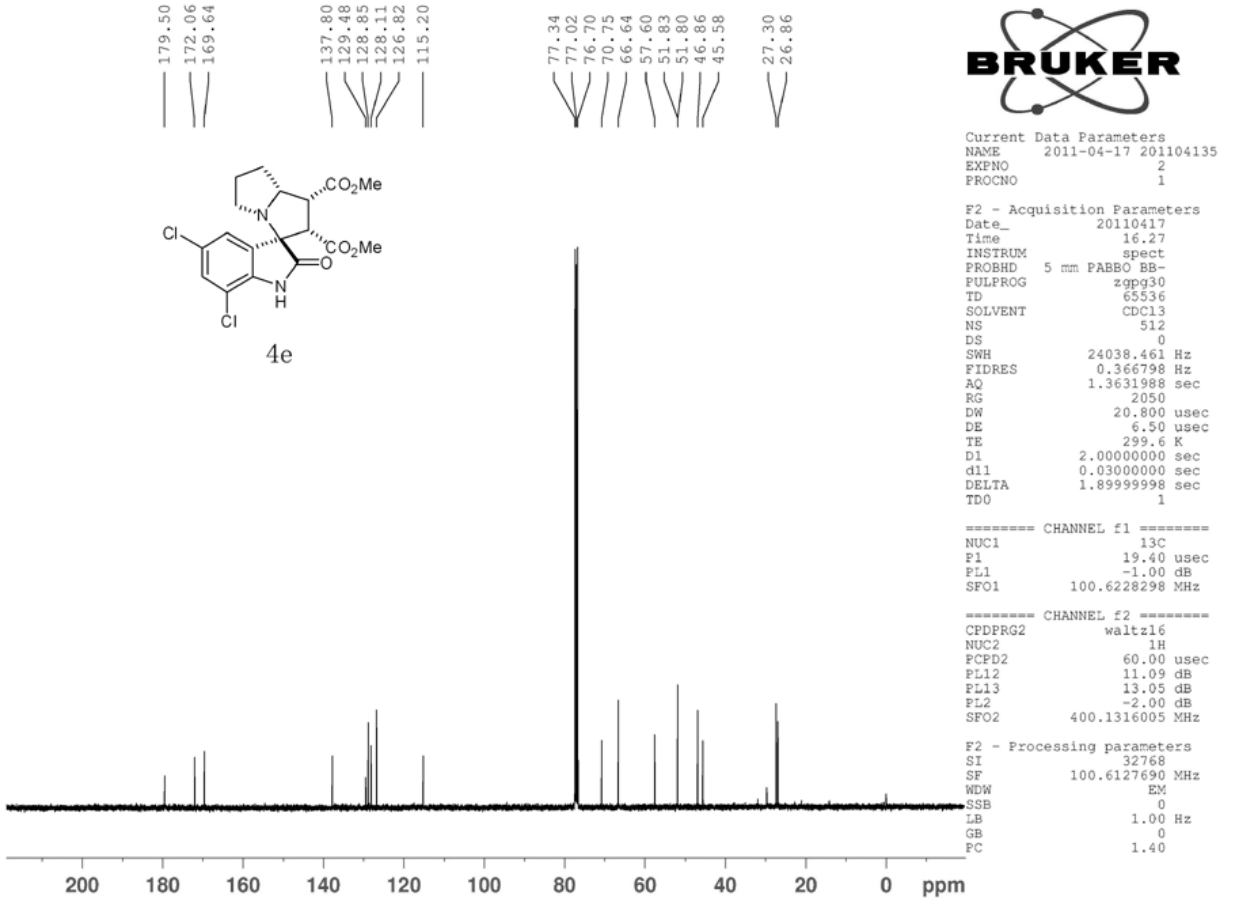
179.50
172.06
169.64
137.80
129.48
128.85
128.11
126.82
115.20
77.34
77.02
76.70
70.75
66.64
57.60
51.83
51.80
46.86
45.58
27.30
26.86
CO2Me
CO2Me
Cl
Cl
N
NH
O
4e
200 180 160 140 120 100 80 60 40 20 0 ppm
BRUKER
Current Data Parameters
NAME 2011-04-17 201104135
EXPNO 2
PROCNO 1
F2 - Acquisition Parameters
Date_ 20110417
Time 16.27
INSTRUM spect
PROBHD 5 mm PABBO BB-
PULPROG zgpg30
TD 65536
SOLVENT CDCl3
NS 512
DS 0
SWH 24038.461 Hz
FIDRES 0.366798 Hz
AQ 1.3631988 sec
RG 2050
DW 20.800 usec
DE 6.50 usec
TE 299.6 K
D1 2.00000000 sec
d11 0.03000000 sec
DELTA 1.89999998 sec
TD0 1
======== CHANNEL f1 ========
NUC1 13C
P1 19.40 usec
PL1 -1.00 dB
SFO1 100.6228298 MHz
======== CHANNEL f2 ========
CPDPRG2 waltz16
NUC2 1H
PCPD2 60.00 usec
PL12 11.09 dB
PL13 13.05 dB
PL2 -2.00 dB
SFO2 400.1316005 MHz
F2 - Processing parameters
SI 32768
SF 100.6127690 MHz
WDW EM
SSB 0
LB 1.00 Hz
GB 0
PC 1.40

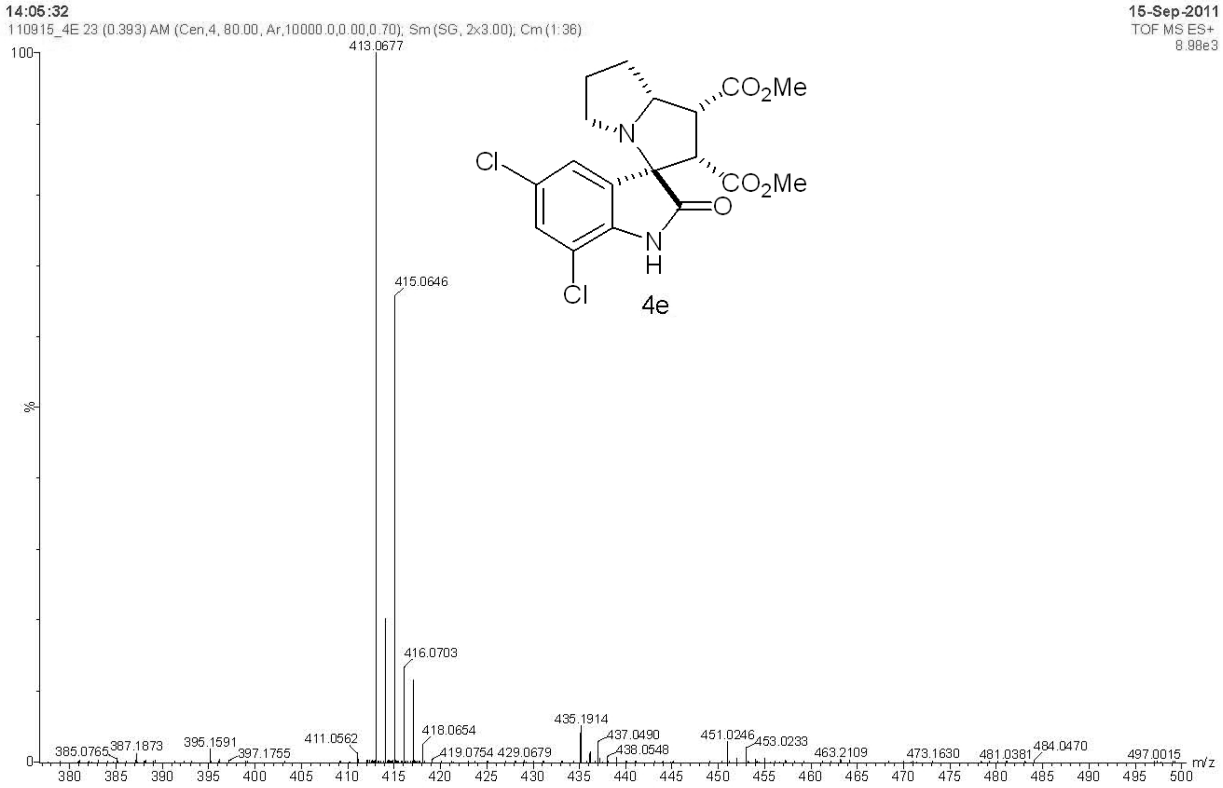
14:05:32
110915_4E 23 (0.393) AM (Cen,4, 80.00, Ar,10000.0,0.00,0.70); Sm (SG, 2x3.00); Cm (1:36)
15-Sep-2011
TOF MS ES+
8.98e3
413.0677
415.0646
416.0703
418.0654
419.0754
429.0679
435.1914
437.0490
438.0548
451.0246
453.0233
463.2109
473.1630
481.0381
484.0470
497.0015
385.0765
387.1873
395.1591
397.1755
411.0562
m/z
%
CO2Me
CO2Me
Cl
Cl
N
N
H
O
4e

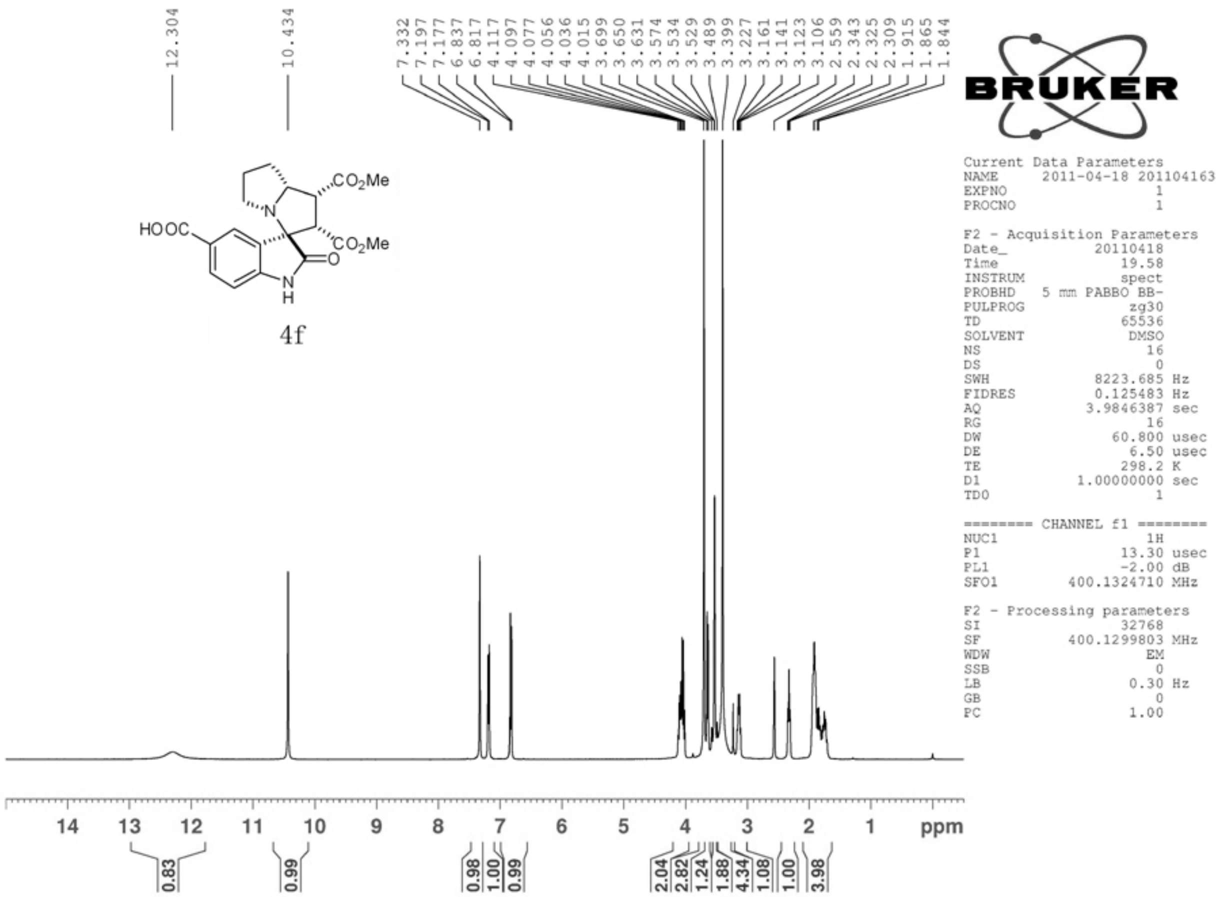
12.304
10.434
7.332 7.197 7.177 6.837 6.817 4.117 4.097 4.077 4.056 4.036 4.015 3.699 3.650 3.631 3.574 3.534 3.529 3.489 3.399 3.227 3.161 3.141 3.123 3.106 2.559 2.343 2.325 2.309 1.915 1.865 1.844
HOOC
CO2Me
CO2Me
N
N
H
O
4f
BRUKER
Current Data Parameters
NAME 2011-04-18 201104163
EXPNO 1
PROCNO 1
F2 - Acquisition Parameters
Date_ 20110418
Time 19.58
INSTRUM spect
PROBHD 5 mm PABBO BB-
PULPROG zg30
TD 65536
SOLVENT DMSO
NS 16
DS 0
SWH 8223.685 Hz
FIDRES 0.125483 Hz
AQ 3.9846387 sec
RG 16
DW 60.800 usec
DE 6.50 usec
TE 298.2 K
D1 1.00000000 sec
TD0 1
======== CHANNEL f1 ========
NUC1 1H
P1 13.30 usec
PL1 -2.00 dB
SFO1 400.1324710 MHz
F2 - Processing parameters
SI 32768
SF 400.1299803 MHz
WDW EM
SSB 0
LB 0.30 Hz
GB 0
PC 1.00
ppm
0.83
0.99
0.98
1.00
0.99
2.04
2.82
1.24
1.88
4.34
1.08
1.00
3.98

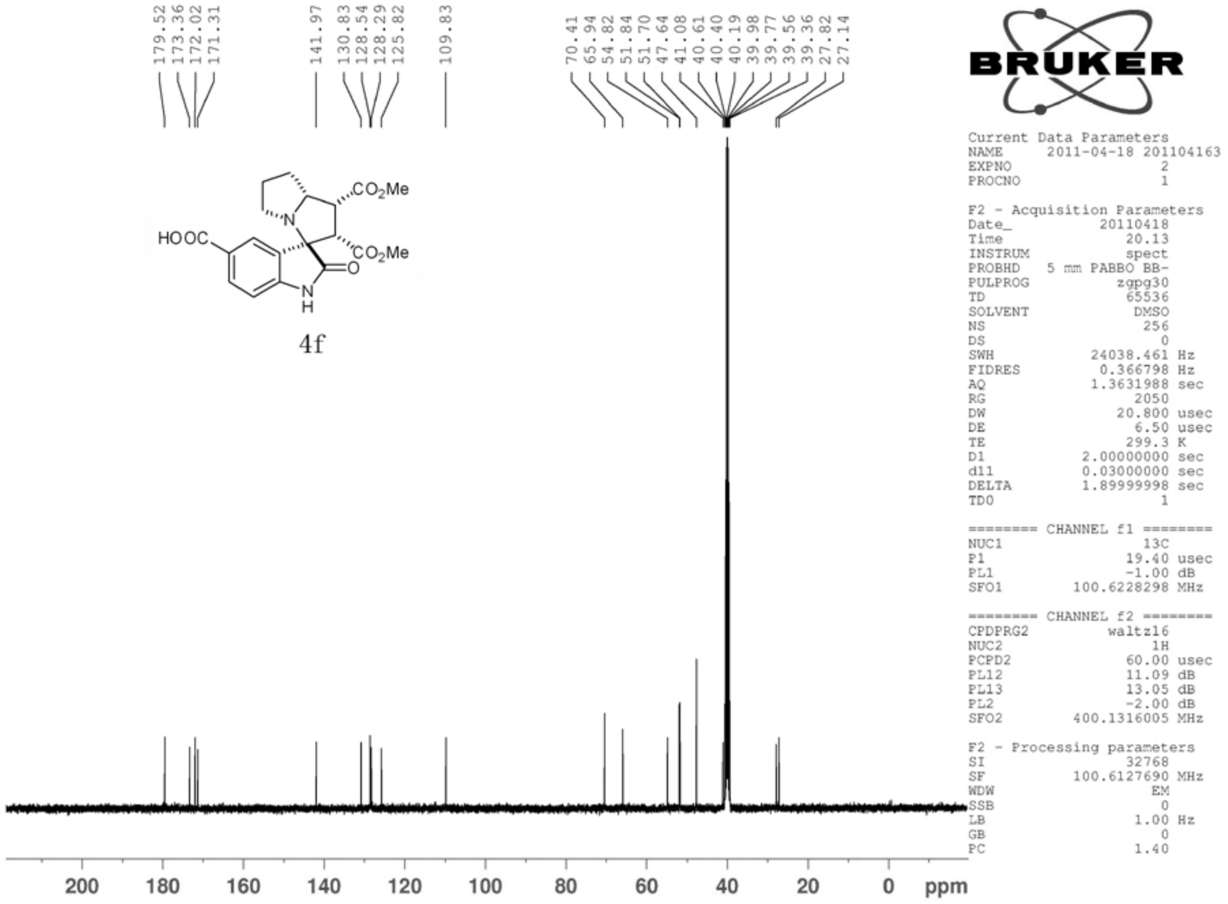
179.52
173.36
172.02
171.31
141.97
130.83
128.54
128.29
125.82
109.83
70.41
65.94
54.82
51.84
51.70
47.64
41.08
40.61
40.40
40.19
39.98
39.77
39.56
39.36
27.82
27.14
CO2Me
HOOC
CO2Me
O
N
H
4f
BRUKER
Current Data Parameters
NAME 2011-04-18 201104163
EXPNO 2
PROCNO 1
F2 - Acquisition Parameters
Date_ 20110418
Time 20.13
INSTRUM spect
PROBHD 5 mm PABBO BB-
PULPROG zgpg30
TD 65536
SOLVENT DMSO
NS 256
DS 0
SWH 24038.461 Hz
FIDRES 0.366798 Hz
AQ 1.3631988 sec
RG 2050
DW 20.800 usec
DE 6.50 usec
TE 299.3 K
D1 2.00000000 sec
d11 0.03000000 sec
DELTA 1.89999998 sec
TD0 1
======== CHANNEL f1 ========
NUC1 13C
P1 19.40 usec
PL1 -1.00 dB
SFO1 100.6228298 MHz
======== CHANNEL f2 ========
CPDPRG2 waltz16
NUC2 1H
PCPD2 60.00 usec
PL12 11.09 dB
PL13 13.05 dB
PL2 -2.00 dB
SFO2 400.1316005 MHz
F2 - Processing parameters
SI 32768
SF 100.6127690 MHz
WDW EM
SSB 0
LB 1.00 Hz
GB 0
PC 1.40
200 180 160 140 120 100 80 60 40 20 0 ppm

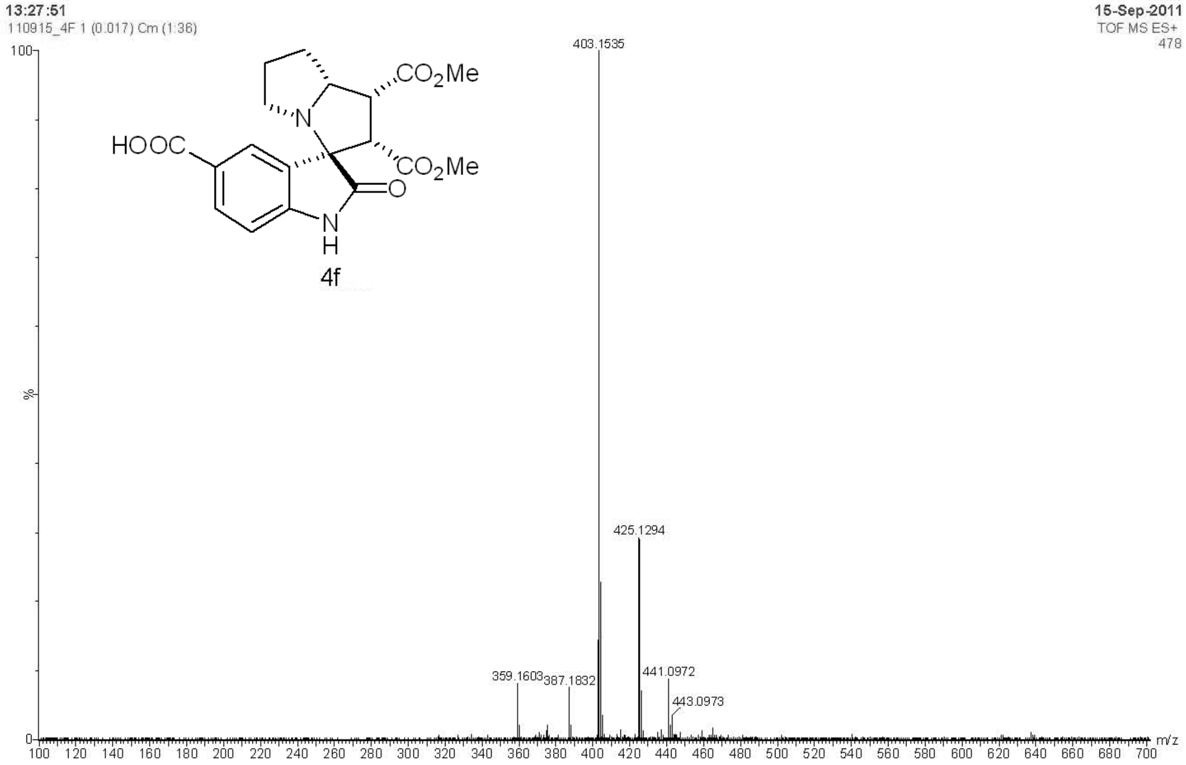
13:27:51
110915_4F 1 (0.017) Cm (1:36)
15-Sep-2011
TOF MS ES+
478
100
%
0
CO2Me
HOOC
CO2Me
O
N
H
4f
403.1535
425.1294
359.1603
387.1832
441.0972
443.0973
m/z
100 120 140 160 180 200 220 240 260 280 300 320 340 360 380 400 420 440 460 480 500 520 540 560 580 600 620 640 660 680 700

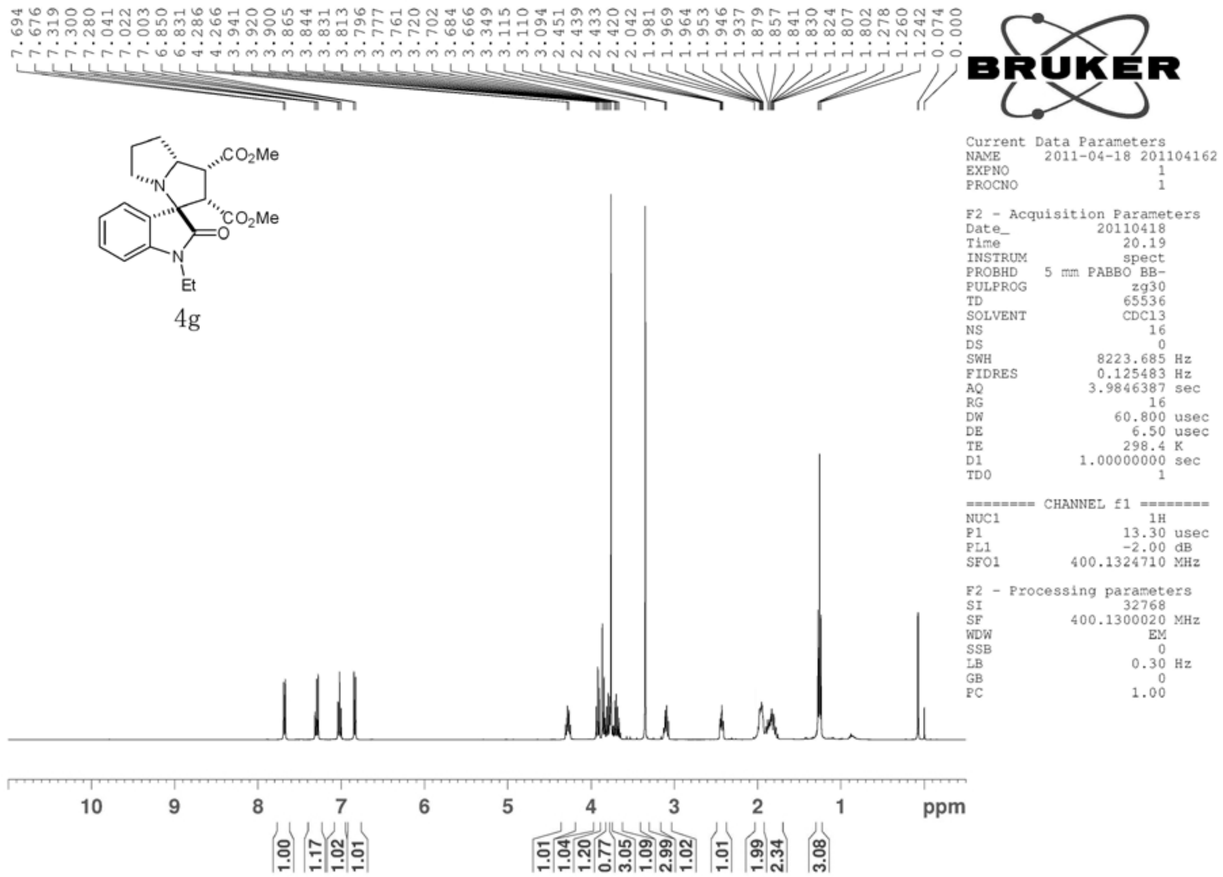
7.694
7.676
7.319
7.300
7.280
7.041
7.022
7.003
6.850
6.831
4.286
4.266
3.941
3.920
3.900
3.865
3.844
3.831
3.813
3.796
3.777
3.761
3.720
3.702
3.684
3.666
3.349
3.115
3.110
3.094
2.451
2.439
2.433
2.420
2.042
1.981
1.969
1.964
1.953
1.946
1.937
1.879
1.857
1.841
1.830
1.824
1.807
1.802
1.278
1.260
1.242
0.074
0.000
BRUKER
CO2Me
CO2Me
N
O
N
Et
4g
Current Data Parameters
NAME 2011-04-18 201104162
EXPNO 1
PROCNO 1
F2 - Acquisition Parameters
Date_ 20110418
Time 20.19
INSTRUM spect
PROBHD 5 mm PABBO BB-
PULPROG zg30
TD 65536
SOLVENT CDCl3
NS 16
DS 0
SWH 8223.685 Hz
FIDRES 0.125483 Hz
AQ 3.9846387 sec
RG 16
DW 60.800 usec
DE 6.50 usec
TE 298.4 K
D1 1.00000000 sec
TD0 1
======== CHANNEL f1 ========
NUC1 1H
P1 13.30 usec
PL1 -2.00 dB
SFO1 400.1324710 MHz
F2 - Processing parameters
SI 32768
SF 400.1300020 MHz
WDW EM
SSB 0
LB 0.30 Hz
GB 0
PC 1.00
10 9 8 7 6 5 4 3 2 1 ppm
1.00 1.17 1.02 1.01
1.01 1.04 1.20 0.77 3.05 1.09 2.99 1.02 1.01 1.99 2.34 3.08

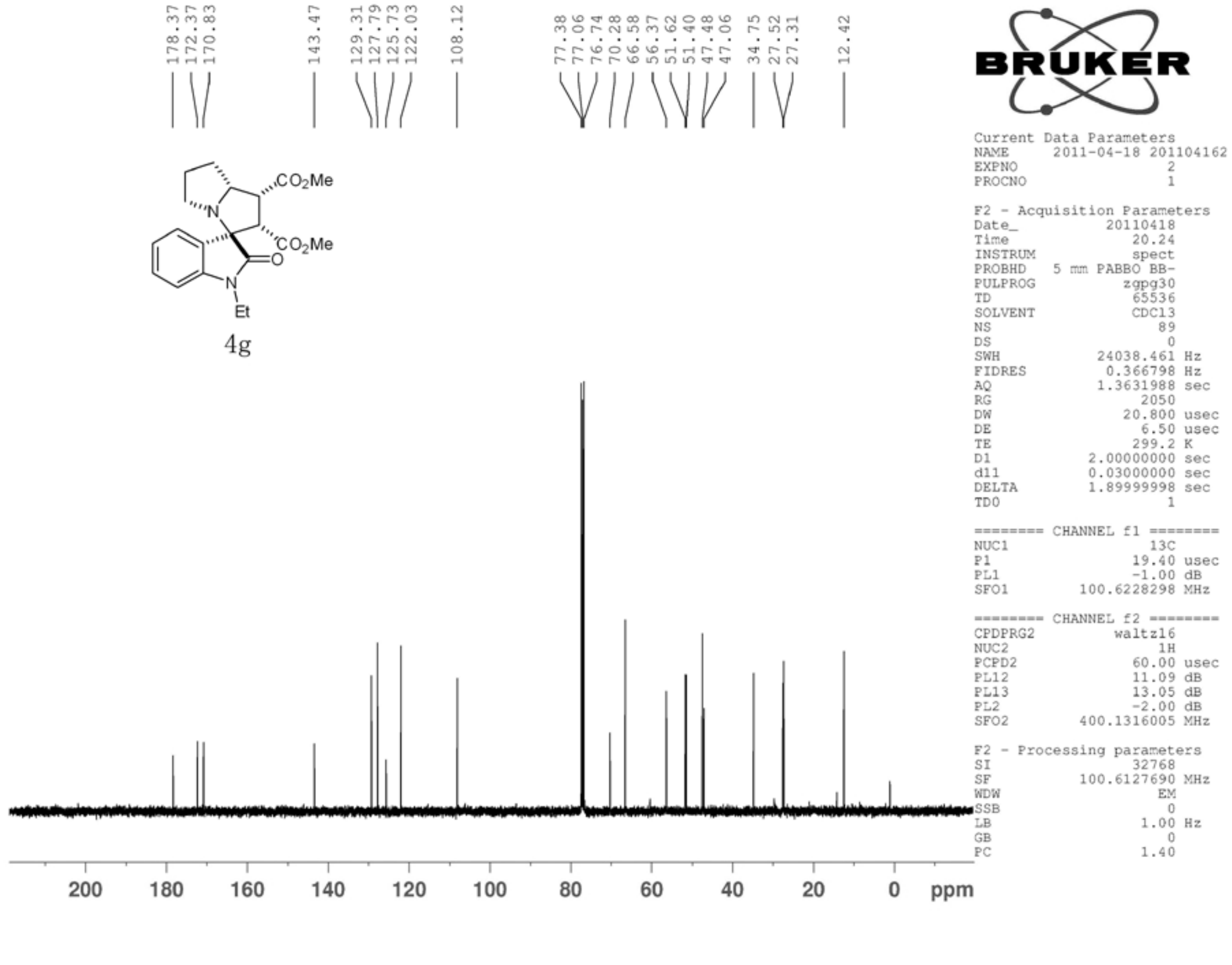
178.37
172.37
170.83
143.47
129.31
127.79
125.73
122.03
108.12
77.38
77.06
76.74
70.28
66.58
56.37
51.62
51.40
47.48
47.06
34.75
27.52
27.31
12.42
BRUKER
CO2Me
CO2Me
N
O
N
Et
4g
Current Data Parameters
NAME 2011-04-18 201104162
EXPNO 2
PROCNO 1
F2 - Acquisition Parameters
Date_ 20110418
Time 20.24
INSTRUM spect
PROBHD 5 mm PABBO BB-
PULPROG zgpg30
TD 65536
SOLVENT CDCl3
NS 89
DS 0
SWH 24038.461 Hz
FIDRES 0.366798 Hz
AQ 1.3631988 sec
RG 2050
DW 20.800 usec
DE 6.50 usec
TE 299.2 K
D1 2.00000000 sec
d11 0.03000000 sec
DELTA 1.89999998 sec
TD0 1
======== CHANNEL f1 ========
NUC1 13C
P1 19.40 usec
PL1 -1.00 dB
SFO1 100.6228298 MHz
======== CHANNEL f2 ========
CPDPRG2 waltz16
NUC2 1H
PCPD2 60.00 usec
PL12 11.09 dB
PL13 13.05 dB
PL2 -2.00 dB
SFO2 400.1316005 MHz
F2 - Processing parameters
SI 32768
SF 100.6127690 MHz
WDW EM
SSB 0
LB 1.00 Hz
GB 0
PC 1.40
200 180 160 140 120 100 80 60 40 20 0 ppm

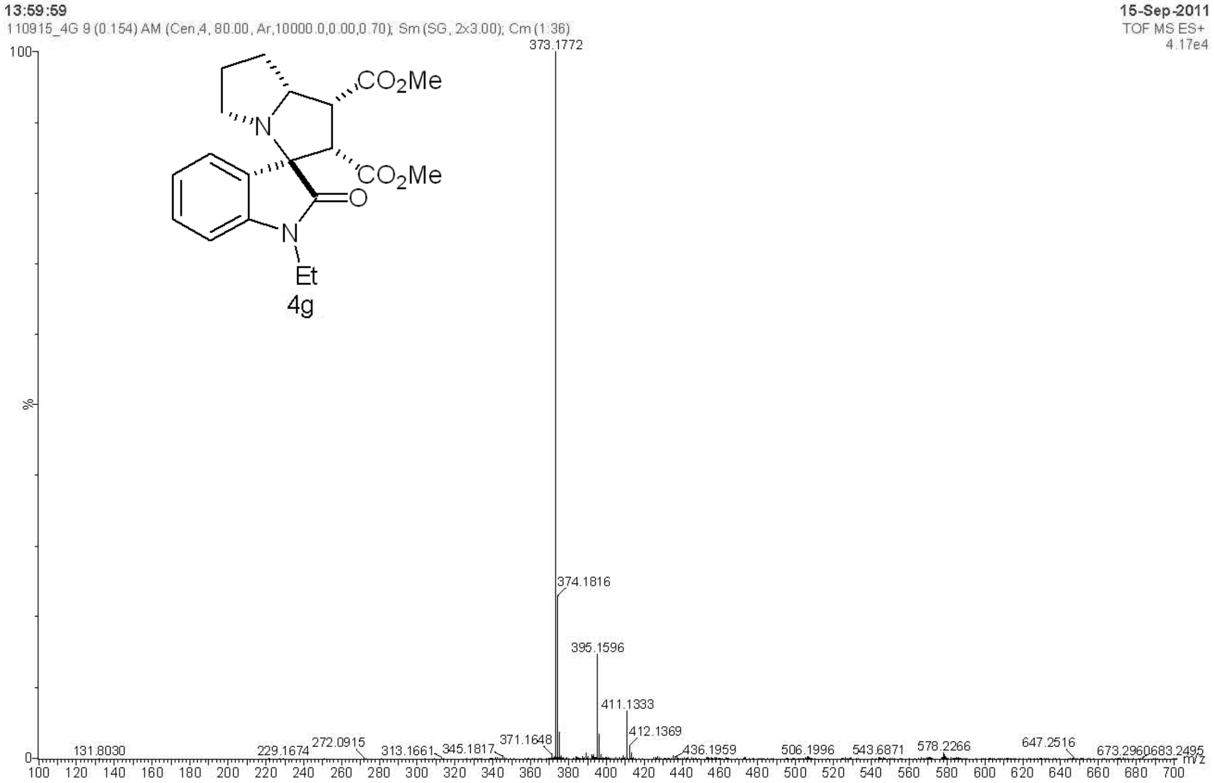
13:59:59
110915_4G 9 (0.154) AM (Cen,4, 80.00, Ar,10000.0,0.00,0.70); Sm (SG, 2x3.00); Cm (1:36)
15-Sep-2011
TOF MS ES+
4.17e4
373.1772
374.1816
395.1596
411.1333
412.1369
CO2Me
CO2Me
N
O
N
Et
4g
%
m/z

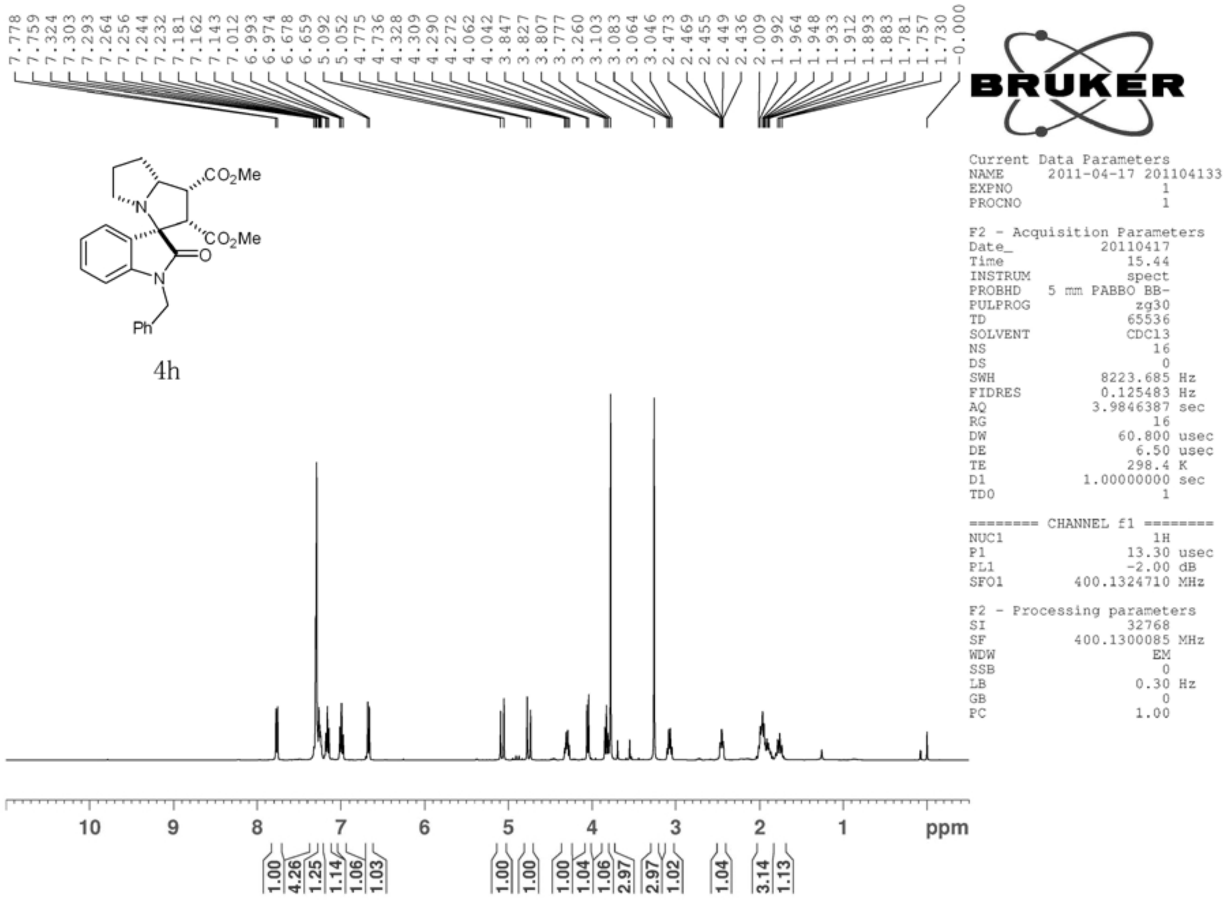
BRUKER
Current Data Parameters
NAME 2011-04-17 201104133
EXPNO 1
PROCNO 1
F2 - Acquisition Parameters
Date_ 20110417
Time 15.44
INSTRUM spect
PROBHD 5 mm PABBO BB-
PULPROG zg30
TD 65536
SOLVENT CDCl3
NS 16
DS 0
SWH 8223.685 Hz
FIDRES 0.125483 Hz
AQ 3.9846387 sec
RG 16
DW 60.800 usec
DE 6.50 usec
TE 298.4 K
D1 1.00000000 sec
TD0 1
======== CHANNEL f1 ========
NUC1 1H
P1 13.30 usec
PL1 -2.00 dB
SFO1 400.1324710 MHz
F2 - Processing parameters
SI 32768
SF 400.1300085 MHz
WDW EM
SSB 0
LB 0.30 Hz
GB 0
PC 1.00
CO2Me
CO2Me
N
O
N
Ph
4h
ppm

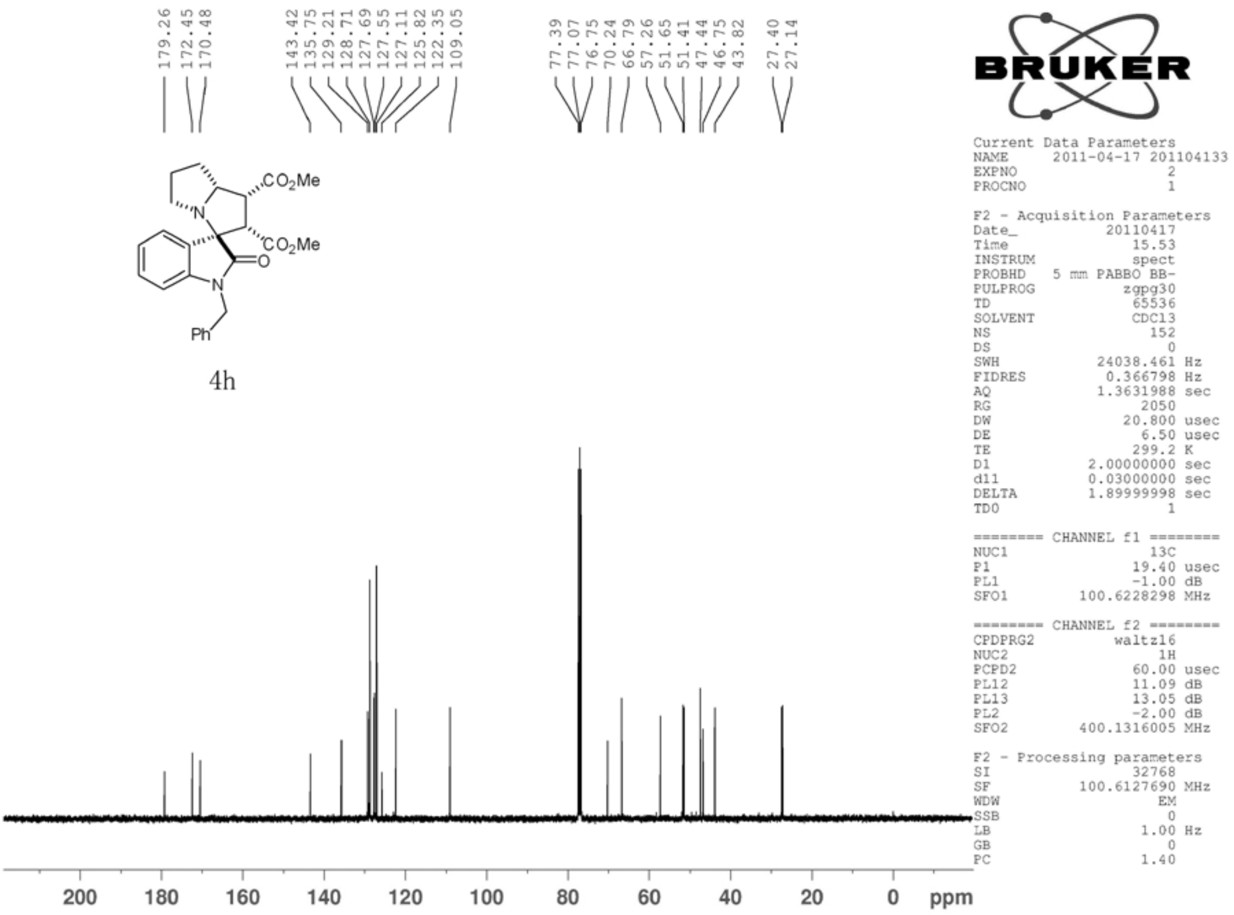

179.26
172.45
170.48
143.42
135.75
129.21
128.71
127.69
127.55
127.11
125.82
122.35
109.05
77.39
77.07
76.75
70.24
66.79
57.26
51.65
51.41
47.44
46.75
43.82
27.40
27.14
CO2Me
CO2Me
N
O
N
Ph
4h
BRUKER
Current Data Parameters
NAME 2011-04-17 201104133
EXPNO 2
PROCNO 1
F2 - Acquisition Parameters
Date_ 20110417
Time 15.53
INSTRUM spect
PROBHD 5 mm PABBO BB-
PULPROG zgpg30
TD 65536
SOLVENT CDCl3
NS 152
DS 0
SWH 24038.461 Hz
FIDRES 0.366798 Hz
AQ 1.3631988 sec
RG 2050
DW 20.800 usec
DE 6.50 usec
TE 299.2 K
D1 2.00000000 sec
d11 0.03000000 sec
DELTA 1.89999998 sec
TD0 1
======== CHANNEL f1 ========
NUC1 13C
P1 19.40 usec
PL1 -1.00 dB
SFO1 100.6228298 MHz
======== CHANNEL f2 ========
CPDPRG2 waltz16
NUC2 1H
PCPD2 60.00 usec
PL12 11.09 dB
PL13 13.05 dB
PL2 -2.00 dB
SFO2 400.1316005 MHz
F2 - Processing parameters
SI 32768
SF 100.6127690 MHz
WDW EM
SSB 0
LB 1.00 Hz
GB 0
PC 1.40
200 180 160 140 120 100 80 60 40 20 0 ppm

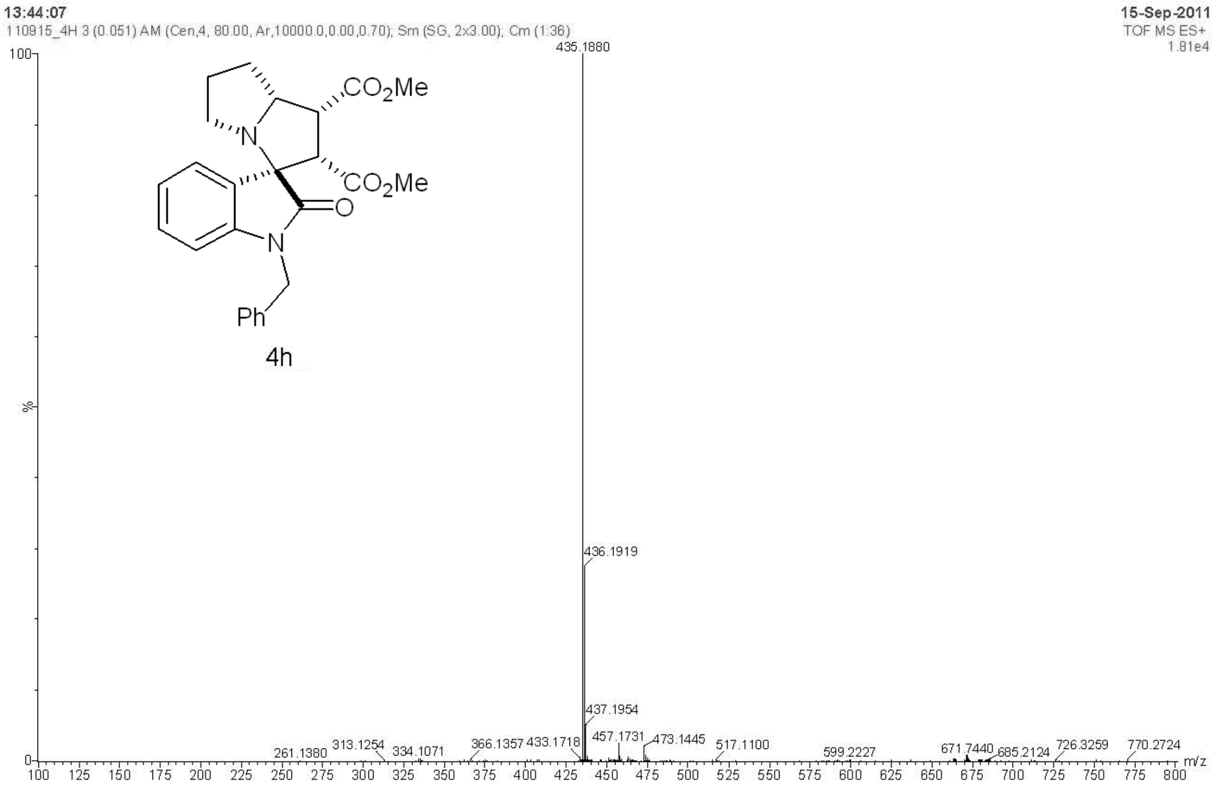

13:44:07
110915_4H 3 (0.051) AM (Cen,4, 80.00, Ar,10000.0,0.00,0.70); Sm (SG, 2x3.00); Cm (1:36)
15-Sep-2011
TOF MS ES+
1.81e4
435.1880
436.1919
437.1954
261.1380
313.1254
334.1071
366.1357
433.1718
457.1731
473.1445
517.1100
599.2227
671.7440
685.2124
726.3259
770.2724
CO2Me
CO2Me
N
O
N
Ph
4h
100
%
0
100 125 150 175 200 225 250 275 300 325 350 375 400 425 450 475 500 525 550 575 600 625 650 675 700 725 750 775 800 m/z

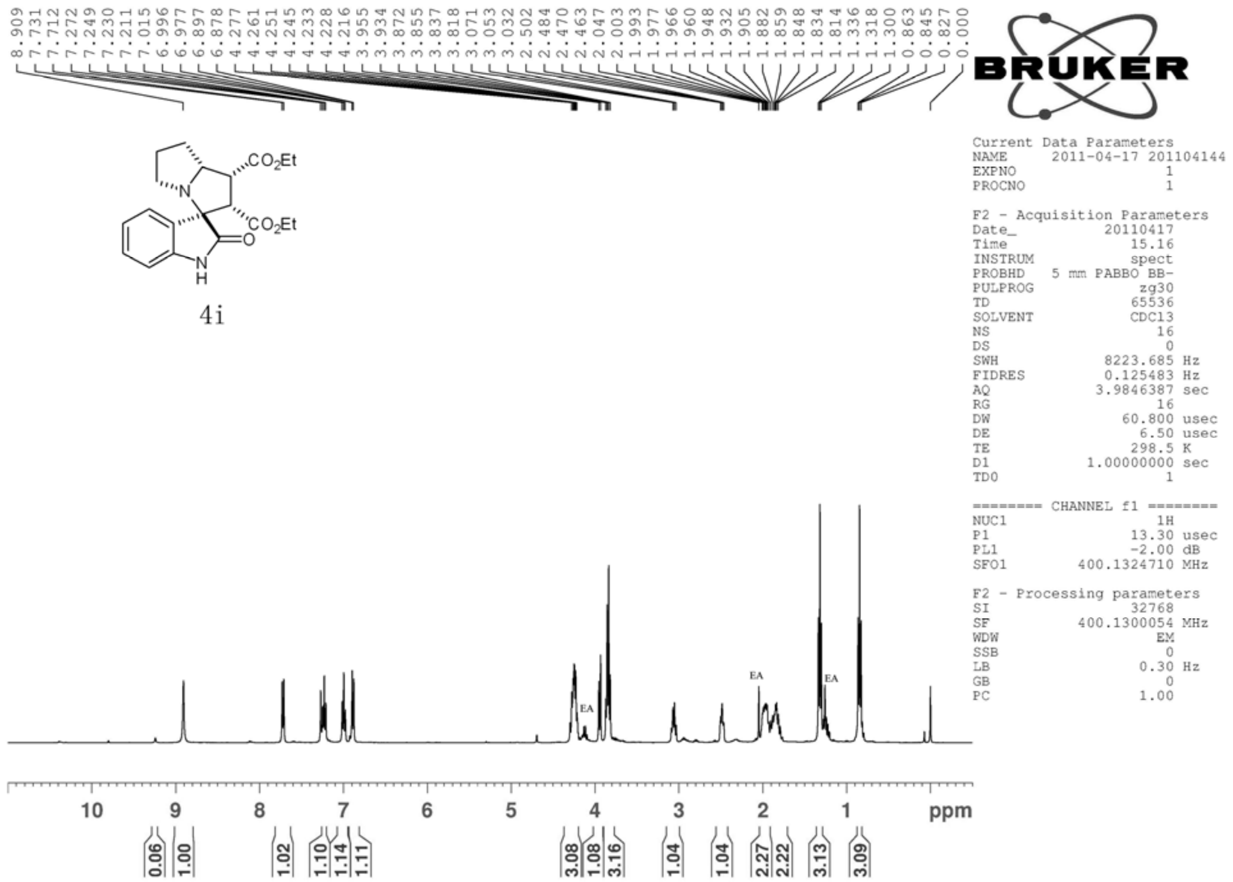
8.909
7.731
7.712
7.272
7.249
7.230
7.211
7.015
6.996
6.977
6.897
6.878
4.277
4.261
4.251
4.245
4.233
4.228
4.216
3.955
3.934
3.872
3.855
3.837
3.818
3.071
3.053
3.032
2.502
2.484
2.470
2.463
2.047
2.003
1.993
1.977
1.966
1.960
1.948
1.932
1.905
1.882
1.859
1.848
1.834
1.814
1.336
1.318
1.300
0.863
0.845
0.827
0.000
BRUKER
CO2Et
CO2Et
N
O
N
H
4i
EA
EA
EA
10
9
8
7
6
5
4
3
2
1
ppm
0.06
1.00
1.02
1.10
1.14
1.11
3.08
1.08
3.16
1.04
1.04
2.27
2.22
3.13
3.09
Current Data Parameters
NAME 2011-04-17 201104144
EXPNO 1
PROCNO 1
F2 - Acquisition Parameters
Date_ 20110417
Time 15.16
INSTRUM spect
PROBHD 5 mm PABBO BB-
PULPROG zg30
TD 65536
SOLVENT CDCl3
NS 16
DS 0
SWH 8223.685 Hz
FIDRES 0.125483 Hz
AQ 3.9846387 sec
RG 16
DW 60.800 usec
DE 6.50 usec
TE 298.5 K
D1 1.00000000 sec
TD0 1
======== CHANNEL f1 ========
NUC1 1H
P1 13.30 usec
PL1 -2.00 dB
SFO1 400.1324710 MHz
F2 - Processing parameters
SI 32768
SF 400.1300054 MHz
WDW EM
SSB 0
LB 0.30 Hz
GB 0
PC 1.00

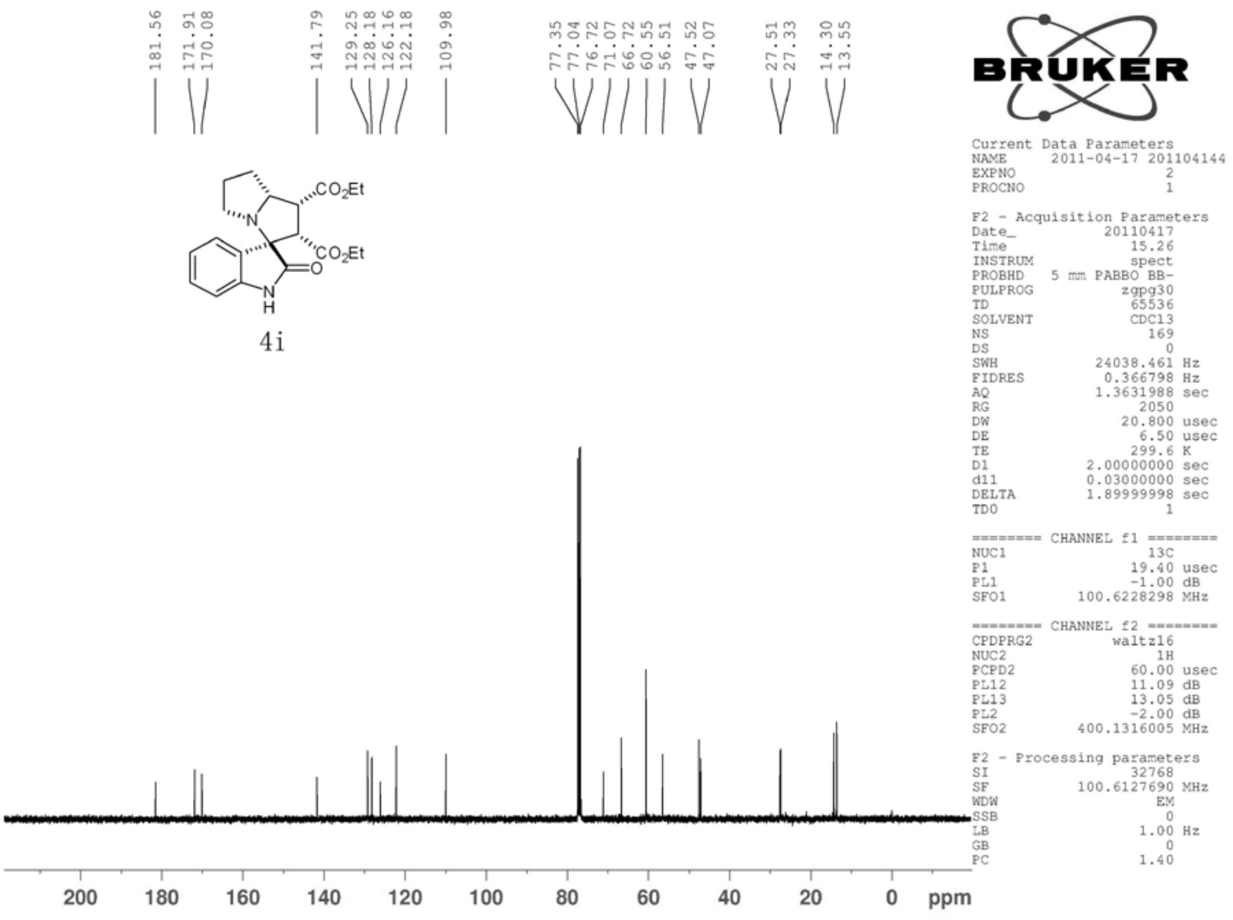
181.56
171.91
170.08
141.79
129.25
128.18
126.16
122.18
109.98
77.35
77.04
76.72
71.07
66.72
60.55
56.51
47.52
47.07
27.51
27.33
14.30
13.55
BRUKER
CO2Et
CO2Et
N
O
N
H
4i
200
180
160
140
120
100
80
60
40
20
0
ppm
Current Data Parameters
NAME 2011-04-17 201104144
EXPNO 2
PROCNO 1
F2 - Acquisition Parameters
Date_ 20110417
Time 15.26
INSTRUM spect
PROBHD 5 mm PABBO BB-
PULPROG zgpg30
TD 65536
SOLVENT CDCl3
NS 169
DS 0
SWH 24038.461 Hz
FIDRES 0.366798 Hz
AQ 1.3631988 sec
RG 2050
DW 20.800 usec
DE 6.50 usec
TE 299.6 K
D1 2.00000000 sec
d11 0.03000000 sec
DELTA 1.89999998 sec
TD0 1
======== CHANNEL f1 ========
NUC1 13C
P1 19.40 usec
PL1 -1.00 dB
SFO1 100.6228298 MHz
======== CHANNEL f2 ========
CPDPRG2 waltz16
NUC2 1H
PCPD2 60.00 usec
PL12 11.09 dB
PL13 13.05 dB
PL2 -2.00 dB
SFO2 400.1316005 MHz
F2 - Processing parameters
SI 32768
SF 100.6127690 MHz
WDW EM
SSB 0
LB 1.00 Hz
GB 0
PC 1.40

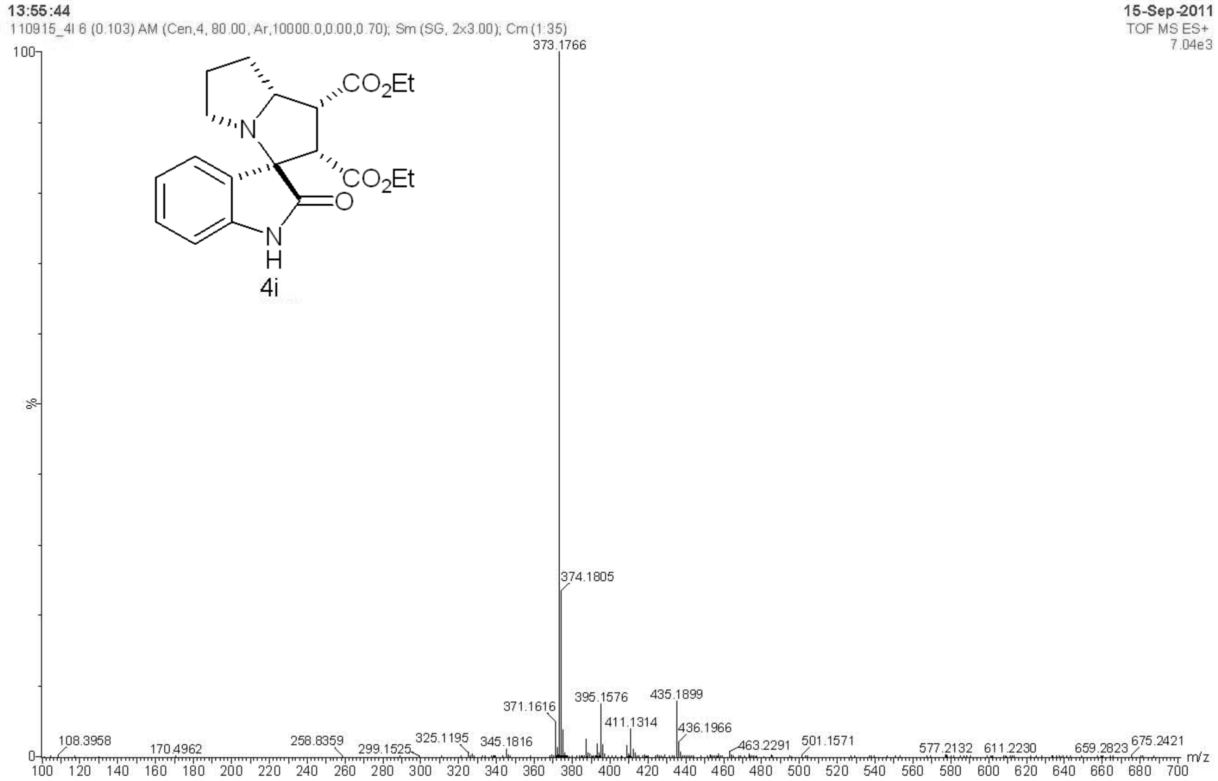
13:55:44
15-Sep-2011
110915_4l 6 (0.103) AM (Cen,4, 80.00, Ar,10000.0,0.00,0.70); Sm (SG, 2x3.00); Cm (1:35)
TOF MS ES+
7.04e3
CO2Et
CO2Et
N
O
N
H
4i
373.1766
374.1805
371.1616
395.1576
435.1899
411.1314
436.1966
108.3958
170.4962
258.8359
299.1525
325.1195
345.1816
463.2291
501.1571
577.2132
611.2230
659.2823
675.2421
%
m/z

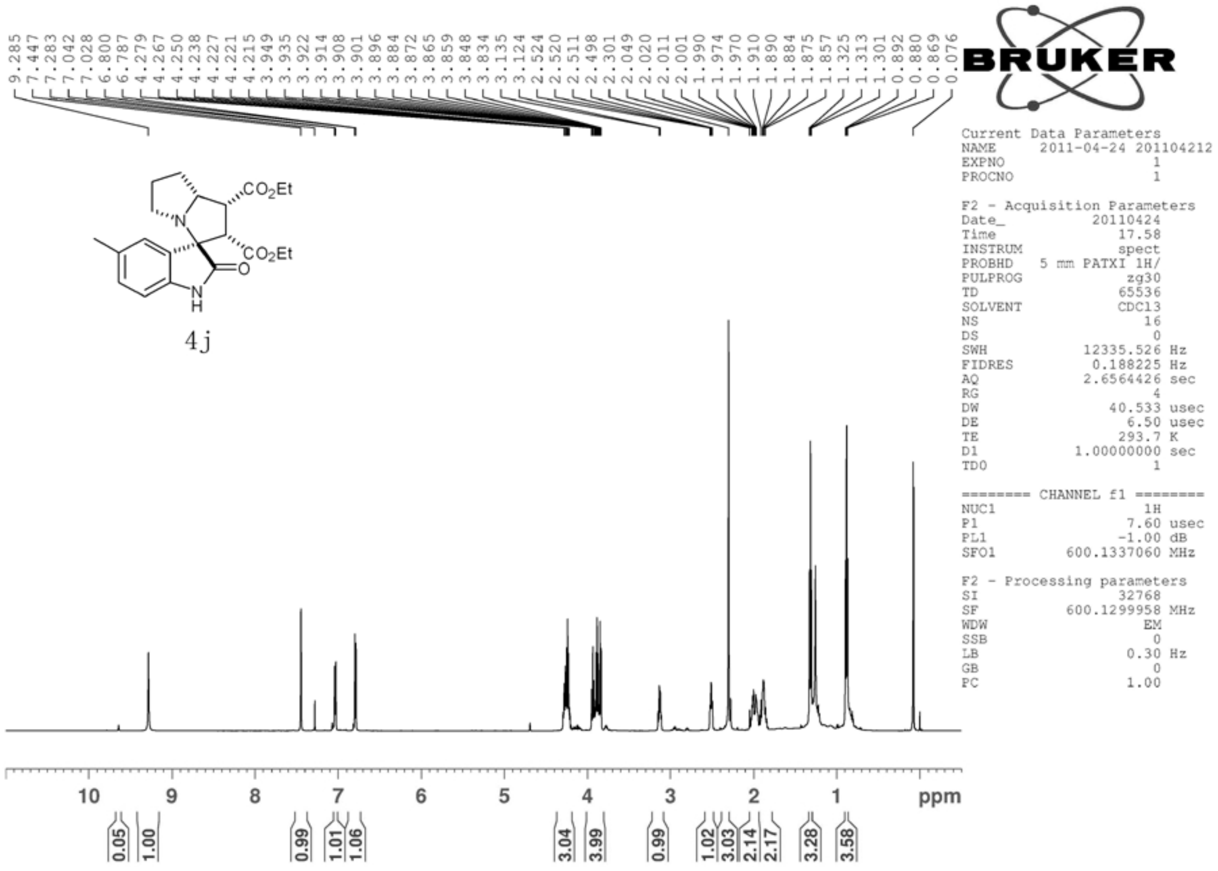
9.285 7.447 7.283 7.042 7.028 6.800 6.787 4.279 4.267 4.250 4.238 4.227 4.221 4.215 3.949 3.935 3.922 3.914 3.908 3.901 3.896 3.884 3.872 3.865 3.859 3.848 3.834 3.135 3.124 2.524 2.520 2.511 2.498 2.301 2.049 2.020 2.011 2.001 1.990 1.974 1.970 1.910 1.890 1.884 1.875 1.857 1.325 1.313 1.301 0.892 0.880 0.869 0.076
BRUKER
CO2Et
CO2Et
N
O
N
H
4j
Current Data Parameters
NAME 2011-04-24 201104212
EXPNO 1
PROCNO 1
F2 - Acquisition Parameters
Date_ 20110424
Time 17.58
INSTRUM spect
PROBHD 5 mm PATXI 1H/
PULPROG zg30
TD 65536
SOLVENT CDCl3
NS 16
DS 0
SWH 12335.526 Hz
FIDRES 0.188225 Hz
AQ 2.6564426 sec
RG 4
DW 40.533 usec
DE 6.50 usec
TE 293.7 K
D1 1.00000000 sec
TD0 1
======== CHANNEL f1 ========
NUC1 1H
P1 7.60 usec
PL1 -1.00 dB
SFO1 600.1337060 MHz
F2 - Processing parameters
SI 32768
SF 600.1299958 MHz
WDW EM
SSB 0
LB 0.30 Hz
GB 0
PC 1.00
ppm
0.05 1.00 0.99 1.01 1.06 3.04 3.99 0.99 1.02 3.03 2.14 2.17 3.28 3.58

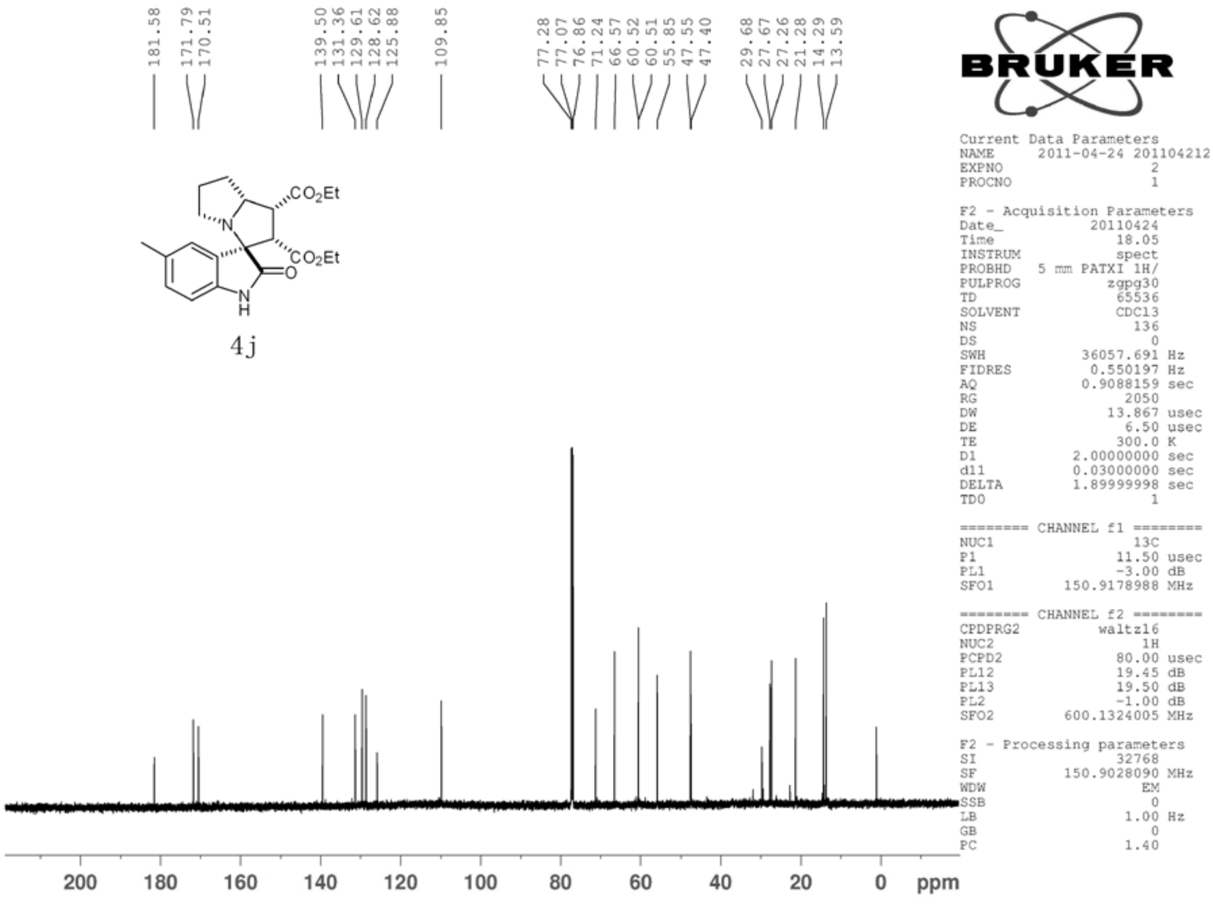
181.58
171.79
170.51
139.50
131.36
129.61
128.62
125.88
109.85
77.28
77.07
76.86
71.24
66.57
60.52
60.51
55.85
47.55
47.40
29.68
27.67
27.26
21.28
14.29
13.59
CO2Et
CO2Et
N
O
N
H
4j
BRUKER
Current Data Parameters
NAME 2011-04-24 201104212
EXPNO 2
PROCNO 1
F2 - Acquisition Parameters
Date_ 20110424
Time 18.05
INSTRUM spect
PROBHD 5 mm PATXI 1H/
PULPROG zgpg30
TD 65536
SOLVENT CDCl3
NS 136
DS 0
SWH 36057.691 Hz
FIDRES 0.550197 Hz
AQ 0.9088159 sec
RG 2050
DW 13.867 usec
DE 6.50 usec
TE 300.0 K
D1 2.00000000 sec
d11 0.03000000 sec
DELTA 1.89999998 sec
TD0 1
======== CHANNEL f1 ========
NUC1 13C
P1 11.50 usec
PL1 -3.00 dB
SFO1 150.9178988 MHz
======== CHANNEL f2 ========
CPDPRG2 waltz16
NUC2 1H
PCPD2 80.00 usec
PL12 19.45 dB
PL13 19.50 dB
PL2 -1.00 dB
SFO2 600.1324005 MHz
F2 - Processing parameters
SI 32768
SF 150.9028090 MHz
WDW EM
SSB 0
LB 1.00 Hz
GB 0
PC 1.40
200 180 160 140 120 100 80 60 40 20 0 ppm

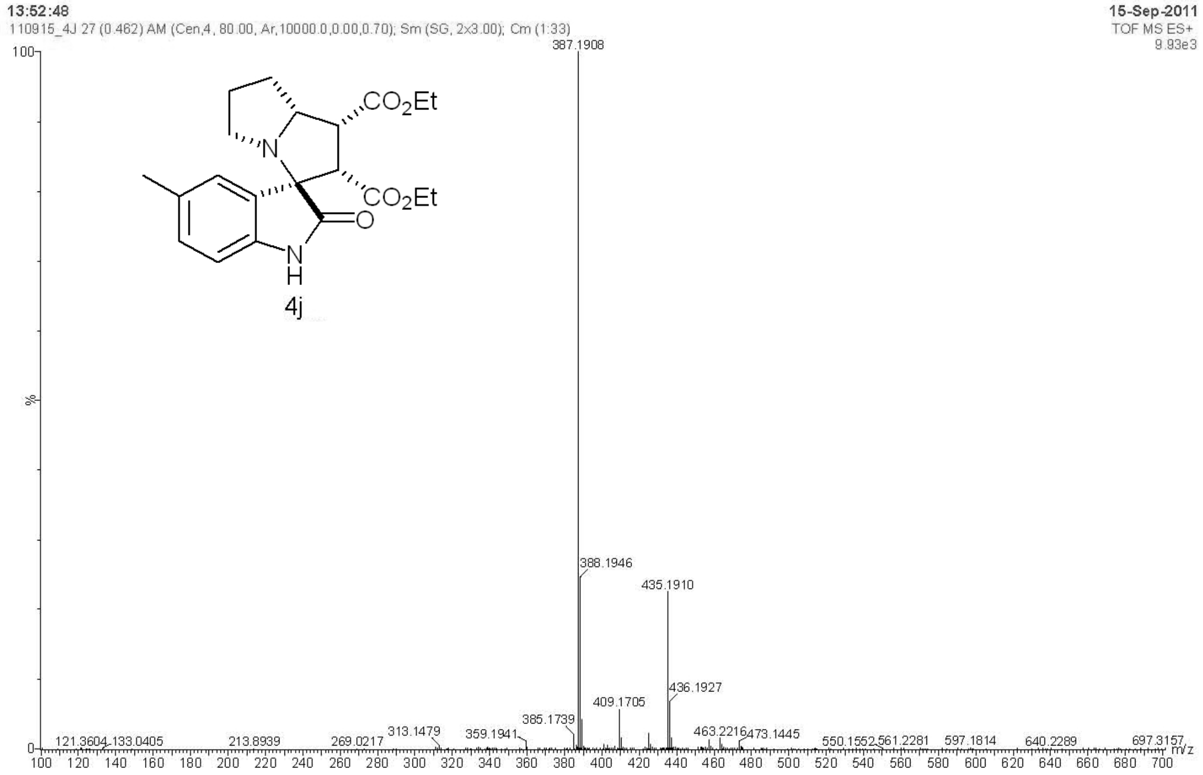
13:52:48
110915_4J 27 (0.462) AM (Cen,4, 80.00, Ar,10000.0,0.00,0.70); Sm (SG, 2x3.00); Cm (1:33)
15-Sep-2011
TOF MS ES+
9.93e3
100
%
0
CO2Et
CO2Et
N
O
N
H
4j
387.1908
388.1946
435.1910
436.1927
409.1705
385.1739
359.1941
313.1479
269.0217
213.8939
121.3604
133.0405
463.2216
473.1445
550.1552
561.2281
597.1814
640.2289
697.3157
m/z
100 120 140 160 180 200 220 240 260 280 300 320 340 360 380 400 420 440 460 480 500 520 540 560 580 600 620 640 660 680 700

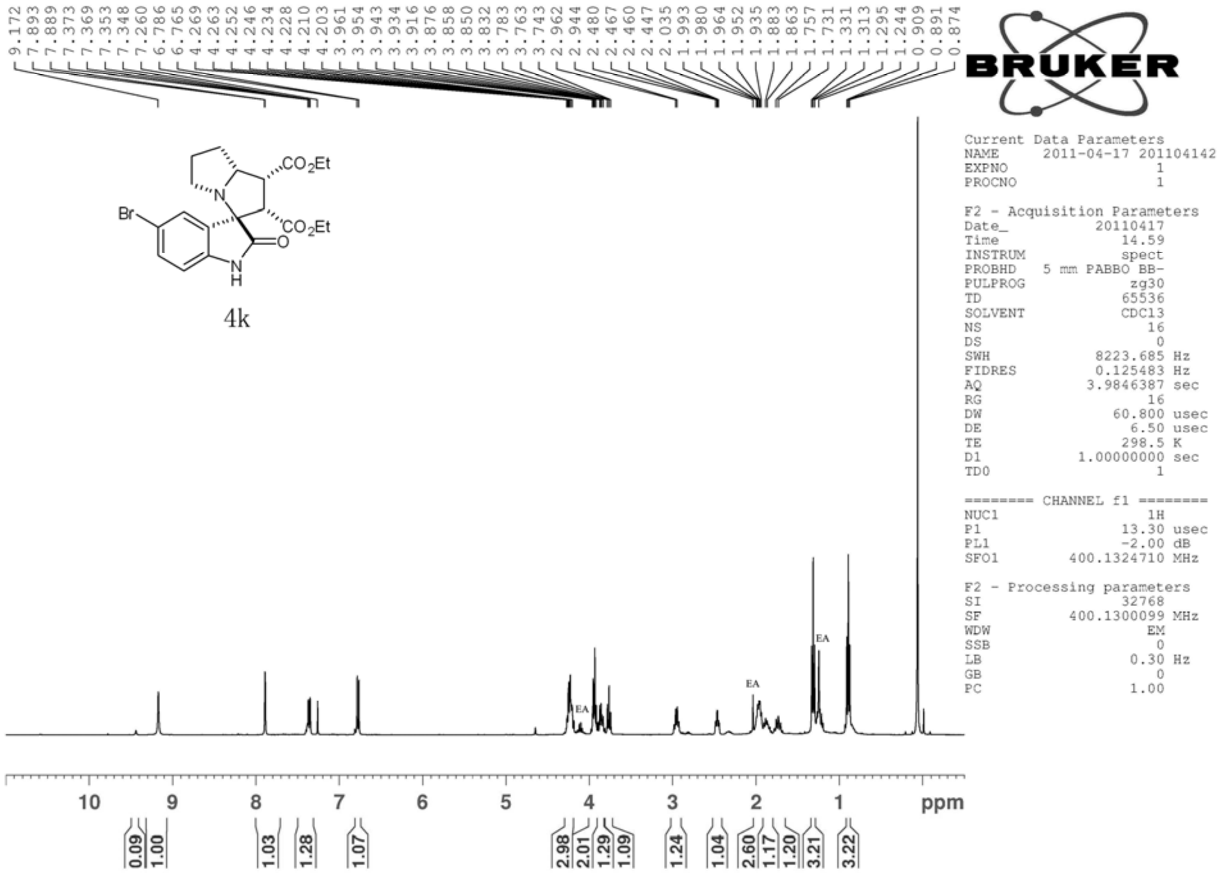
9.172 7.893 7.889 7.373 7.369 7.353 7.348 7.260 6.786 6.765 4.269 4.263 4.252 4.246 4.234 4.228 4.210 4.203 3.961 3.954 3.943 3.934 3.916 3.876 3.858 3.850 3.832 3.783 3.763 3.743 2.962 2.944 2.480 2.467 2.460 2.447 2.035 1.993 1.980 1.964 1.952 1.935 1.883 1.863 1.757 1.731 1.331 1.313 1.295 1.244 0.909 0.891 0.874
BRUKER
CO2Et
Br
N
CO2Et
O
N
H
4k
EA
EA
EA
Current Data Parameters
NAME 2011-04-17 201104142
EXPNO 1
PROCNO 1
F2 - Acquisition Parameters
Date_ 20110417
Time 14.59
INSTRUM spect
PROBHD 5 mm PABBO BB-
PULPROG zg30
TD 65536
SOLVENT CDCl3
NS 16
DS 0
SWH 8223.685 Hz
FIDRES 0.125483 Hz
AQ 3.9846387 sec
RG 16
DW 60.800 usec
DE 6.50 usec
TE 298.5 K
D1 1.00000000 sec
TD0 1
======== CHANNEL f1 ========
NUC1 1H
P1 13.30 usec
PL1 -2.00 dB
SFO1 400.1324710 MHz
F2 - Processing parameters
SI 32768
SF 400.1300099 MHz
WDW EM
SSB 0
LB 0.30 Hz
GB 0
PC 1.00
10 9 8 7 6 5 4 3 2 1 ppm
0.09 1.00 1.03 1.28 1.07 2.98 2.01 1.29 1.09 1.24 1.04 2.60 1.17 1.20 3.21 3.22

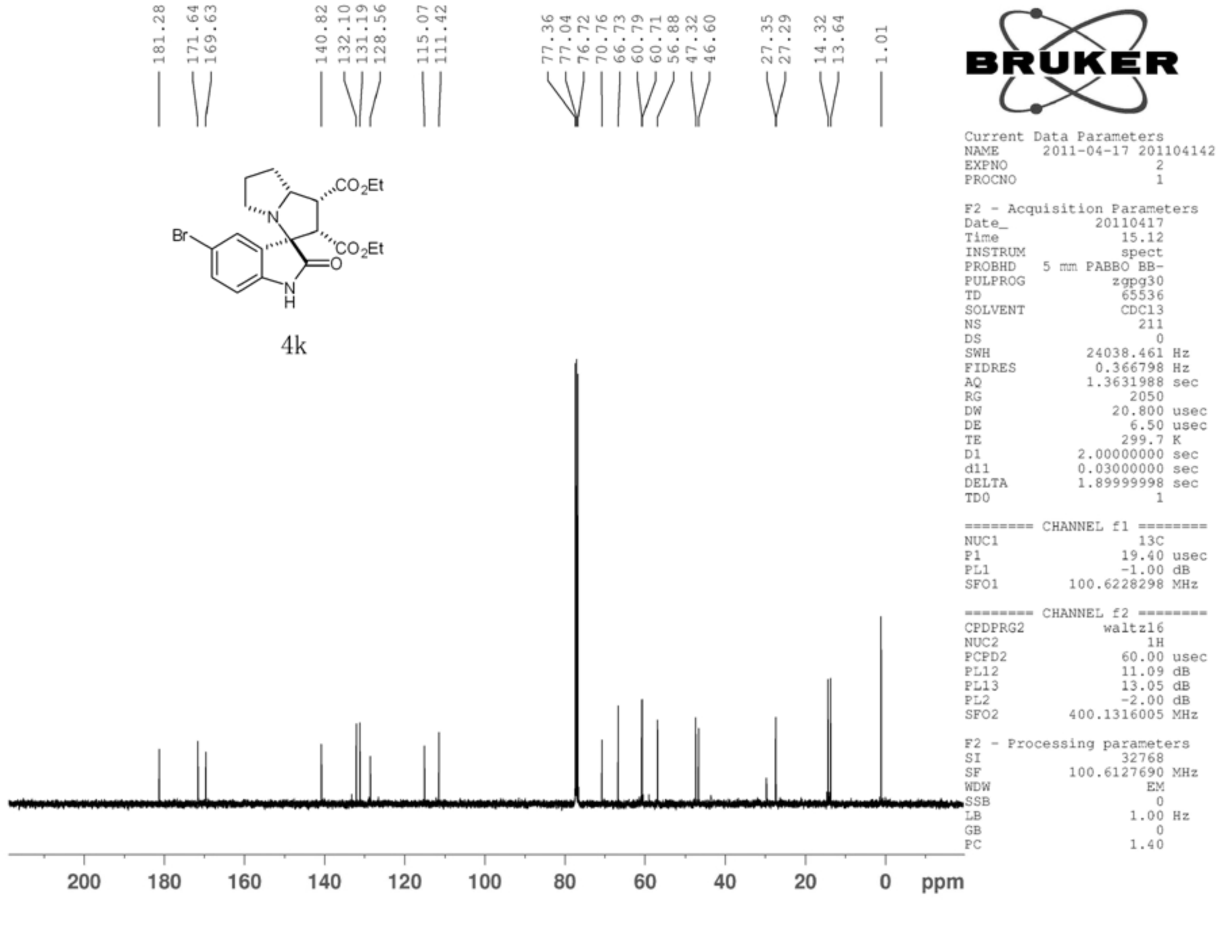
181.28 171.64 169.63 140.82 132.10 131.19 128.56 115.07 111.42 77.36 77.04 76.72 70.76 66.73 60.79 60.71 56.88 47.32 46.60 27.35 27.29 14.32 13.64 1.01
BRUKER
CO2Et
Br
N
CO2Et
O
N
H
4k
Current Data Parameters
NAME 2011-04-17 201104142
EXPNO 2
PROCNO 1
F2 - Acquisition Parameters
Date_ 20110417
Time 15.12
INSTRUM spect
PROBHD 5 mm PABBO BB-
PULPROG zgpg30
TD 65536
SOLVENT CDCl3
NS 211
DS 0
SWH 24038.461 Hz
FIDRES 0.366798 Hz
AQ 1.3631988 sec
RG 2050
DW 20.800 usec
DE 6.50 usec
TE 299.7 K
D1 2.00000000 sec
d11 0.03000000 sec
DELTA 1.89999998 sec
TD0 1
======== CHANNEL f1 ========
NUC1 13C
P1 19.40 usec
PL1 -1.00 dB
SFO1 100.6228298 MHz
======== CHANNEL f2 ========
CPDPRG2 waltz16
NUC2 1H
PCPD2 60.00 usec
PL12 11.09 dB
PL13 13.05 dB
PL2 -2.00 dB
SFO2 400.1316005 MHz
F2 - Processing parameters
SI 32768
SF 100.6127690 MHz
WDW EM
SSB 0
LB 1.00 Hz
GB 0
PC 1.40
200 180 160 140 120 100 80 60 40 20 0 ppm

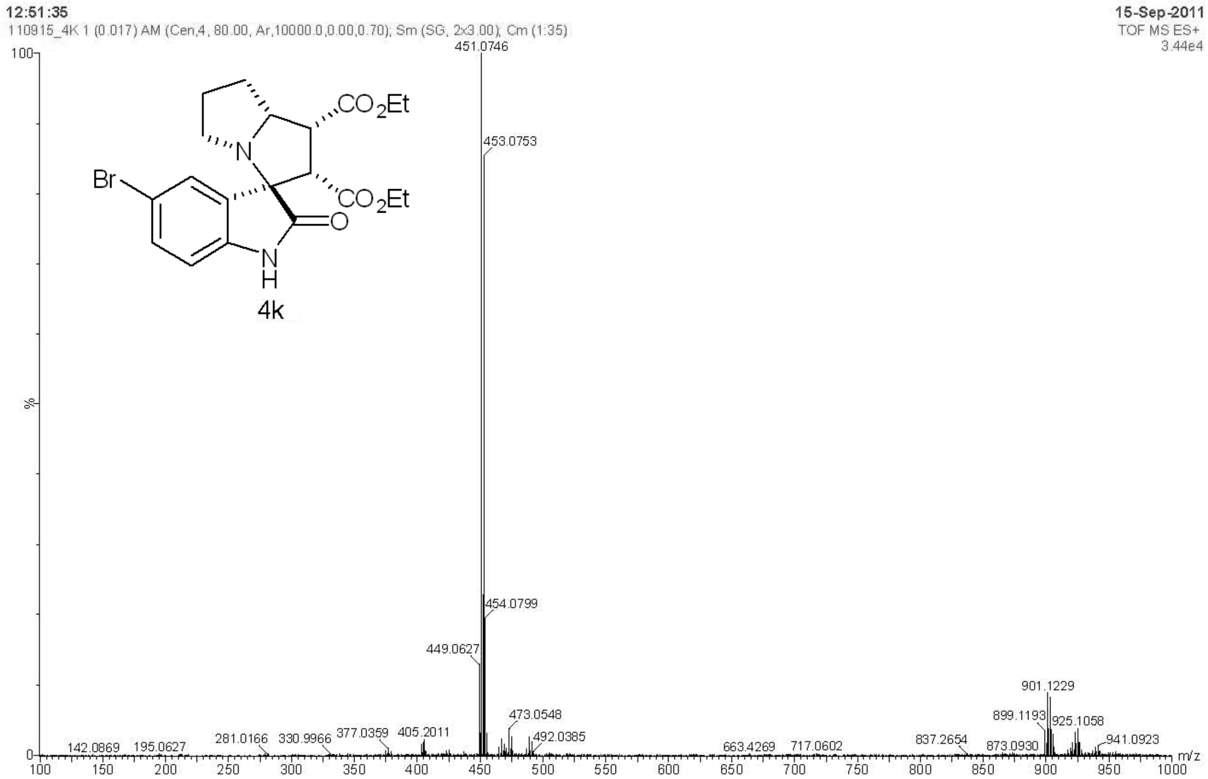
12:51:35
110915_4K 1 (0.017) AM (Cen,4, 80.00, Ar,10000.0,0.00,0.70); Sm (SG, 2x3.00); Cm (1:35)
15-Sep-2011
TOF MS ES+
3.44e4
Br
N
CO2Et
CO2Et
O
N
H
4k
451.0746
453.0753
454.0799
449.0627
473.0548
492.0385
405.2011
377.0359
330.9966
281.0166
195.0627
142.0869
663.4269
717.0602
837.2654
873.0930
899.1193
901.1229
925.1058
941.0923
%
m/z

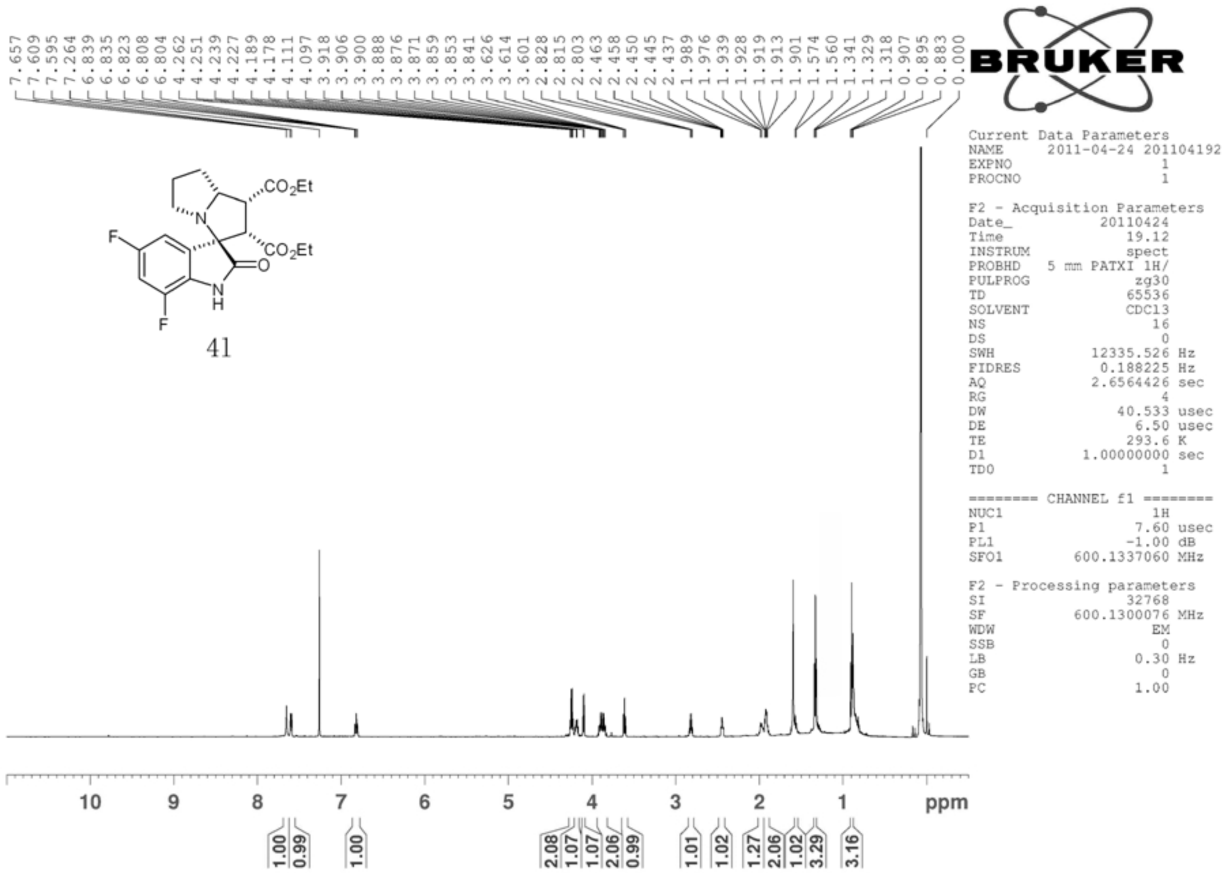
7.657
7.609
7.595
7.264
6.839
6.835
6.823
6.808
6.804
4.262
4.251
4.239
4.227
4.189
4.178
4.111
4.097
3.918
3.906
3.900
3.888
3.876
3.871
3.859
3.853
3.841
3.626
3.614
3.601
2.828
2.815
2.803
2.463
2.458
2.450
2.445
2.437
1.989
1.976
1.939
1.928
1.919
1.913
1.901
1.574
1.560
1.341
1.329
1.318
0.907
0.895
0.883
0.000
BRUKER
Current Data Parameters
NAME 2011-04-24 201104192
EXPNO 1
PROCNO 1
F2 - Acquisition Parameters
Date_ 20110424
Time 19.12
INSTRUM spect
PROBHD 5 mm PATXI 1H/
PULPROG zg30
TD 65536
SOLVENT CDCl3
NS 16
DS 0
SWH 12335.526 Hz
FIDRES 0.188225 Hz
AQ 2.6564426 sec
RG 4
DW 40.533 usec
DE 6.50 usec
TE 293.6 K
D1 1.00000000 sec
TD0 1
======== CHANNEL f1 ========
NUC1 1H
P1 7.60 usec
PL1 -1.00 dB
SFO1 600.1337060 MHz
F2 - Processing parameters
SI 32768
SF 600.1300076 MHz
WDW EM
SSB 0
LB 0.30 Hz
GB 0
PC 1.00
F
F
N
CO2Et
CO2Et
O
N
H
41
ppm
1.00
0.99
1.00
2.08
1.07
1.07
2.06
0.99
1.01
1.02
1.27
2.06
1.02
3.29
3.16

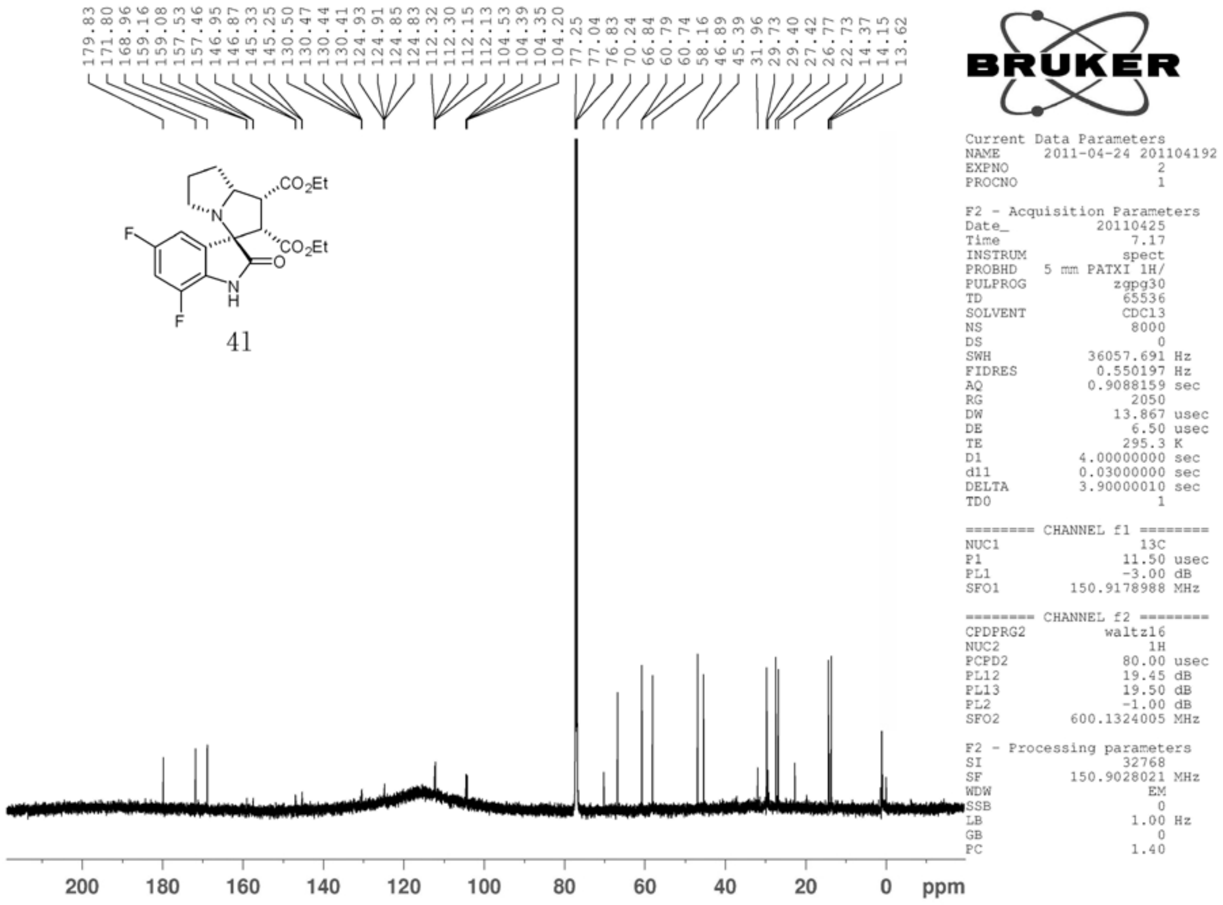
179.83
171.80
168.96
159.16
159.08
157.53
157.46
146.95
146.87
145.33
145.25
130.50
130.47
130.44
130.41
124.93
124.91
124.85
124.83
112.32
112.30
112.15
112.13
104.53
104.39
104.35
104.20
77.25
77.04
76.83
70.24
66.84
60.79
60.74
58.16
46.89
45.39
31.96
29.73
29.40
27.42
26.77
22.73
14.37
14.15
13.62
CO2Et
CO2Et
F
F
N
O
NH
41
BRUKER
Current Data Parameters
NAME 2011-04-24 201104192
EXPNO 2
PROCNO 1
F2 - Acquisition Parameters
Date_ 20110425
Time 7.17
INSTRUM spect
PROBHD 5 mm PATXI 1H/
PULPROG zgpg30
TD 65536
SOLVENT CDCl3
NS 8000
DS 0
SWH 36057.691 Hz
FIDRES 0.550197 Hz
AQ 0.9088159 sec
RG 2050
DW 13.867 usec
DE 6.50 usec
TE 295.3 K
D1 4.00000000 sec
d11 0.03000000 sec
DELTA 3.90000010 sec
TD0 1
======== CHANNEL f1 ========
NUC1 13C
P1 11.50 usec
PL1 -3.00 dB
SFO1 150.9178988 MHz
======== CHANNEL f2 ========
CPDPRG2 waltz16
NUC2 1H
PCPD2 80.00 usec
PL12 19.45 dB
PL13 19.50 dB
PL2 -1.00 dB
SFO2 600.1324005 MHz
F2 - Processing parameters
SI 32768
SF 150.9028021 MHz
WDW EM
SSB 0
LB 1.00 Hz
GB 0
PC 1.40
200
180
160
140
120
100
80
60
40
20
0
ppm

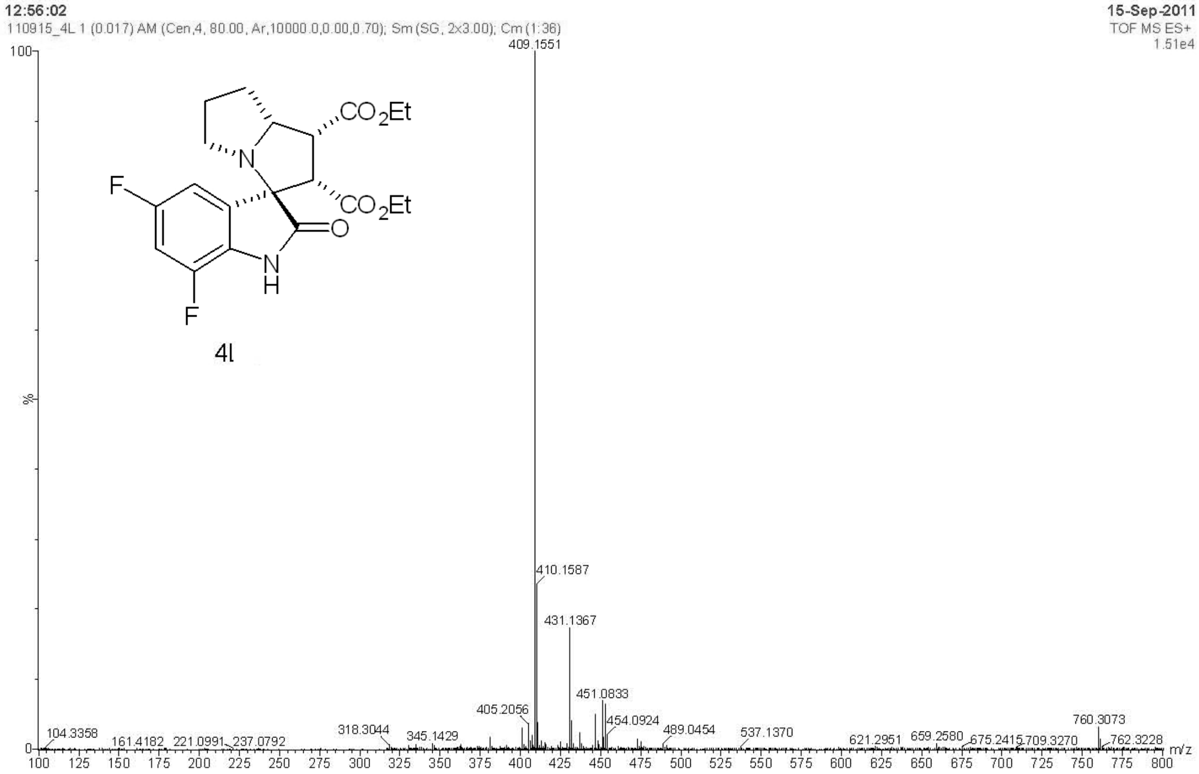
12:56:02
110915_4L 1 (0.017) AM (Cen,4, 80.00, Ar,10000.0,0.00,0.70); Sm (SG, 2x3.00); Cm (1:36)
15-Sep-2011
TOF MS ES+
1.51e4
100
%
0
409.1551
410.1587
431.1367
451.0833
454.0924
405.2056
489.0454
537.1370
104.3358
161.4182
221.0991
237.0792
318.3044
345.1429
621.2951
659.2580
675.2415
709.3270
760.3073
762.3228
m/z
CO2Et
CO2Et
F
F
N
O
NH
4l
100 125 150 175 200 225 250 275 300 325 350 375 400 425 450 475 500 525 550 575 600 625 650 675 700 725 750 775 800

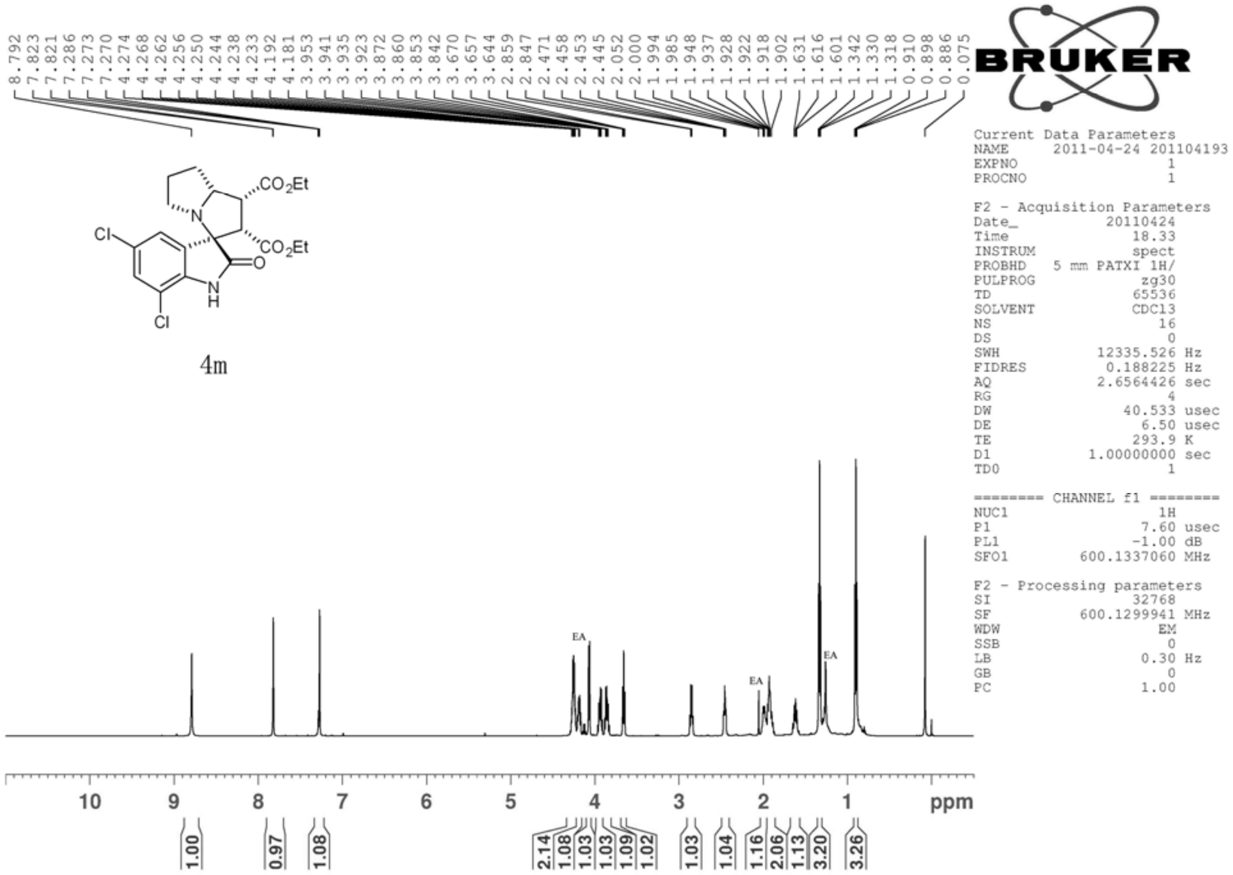
8.792 7.823 7.821 7.286 7.273 7.270 4.274 4.268 4.262 4.256 4.250 4.244 4.238 4.233 4.192 4.181 3.953 3.941 3.935 3.923 3.872 3.860 3.853 3.842 3.670 3.657 3.644 2.859 2.847 2.471 2.458 2.453 2.445 2.052 2.000 1.994 1.985 1.948 1.937 1.928 1.922 1.918 1.902 1.631 1.616 1.601 1.342 1.330 1.318 0.910 0.898 0.886 0.075
BRUKER
CO2Et
CO2Et
Cl
N
O
NH
H
Cl
4m
Current Data Parameters
NAME 2011-04-24 201104193
EXPNO 1
PROCNO 1
F2 - Acquisition Parameters
Date_ 20110424
Time 18.33
INSTRUM spect
PROBHD 5 mm PATXI 1H/
PULPROG zg30
TD 65536
SOLVENT CDCl3
NS 16
DS 0
SWH 12335.526 Hz
FIDRES 0.188225 Hz
AQ 2.6564426 sec
RG 4
DW 40.533 usec
DE 6.50 usec
TE 293.9 K
D1 1.00000000 sec
TD0 1
======== CHANNEL f1 ========
NUC1 1H
P1 7.60 usec
PL1 -1.00 dB
SFO1 600.1337060 MHz
F2 - Processing parameters
SI 32768
SF 600.1299941 MHz
WDW EM
SSB 0
LB 0.30 Hz
GB 0
PC 1.00
EA
EA
EA
10 9 8 7 6 5 4 3 2 1 ppm
1.00 0.97 1.08 2.14 1.08 1.03 1.03 1.09 1.02 1.03 1.04 1.16 2.06 1.13 3.20 3.26

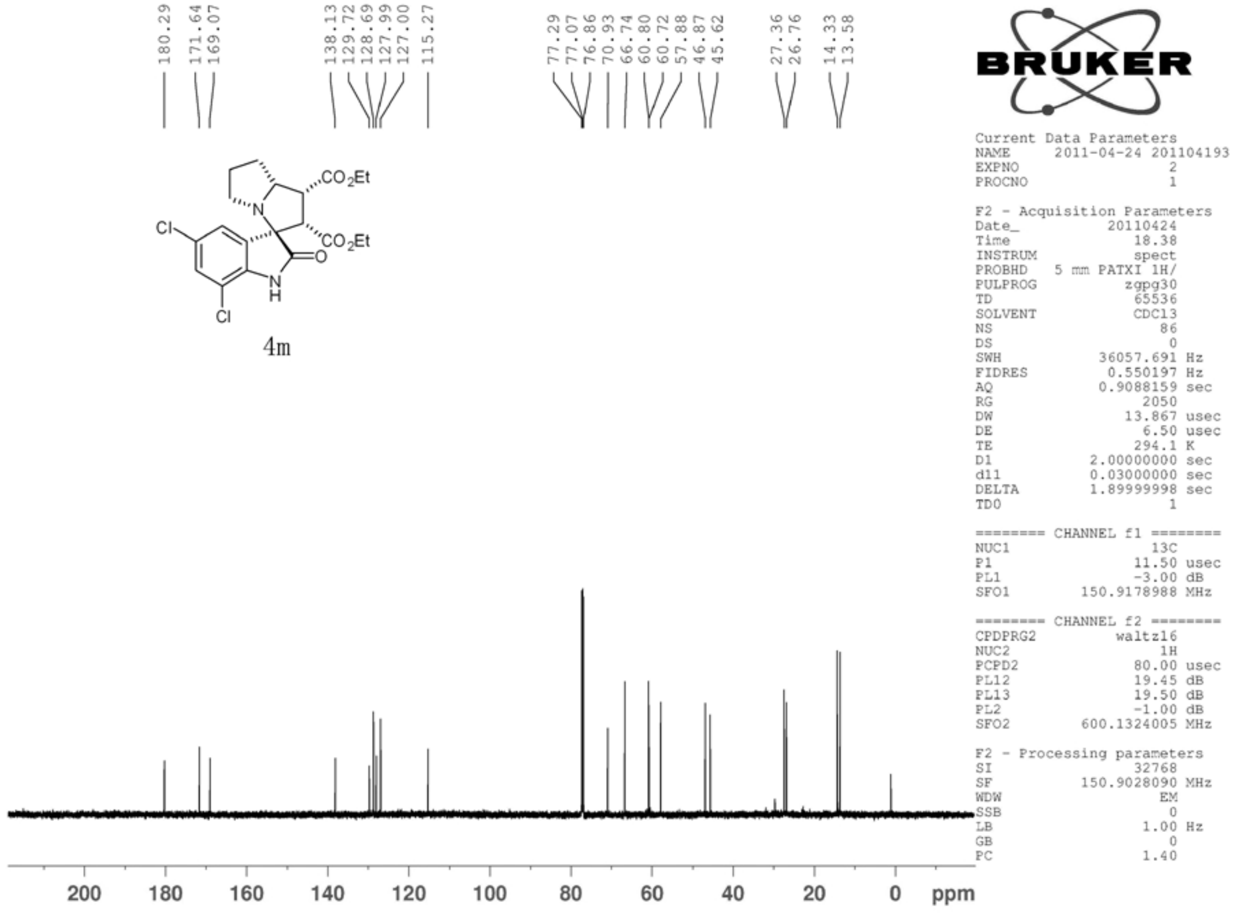
180.29 171.64 169.07 138.13 129.72 128.69 127.99 127.00 115.27 77.29 77.07 76.86 70.93 66.74 60.80 60.72 57.88 46.87 45.62 27.36 26.76 14.33 13.58
BRUKER
CO2Et
CO2Et
Cl
N
O
NH
H
Cl
4m
Current Data Parameters
NAME 2011-04-24 201104193
EXPNO 2
PROCNO 1
F2 - Acquisition Parameters
Date_ 20110424
Time 18.38
INSTRUM spect
PROBHD 5 mm PATXI 1H/
PULPROG zgpg30
TD 65536
SOLVENT CDCl3
NS 86
DS 0
SWH 36057.691 Hz
FIDRES 0.550197 Hz
AQ 0.9088159 sec
RG 2050
DW 13.867 usec
DE 6.50 usec
TE 294.1 K
D1 2.00000000 sec
d11 0.03000000 sec
DELTA 1.89999998 sec
TD0 1
======== CHANNEL f1 ========
NUC1 13C
P1 11.50 usec
PL1 -3.00 dB
SFO1 150.9178988 MHz
======== CHANNEL f2 ========
CPDPRG2 waltz16
NUC2 1H
PCPD2 80.00 usec
PL12 19.45 dB
PL13 19.50 dB
PL2 -1.00 dB
SFO2 600.1324005 MHz
F2 - Processing parameters
SI 32768
SF 150.9028090 MHz
WDW EM
SSB 0
LB 1.00 Hz
GB 0
PC 1.40
200 180 160 140 120 100 80 60 40 20 0 ppm

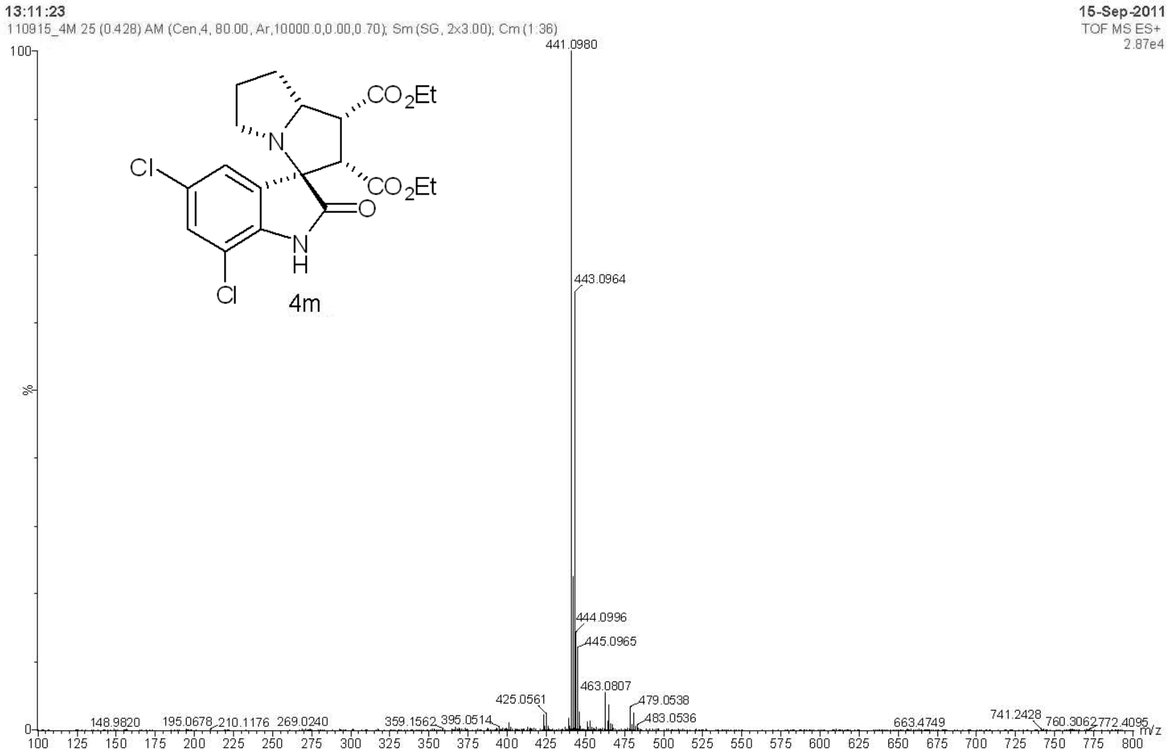
13:11:23
110915_4M 25 (0.428) AM (Cen,4, 80.00, Ar,10000.0,0.00,0.70); Sm (SG, 2x3.00); Cm (1:36)
15-Sep-2011
TOF MS ES+
2.87e4
441.0980
443.0964
444.0996
445.0965
463.0807
479.0538
483.0536
425.0561
4m
m/z

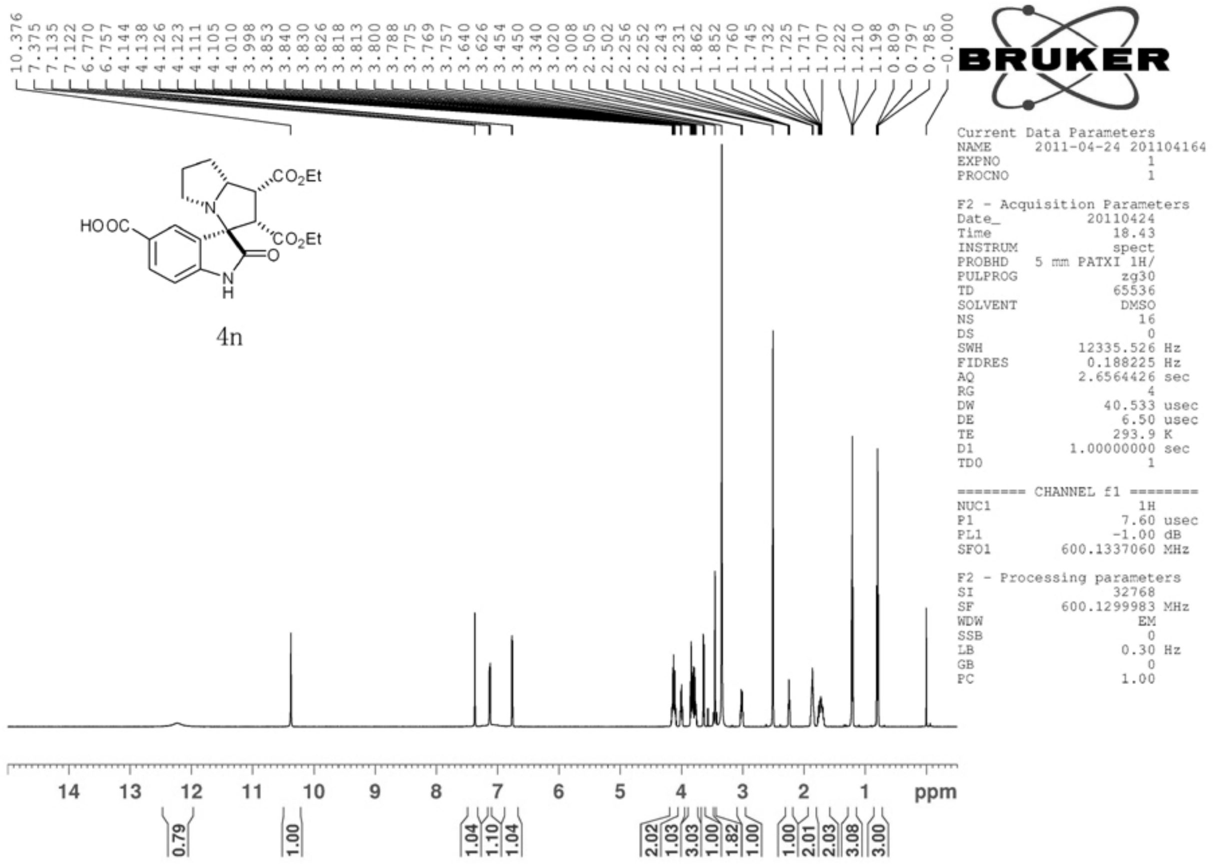
BRUKER
4n
Current Data Parameters
NAME 2011-04-24 201104164
EXPNO 1
PROCNO 1
F2 - Acquisition Parameters
Date_ 20110424
Time 18.43
INSTRUM spect
PROBHD 5 mm PATXI 1H/
PULPROG zg30
TD 65536
SOLVENT DMSO
NS 16
DS 0
SWH 12335.526 Hz
FIDRES 0.188225 Hz
AQ 2.6564426 sec
RG 4
DW 40.533 usec
DE 6.50 usec
TE 293.9 K
D1 1.00000000 sec
TD0 1
======== CHANNEL f1 ========
NUC1 1H
P1 7.60 usec
PL1 -1.00 dB
SFO1 600.1337060 MHz
F2 - Processing parameters
SI 32768
SF 600.1299983 MHz
WDW EM
SSB 0
LB 0.30 Hz
GB 0
PC 1.00
ppm

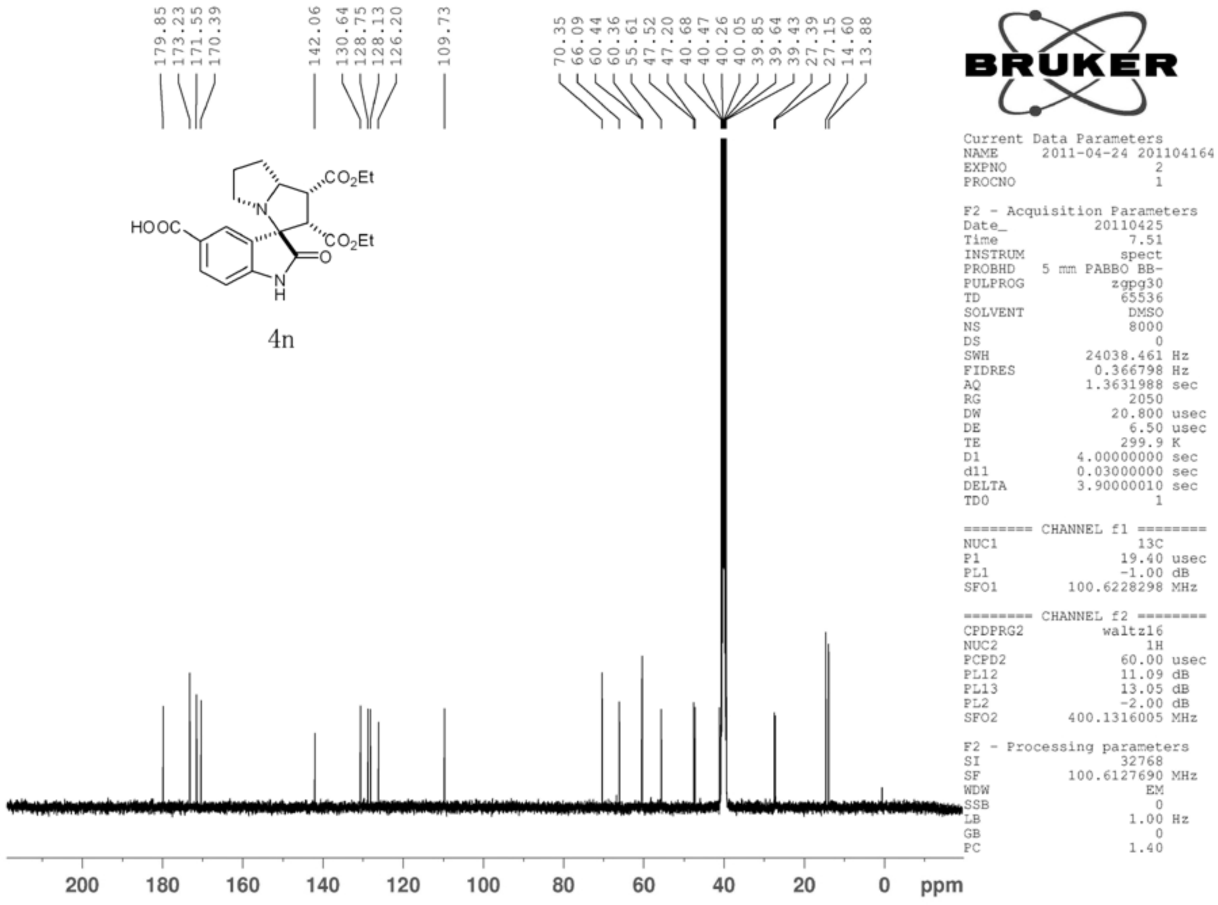
179.85
173.23
171.55
170.39
142.06
130.64
128.75
128.13
126.20
109.73
70.35
66.09
60.44
60.36
55.61
47.52
47.20
40.68
40.47
40.26
40.05
39.85
39.64
39.43
27.39
27.15
14.60
13.88
HOOC
CO2Et
CO2Et
N
O
N
H
4n
BRUKER
Current Data Parameters
NAME 2011-04-24 201104164
EXPNO 2
PROCNO 1
F2 - Acquisition Parameters
Date_ 20110425
Time 7.51
INSTRUM spect
PROBHD 5 mm PABBO BB-
PULPROG zgpg30
TD 65536
SOLVENT DMSO
NS 8000
DS 0
SWH 24038.461 Hz
FIDRES 0.366798 Hz
AQ 1.3631988 sec
RG 2050
DW 20.800 usec
DE 6.50 usec
TE 299.9 K
D1 4.00000000 sec
d11 0.03000000 sec
DELTA 3.90000010 sec
TD0 1
======== CHANNEL f1 ========
NUC1 13C
P1 19.40 usec
PL1 -1.00 dB
SFO1 100.6228298 MHz
======== CHANNEL f2 ========
CPDPRG2 waltz16
NUC2 1H
PCPD2 60.00 usec
PL12 11.09 dB
PL13 13.05 dB
PL2 -2.00 dB
SFO2 400.1316005 MHz
F2 - Processing parameters
SI 32768
SF 100.6127690 MHz
WDW EM
SSB 0
LB 1.00 Hz
GB 0
PC 1.40
200 180 160 140 120 100 80 60 40 20 0 ppm

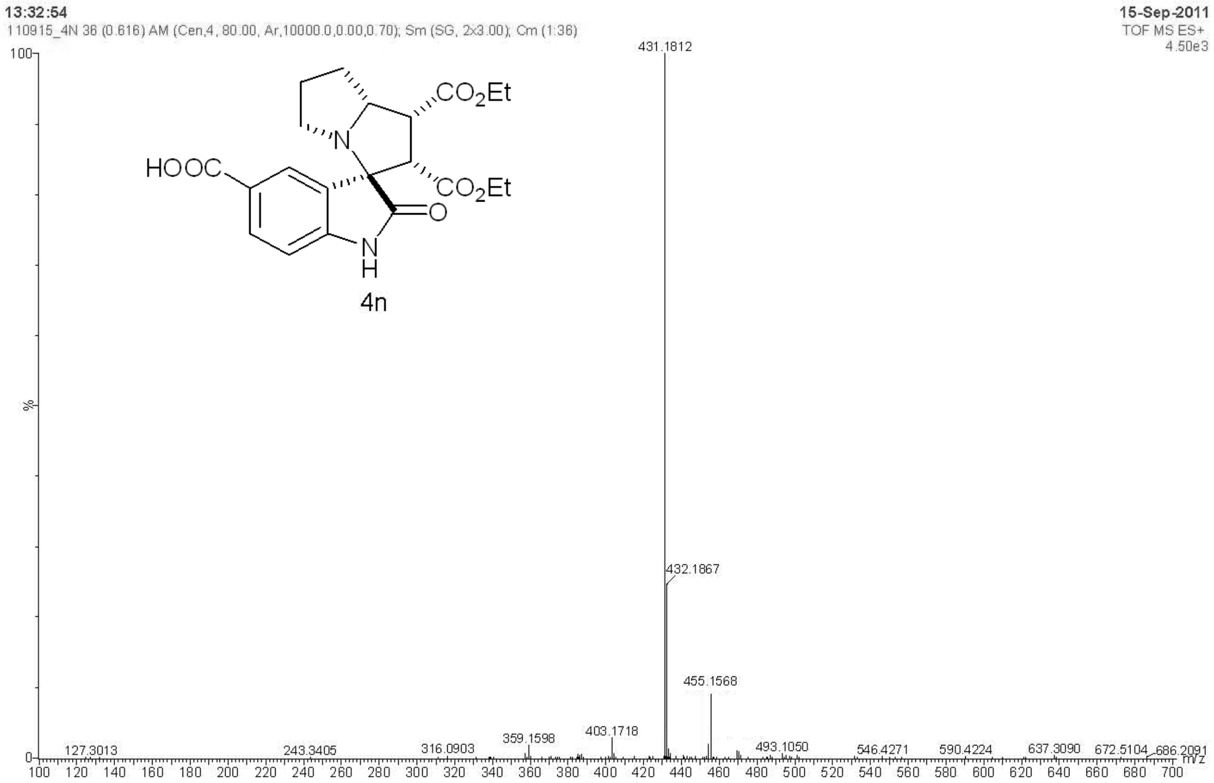
13:32:54
110915_4N 36 (0.616) AM (Cen,4, 80.00, Ar,10000.0,0.00,0.70); Sm (SG, 2x3.00); Cm (1:36)
15-Sep-2011
TOF MS ES+
4.50e3
100
%
0
HOOC
CO2Et
CO2Et
N
O
N
H
4n
431.1812
432.1867
455.1568
403.1718
359.1598
316.0903
243.3405
127.3013
493.1050
546.4271
590.4224
637.3090
672.5104
686.2091
m/z
100 120 140 160 180 200 220 240 260 280 300 320 340 360 380 400 420 440 460 480 500 520 540 560 580 600 620 640 660 680 700

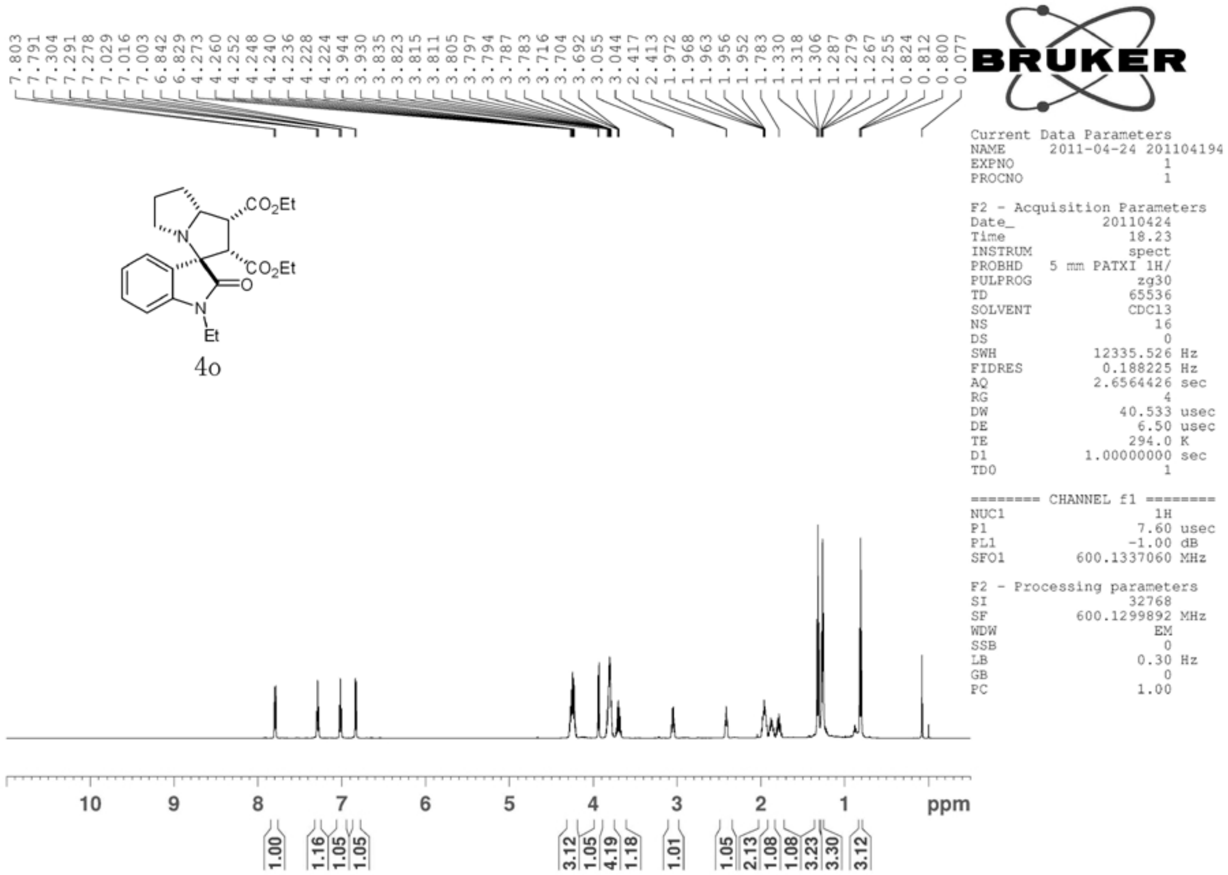
7.803
7.791
7.304
7.291
7.278
7.029
7.016
7.003
6.842
6.829
4.273
4.260
4.252
4.248
4.240
4.236
4.228
4.224
3.944
3.930
3.835
3.823
3.815
3.811
3.805
3.797
3.794
3.787
3.783
3.716
3.704
3.692
3.055
3.044
2.417
2.413
1.972
1.968
1.963
1.956
1.952
1.783
1.330
1.318
1.306
1.287
1.279
1.267
1.255
0.824
0.812
0.800
0.077
BRUKER
CO2Et
CO2Et
N
O
N
Et
4o
Current Data Parameters
NAME 2011-04-24 201104194
EXPNO 1
PROCNO 1
F2 - Acquisition Parameters
Date_ 20110424
Time 18.23
INSTRUM spect
PROBHD 5 mm PATXI 1H/
PULPROG zg30
TD 65536
SOLVENT CDCl3
NS 16
DS 0
SWH 12335.526 Hz
FIDRES 0.188225 Hz
AQ 2.6564426 sec
RG 4
DW 40.533 usec
DE 6.50 usec
TE 294.0 K
D1 1.00000000 sec
TD0 1
======== CHANNEL f1 ========
NUC1 1H
P1 7.60 usec
PL1 -1.00 dB
SFO1 600.1337060 MHz
F2 - Processing parameters
SI 32768
SF 600.1299892 MHz
WDW EM
SSB 0
LB 0.30 Hz
GB 0
PC 1.00
10 9 8 7 6 5 4 3 2 1 ppm
1.00
1.16
1.05
1.05
3.12
1.05
4.19
1.18
1.01
1.05
2.13
1.08
1.08
3.23
3.30
3.12

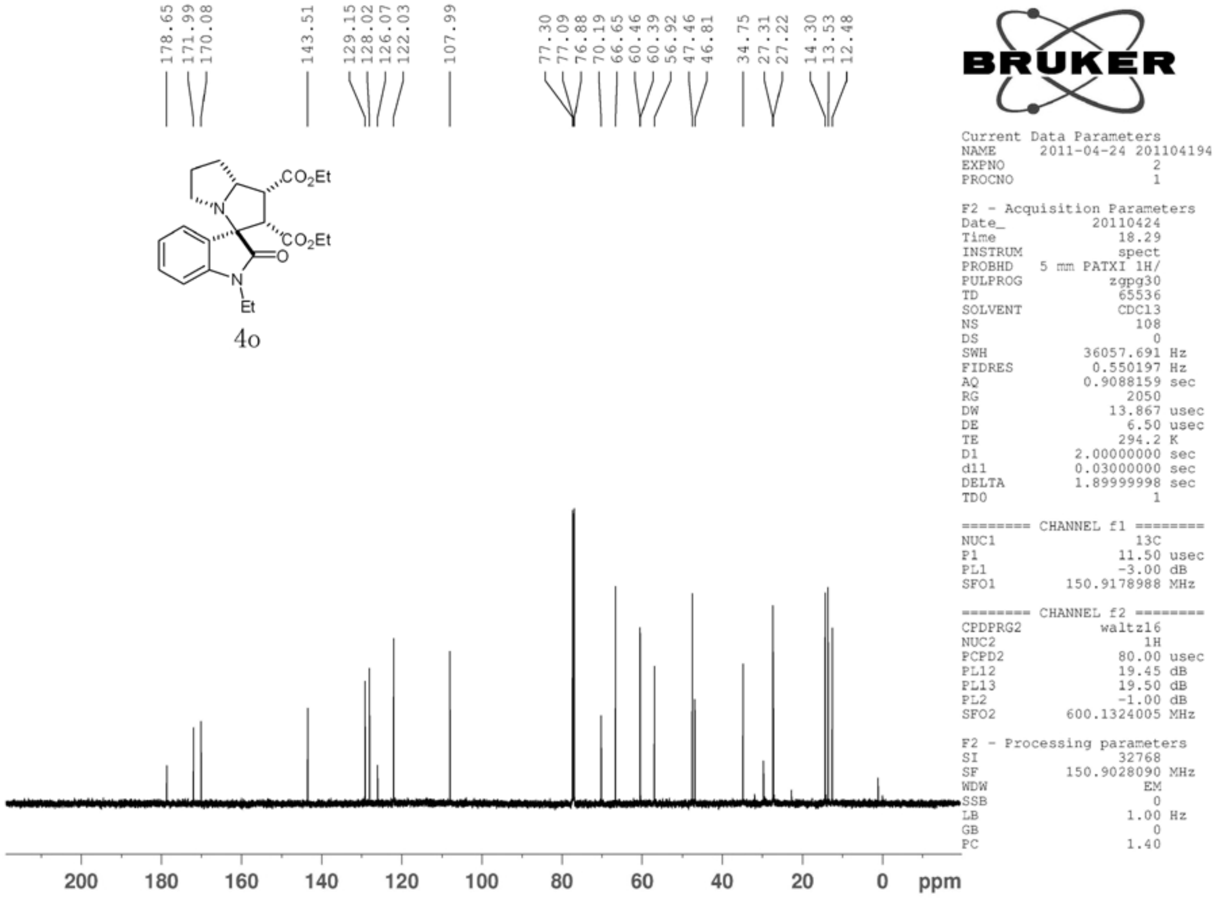
178.65
171.99
170.08
143.51
129.15
128.02
126.07
122.03
107.99
77.30
77.09
76.88
70.19
66.65
60.46
60.39
56.92
47.46
46.81
34.75
27.31
27.22
14.30
13.53
12.48
BRUKER
CO2Et
CO2Et
N
O
N
Et
4o
Current Data Parameters
NAME 2011-04-24 201104194
EXPNO 2
PROCNO 1
F2 - Acquisition Parameters
Date_ 20110424
Time 18.29
INSTRUM spect
PROBHD 5 mm PATXI 1H/
PULPROG zgpg30
TD 65536
SOLVENT CDCl3
NS 108
DS 0
SWH 36057.691 Hz
FIDRES 0.550197 Hz
AQ 0.9088159 sec
RG 2050
DW 13.867 usec
DE 6.50 usec
TE 294.2 K
D1 2.00000000 sec
d11 0.03000000 sec
DELTA 1.89999998 sec
TD0 1
======== CHANNEL f1 ========
NUC1 13C
P1 11.50 usec
PL1 -3.00 dB
SFO1 150.9178988 MHz
======== CHANNEL f2 ========
CPDPRG2 waltz16
NUC2 1H
PCPD2 80.00 usec
PL12 19.45 dB
PL13 19.50 dB
PL2 -1.00 dB
SFO2 600.1324005 MHz
F2 - Processing parameters
SI 32768
SF 150.9028090 MHz
WDW EM
SSB 0
LB 1.00 Hz
GB 0
PC 1.40
200 180 160 140 120 100 80 60 40 20 0 ppm

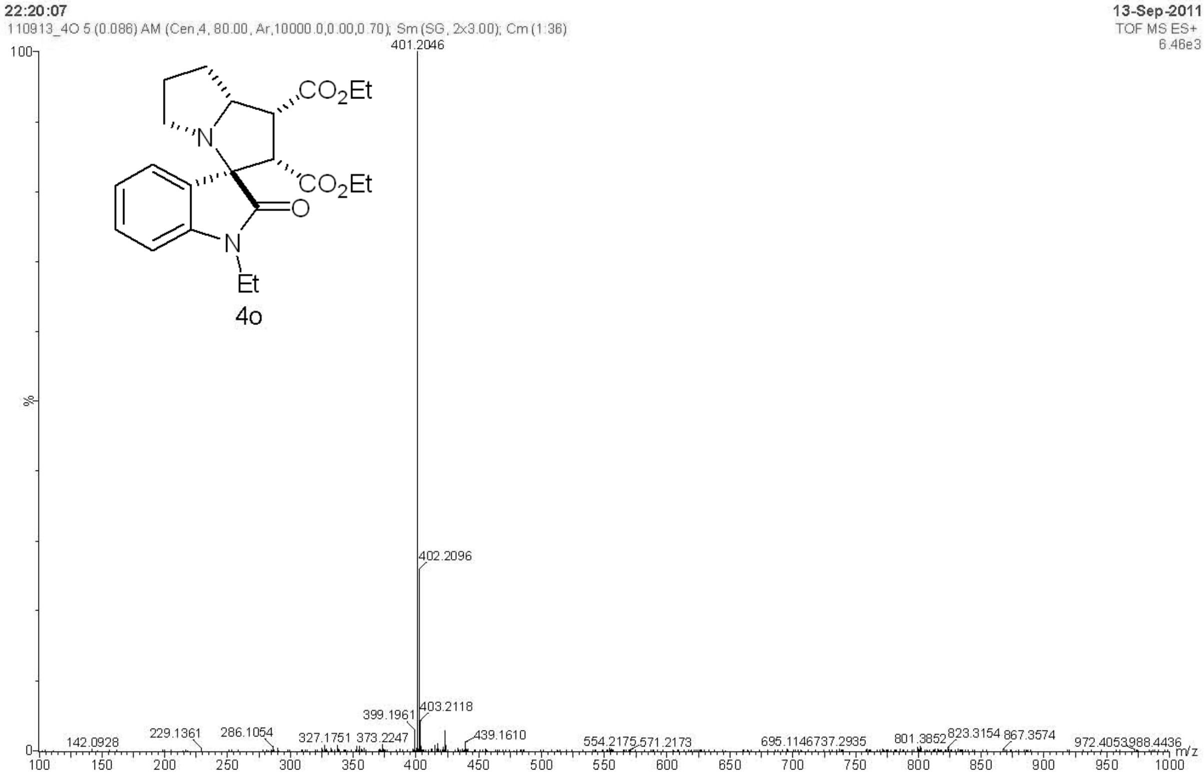
22:20:07
110913_4O 5 (0.086) AM (Cen,4, 80.00, Ar,10000.0,0.00,0.70); Sm (SG, 2x3.00); Cm (1:36)
13-Sep-2011
TOF MS ES+
6.46e3
%
401.2046
402.2096
403.2118
399.1961
142.0928
229.1361
286.1054
327.1751
373.2247
439.1610
554.2175
571.2173
695.1146
737.2935
801.3852
823.3154
867.3574
972.4053
988.4436
m/z
CO2Et
CO2Et
N
O
N
Et
4o

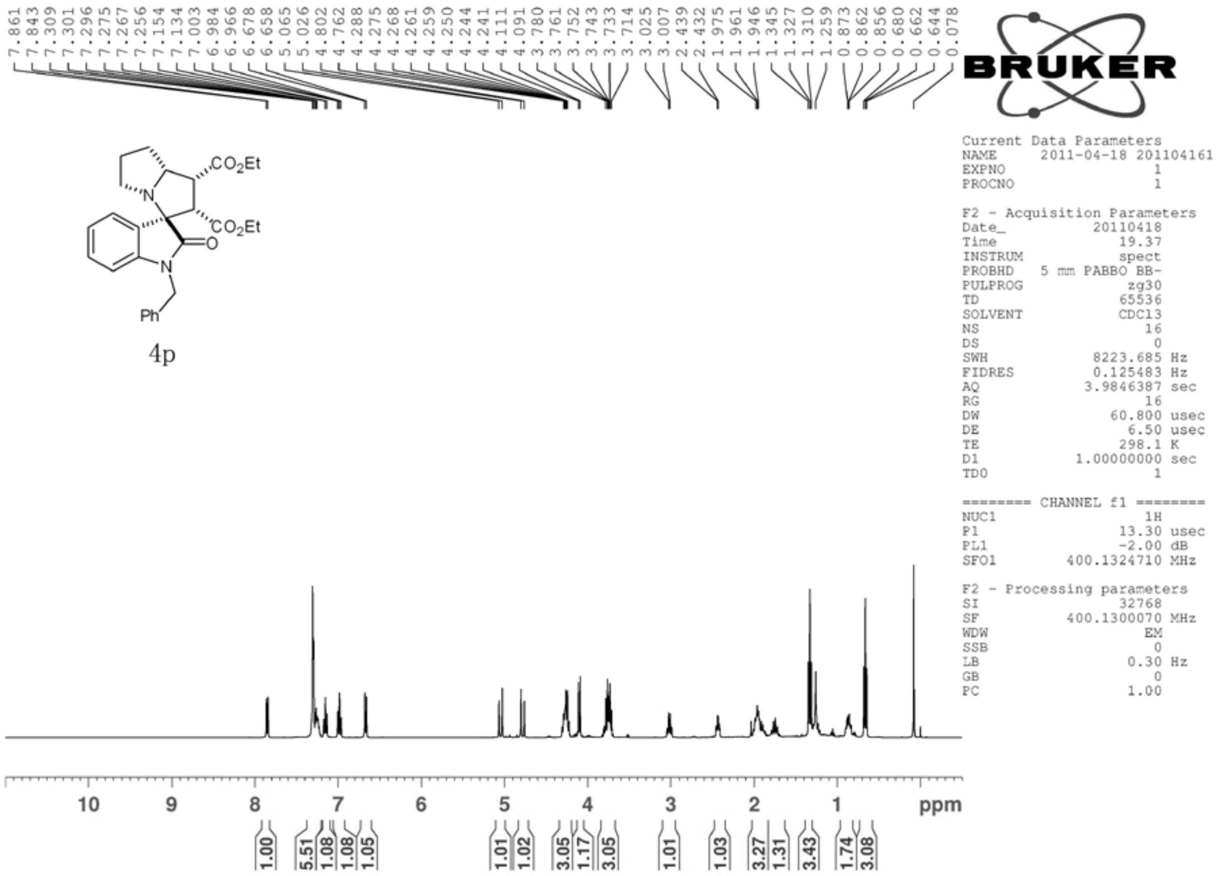
7.861
7.843
7.309
7.301
7.296
7.275
7.267
7.256
7.154
7.134
7.003
6.984
6.966
6.678
6.658
5.065
5.026
4.802
4.762
4.288
4.275
4.268
4.261
4.259
4.250
4.244
4.241
4.111
4.091
3.780
3.761
3.752
3.743
3.733
3.714
3.025
3.007
2.439
2.432
1.975
1.961
1.946
1.345
1.327
1.310
1.259
0.873
0.862
0.856
0.680
0.662
0.644
0.078
BRUKER
Current Data Parameters
NAME 2011-04-18 201104161
EXPNO 1
PROCNO 1
F2 - Acquisition Parameters
Date_ 20110418
Time 19.37
INSTRUM spect
PROBHD 5 mm PABBO BB-
PULPROG zg30
TD 65536
SOLVENT CDCl3
NS 16
DS 0
SWH 8223.685 Hz
FIDRES 0.125483 Hz
AQ 3.9846387 sec
RG 16
DW 60.800 usec
DE 6.50 usec
TE 298.1 K
D1 1.00000000 sec
TD0 1
======== CHANNEL f1 ========
NUC1 1H
P1 13.30 usec
PL1 -2.00 dB
SFO1 400.1324710 MHz
F2 - Processing parameters
SI 32768
SF 400.1300070 MHz
WDW EM
SSB 0
LB 0.30 Hz
GB 0
PC 1.00
ppm
1.00
5.51
1.08
1.08
1.05
1.01
1.02
3.05
1.17
3.05
1.01
1.03
3.27
1.31
3.43
1.74
3.08
CO2Et
CO2Et
N
O
N
Ph
4p

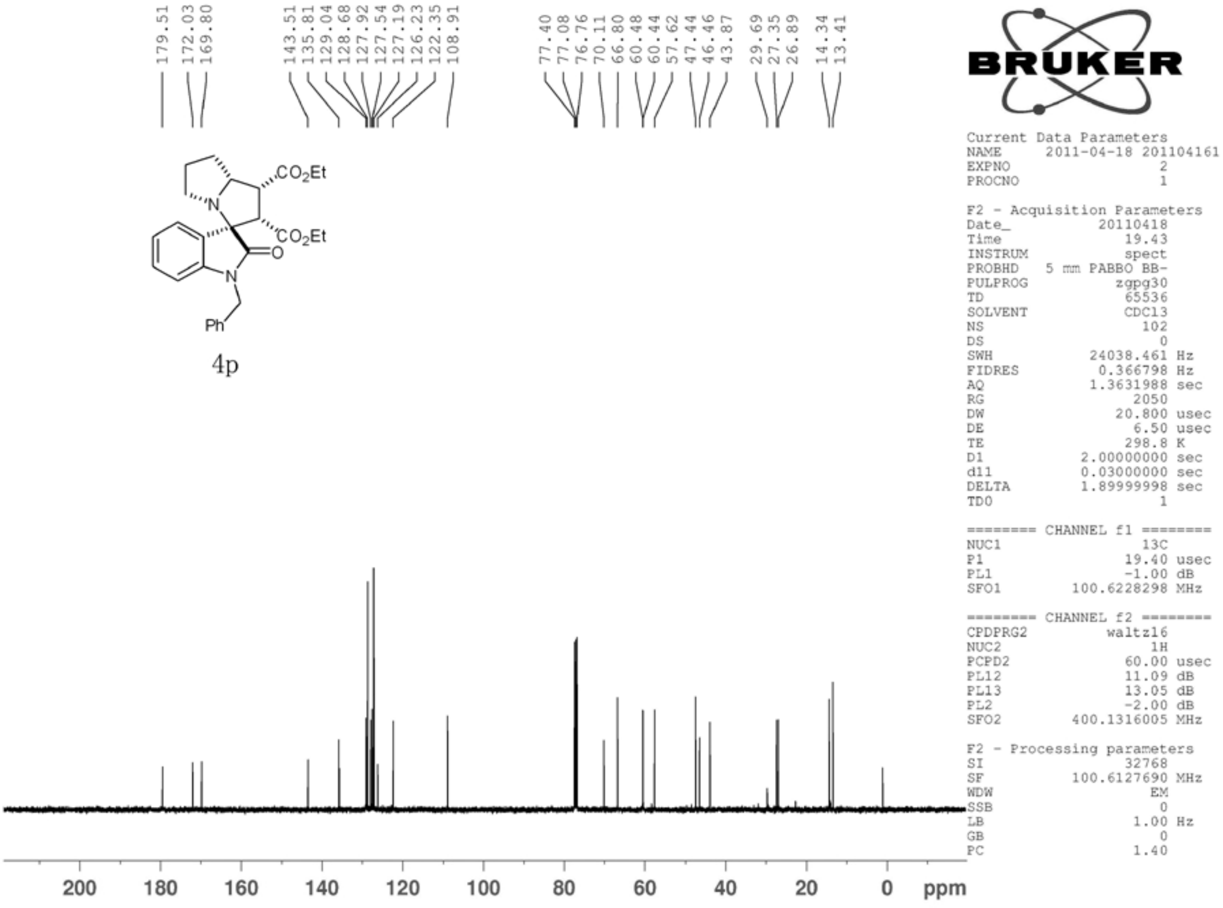
179.51
172.03
169.80
143.51
135.81
129.04
128.68
127.92
127.54
127.19
126.23
122.35
108.91
77.40
77.08
76.76
70.11
66.80
60.48
60.44
57.62
47.44
46.46
43.87
29.69
27.35
26.89
14.34
13.41
CO2Et
CO2Et
N
O
N
Ph
4p
BRUKER
Current Data Parameters
NAME 2011-04-18 201104161
EXPNO 2
PROCNO 1
F2 - Acquisition Parameters
Date_ 20110418
Time 19.43
INSTRUM spect
PROBHD 5 mm PABBO BB-
PULPROG zgpg30
TD 65536
SOLVENT CDCl3
NS 102
DS 0
SWH 24038.461 Hz
FIDRES 0.366798 Hz
AQ 1.3631988 sec
RG 2050
DW 20.800 usec
DE 6.50 usec
TE 298.8 K
D1 2.00000000 sec
d11 0.03000000 sec
DELTA 1.89999998 sec
TD0 1
======== CHANNEL f1 ========
NUC1 13C
P1 19.40 usec
PL1 -1.00 dB
SFO1 100.6228298 MHz
======== CHANNEL f2 ========
CPDPRG2 waltz16
NUC2 1H
PCPD2 60.00 usec
PL12 11.09 dB
PL13 13.05 dB
PL2 -2.00 dB
SFO2 400.1316005 MHz
F2 - Processing parameters
SI 32768
SF 100.6127690 MHz
WDW EM
SSB 0
LB 1.00 Hz
GB 0
PC 1.40
200 180 160 140 120 100 80 60 40 20 0 ppm

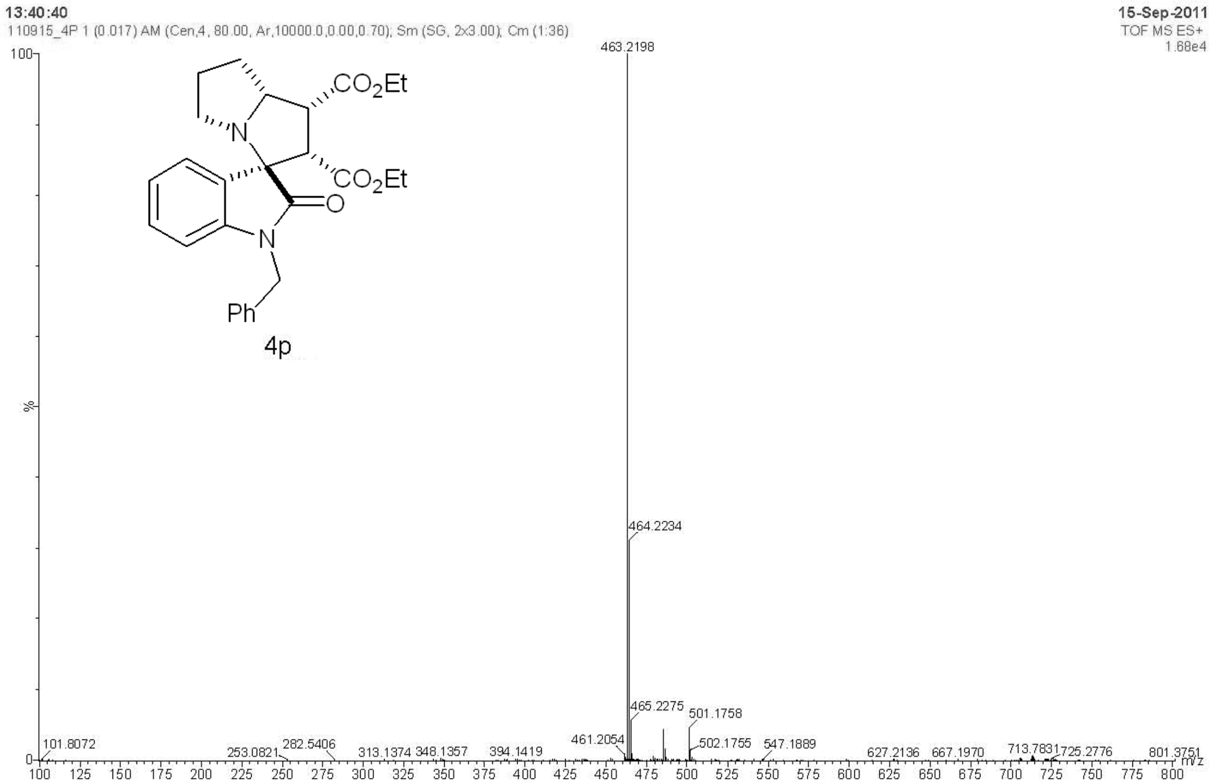
13:40:40
110915_4P 1 (0.017) AM (Cen,4, 80.00, Ar,10000.0,0.00,0.70); Sm (SG, 2x3.00); Cm (1:36)
15-Sep-2011
TOF MS ES+
1.88e4
100
%
0
CO2Et
CO2Et
N
O
N
Ph
4p
463.2198
464.2234
465.2275
501.1758
502.1755
547.1889
461.2054
101.8072
253.0821
282.5406
313.1374
348.1357
394.1419
627.2136
667.1970
713.7831
725.2776
801.3751
m/z
100 125 150 175 200 225 250 275 300 325 350 375 400 425 450 475 500 525 550 575 600 625 650 675 700 725 750 775 800

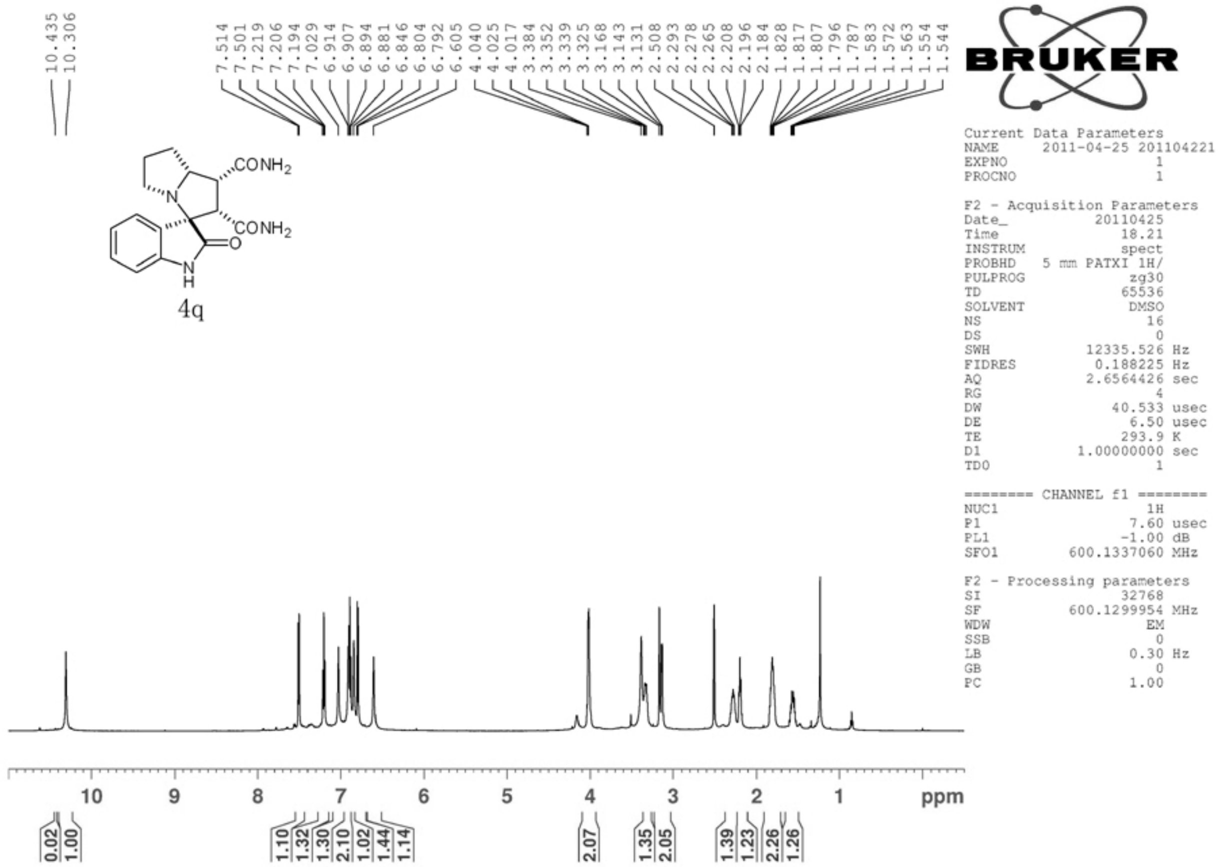
10.435
10.306
7.514
7.501
7.219
7.206
7.194
7.029
6.914
6.907
6.894
6.881
6.846
6.804
6.792
6.605
4.040
4.025
4.017
3.384
3.352
3.339
3.325
3.168
3.143
3.131
2.508
2.293
2.278
2.265
2.208
2.196
2.184
1.828
1.817
1.807
1.796
1.787
1.583
1.572
1.563
1.554
1.544
CONH2
CONH2
N
O
N
H
4q
BRUKER
Current Data Parameters
NAME 2011-04-25 201104221
EXPNO 1
PROCNO 1
F2 - Acquisition Parameters
Date_ 20110425
Time 18.21
INSTRUM spect
PROBHD 5 mm PATXI 1H/
PULPROG zg30
TD 65536
SOLVENT DMSO
NS 16
DS 0
SWH 12335.526 Hz
FIDRES 0.188225 Hz
AQ 2.6564426 sec
RG 4
DW 40.533 usec
DE 6.50 usec
TE 293.9 K
D1 1.00000000 sec
TD0 1
======== CHANNEL f1 ========
NUC1 1H
P1 7.60 usec
PL1 -1.00 dB
SFO1 600.1337060 MHz
F2 - Processing parameters
SI 32768
SF 600.1299954 MHz
WDW EM
SSB 0
LB 0.30 Hz
GB 0
PC 1.00
10
9
8
7
6
5
4
3
2
1
ppm
0.02
1.00
1.10
1.32
1.30
2.10
1.02
1.44
1.14
2.07
1.35
2.05
1.39
1.23
2.26
1.26

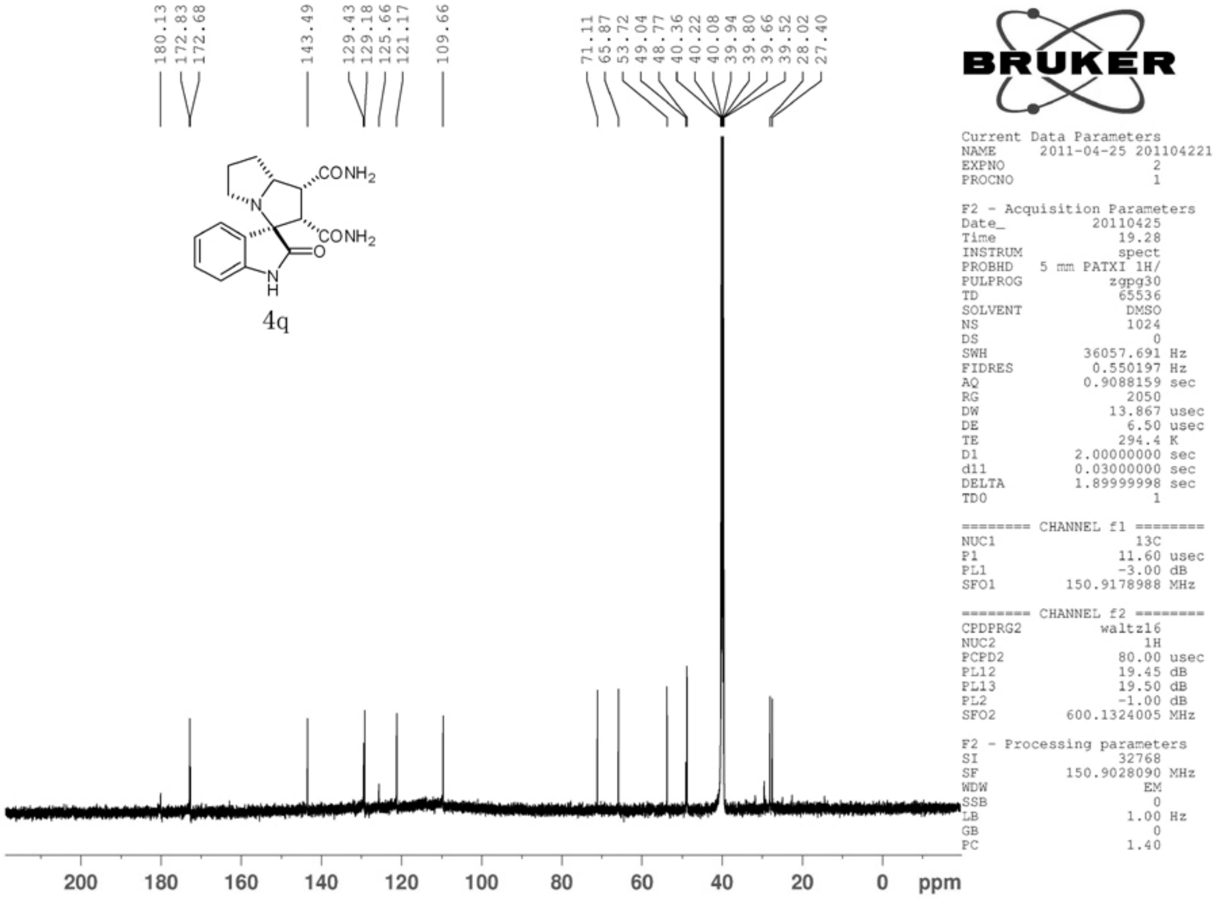
180.13
172.83
172.68
143.49
129.43
129.18
125.66
121.17
109.66
71.11
65.87
53.72
49.04
48.77
40.36
40.22
40.08
39.94
39.80
39.66
39.52
28.02
27.40
CONH2
CONH2
N
O
N
H
4q
BRUKER
Current Data Parameters
NAME 2011-04-25 201104221
EXPNO 2
PROCNO 1
F2 - Acquisition Parameters
Date_ 20110425
Time 19.28
INSTRUM spect
PROBHD 5 mm PATXI 1H/
PULPROG zgpg30
TD 65536
SOLVENT DMSO
NS 1024
DS 0
SWH 36057.691 Hz
FIDRES 0.550197 Hz
AQ 0.9088159 sec
RG 2050
DW 13.867 usec
DE 6.50 usec
TE 294.4 K
D1 2.00000000 sec
d11 0.03000000 sec
DELTA 1.89999998 sec
TD0 1
======== CHANNEL f1 ========
NUC1 13C
P1 11.60 usec
PL1 -3.00 dB
SFO1 150.9178988 MHz
======== CHANNEL f2 ========
CPDPRG2 waltz16
NUC2 1H
PCPD2 80.00 usec
PL12 19.45 dB
PL13 19.50 dB
PL2 -1.00 dB
SFO2 600.1324005 MHz
F2 - Processing parameters
SI 32768
SF 150.9028090 MHz
WDW EM
SSB 0
LB 1.00 Hz
GB 0
PC 1.40
200
180
160
140
120
100
80
60
40
20
0
ppm

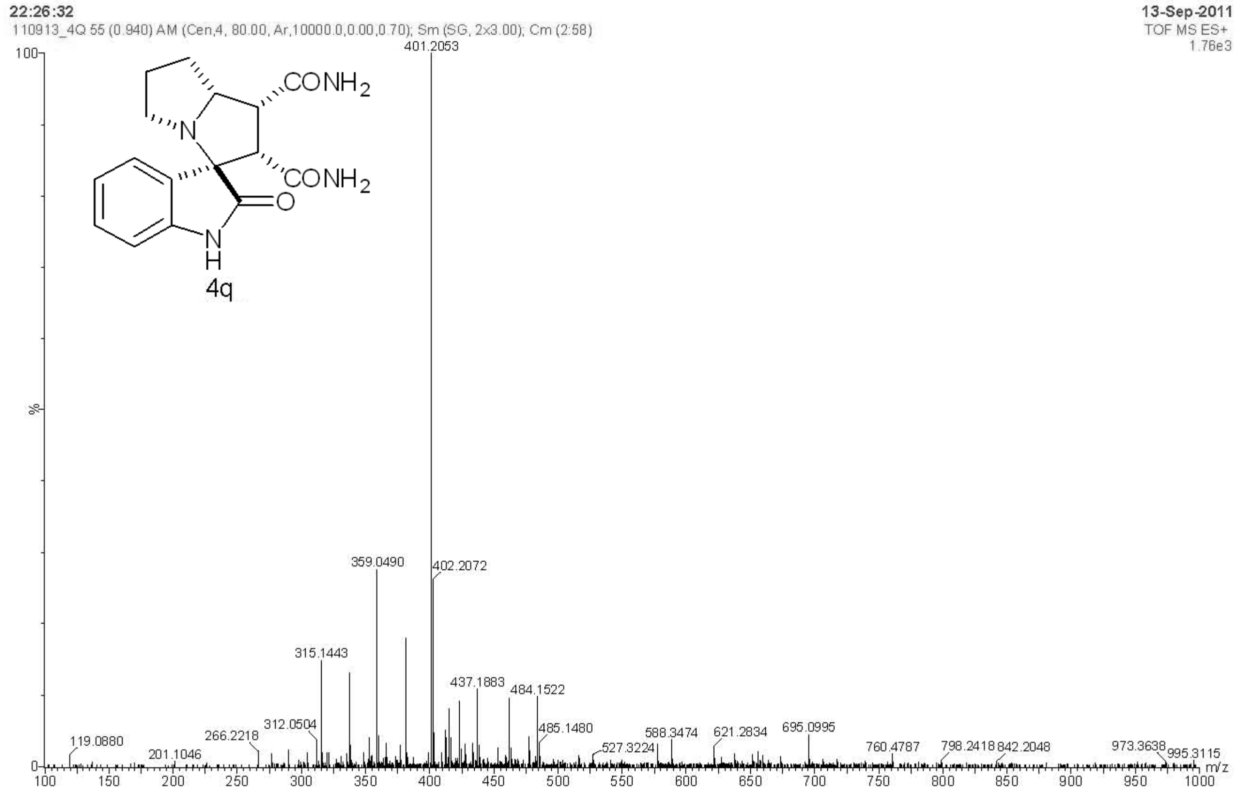
22:26:32
110913_4Q 55 (0.940) AM (Cen,4, 80.00, Ar,10000.0,0.00,0.70); Sm (SG, 2x3.00); Cm (2:58)
13-Sep-2011
TOF MS ES+
1.76e3
401.2053
CONH2
CONH2
O
N
H
N
4q
%
359.0490
402.2072
315.1443
437.1883
484.1522
312.0504
266.2218
485.1480
588.3474
621.2834
695.0995
119.0880
201.1046
527.3224
760.4787
798.2418
842.2048
973.3638
995.3115
m/z

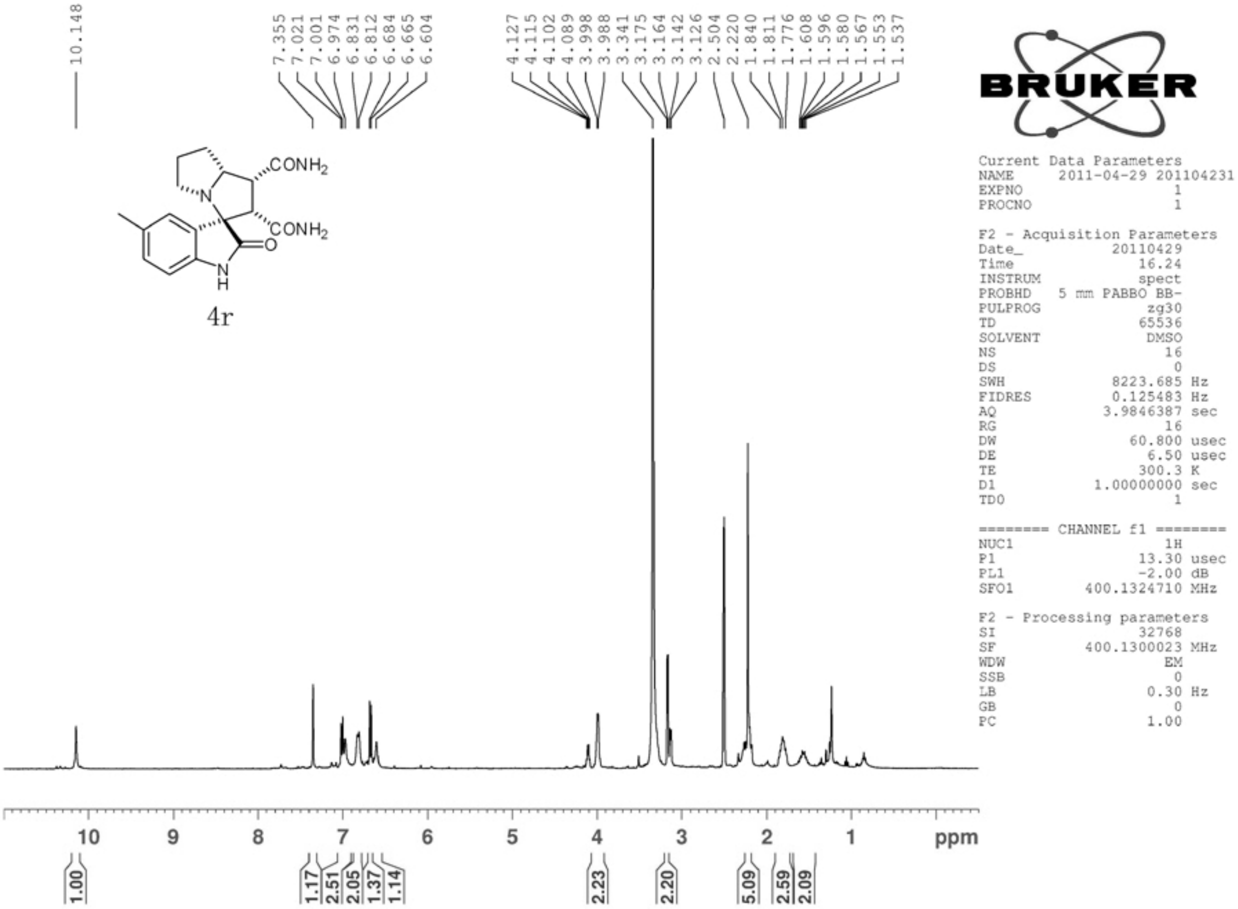
10.148
7.355
7.021
7.001
6.974
6.831
6.812
6.684
6.665
6.604
4.127
4.115
4.102
4.089
3.998
3.988
3.341
3.175
3.164
3.142
3.126
2.504
2.220
1.840
1.811
1.776
1.608
1.596
1.580
1.567
1.553
1.537
CONH2
CONH2
O
N
H
N
4r
Current Data Parameters
NAME 2011-04-29 201104231
EXPNO 1
PROCNO 1
F2 - Acquisition Parameters
Date_ 20110429
Time 16.24
INSTRUM spect
PROBHD 5 mm PABBO BB-
PULPROG zg30
TD 65536
SOLVENT DMSO
NS 16
DS 0
SWH 8223.685 Hz
FIDRES 0.125483 Hz
AQ 3.9846387 sec
RG 16
DW 60.800 usec
DE 6.50 usec
TE 300.3 K
D1 1.00000000 sec
TD0 1
======== CHANNEL f1 ========
NUC1 1H
P1 13.30 usec
PL1 -2.00 dB
SFO1 400.1324710 MHz
F2 - Processing parameters
SI 32768
SF 400.1300023 MHz
WDW EM
SSB 0
LB 0.30 Hz
GB 0
PC 1.00
ppm
1.00
1.17
2.51
2.05
1.37
1.14
2.23
2.20
5.09
2.59
2.09

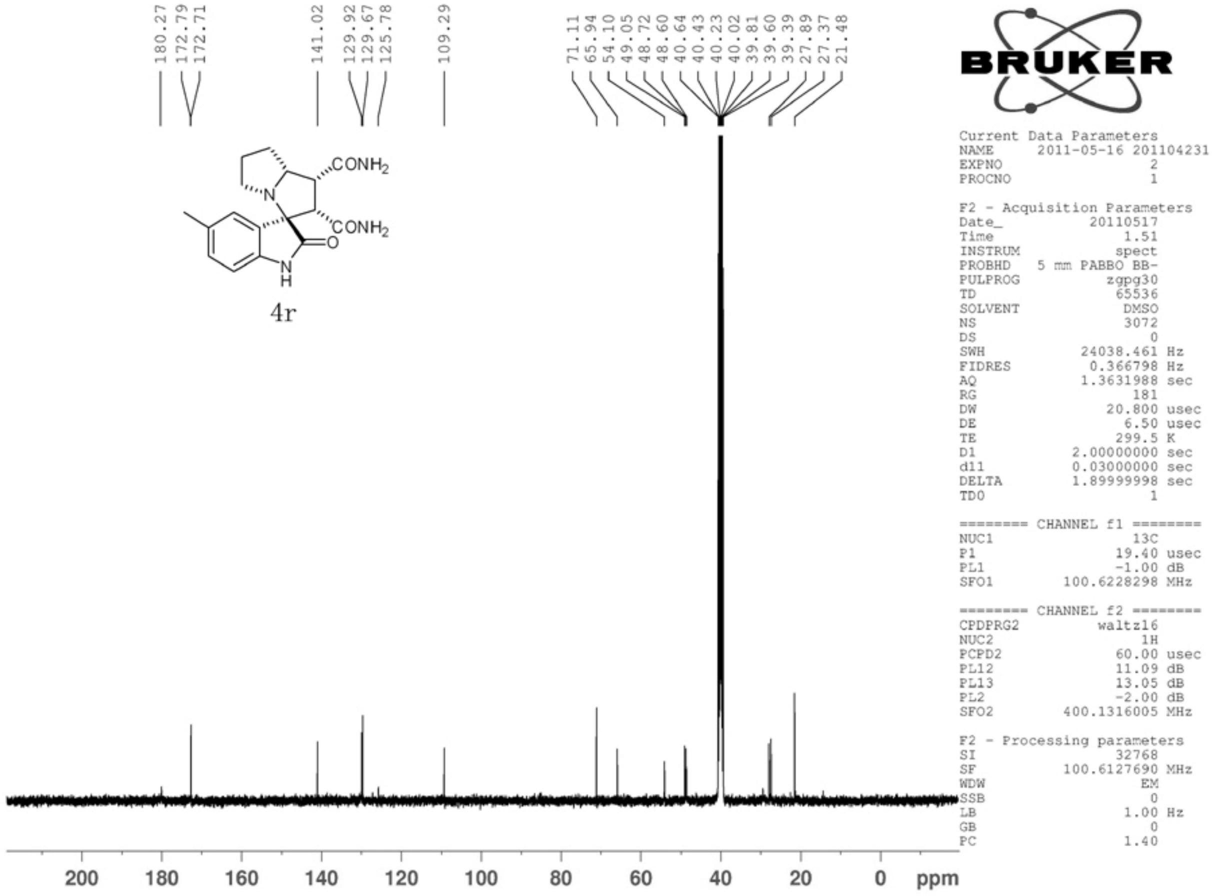
180.27
172.79
172.71
141.02
129.92
129.67
125.78
109.29
71.11
65.94
54.10
49.05
48.72
48.60
40.64
40.43
40.23
40.02
39.81
39.60
39.39
27.89
27.37
21.48
CONH2
CONH2
N
O
N
H
4r
BRUKER
Current Data Parameters
NAME 2011-05-16 201104231
EXPNO 2
PROCNO 1
F2 - Acquisition Parameters
Date_ 20110517
Time 1.51
INSTRUM spect
PROBHD 5 mm PABBO BB-
PULPROG zgpg30
TD 65536
SOLVENT DMSO
NS 3072
DS 0
SWH 24038.461 Hz
FIDRES 0.366798 Hz
AQ 1.3631988 sec
RG 181
DW 20.800 usec
DE 6.50 usec
TE 299.5 K
D1 2.00000000 sec
d11 0.03000000 sec
DELTA 1.89999998 sec
TD0 1
======== CHANNEL f1 ========
NUC1 13C
P1 19.40 usec
PL1 -1.00 dB
SFO1 100.6228298 MHz
======== CHANNEL f2 ========
CPDPRG2 waltz16
NUC2 1H
PCPD2 60.00 usec
PL12 11.09 dB
PL13 13.05 dB
PL2 -2.00 dB
SFO2 400.1316005 MHz
F2 - Processing parameters
SI 32768
SF 100.6127690 MHz
WDW EM
SSB 0
LB 1.00 Hz
GB 0
PC 1.40
200 180 160 140 120 100 80 60 40 20 0 ppm

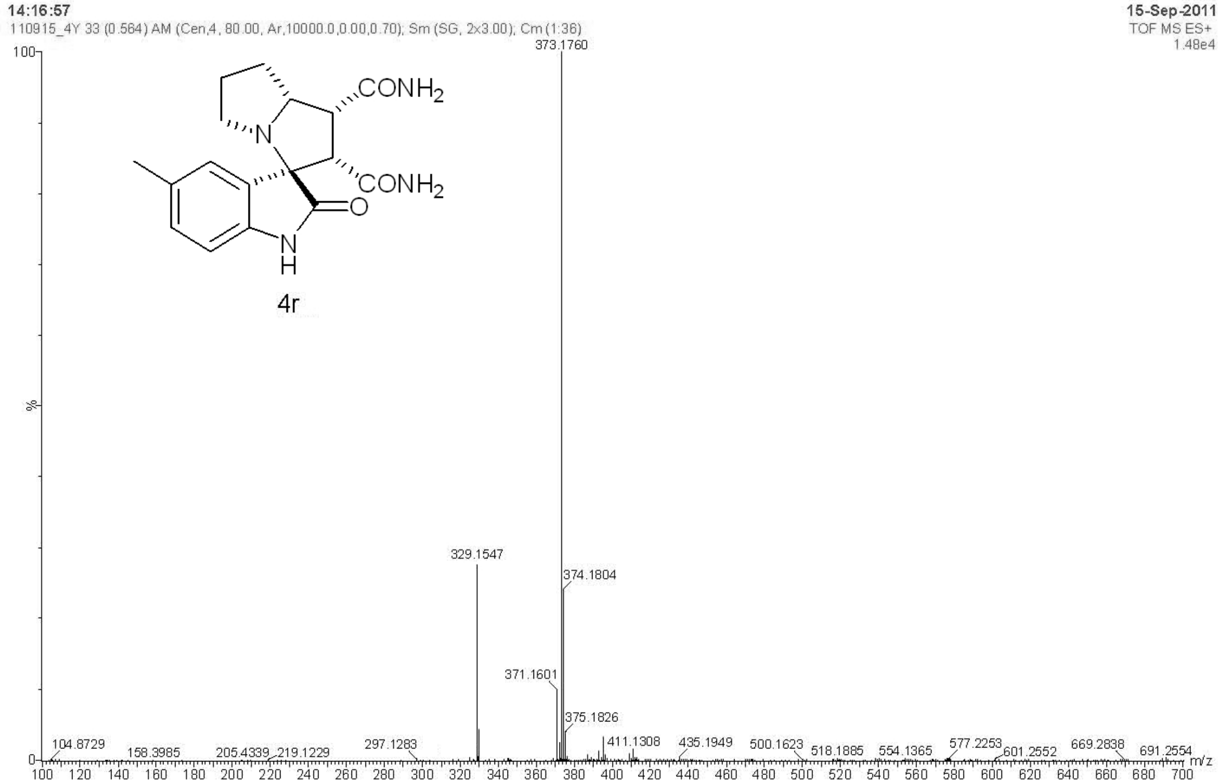
14:16:57
110915_4Y 33 (0.564) AM (Cen,4, 80.00, Ar,10000.0,0.00,0.70); Sm (SG, 2x3.00); Cm (1:36)
15-Sep-2011
TOF MS ES+
1.48e4
373.1760
329.1547
374.1804
371.1601
375.1826
104.8729
158.3985
205.4339
219.1229
297.1283
411.1308
435.1949
500.1623
518.1885
554.1365
577.2253
601.2552
669.2838
691.2554
m/z
%
100
0
CONH2
CONH2
N
O
N
H
4r
